# Exploring p53 isoforms: unraveling heterogeneous p53 tumor suppressor functionality in uveal melanoma

**DOI:** 10.1038/s41420-025-02891-1

**Published:** 2025-12-05

**Authors:** Laura Bartolomei, Yari Ciribilli, Samuele Brugnara, Francesco Reggiani, Gian Mario Moretta, Mariangela Petito, Elisa Marcaccini, Marianna Ambrosio, Carlo Mosci, Ulrich Pfeffer, Adriana Amaro, Paola Monti, Alessandra Bisio

**Affiliations:** 1 Department of Cellular, Computational and Integrative Biology (CIBIO), University of Trento, Via Sommarive 9, 38123 Trento, Italy; 2 SSD Gene Expression Regulation, IRCCS Ospedale Policlinico San Martino, Largo R. Benzi 10, 16132 Genoa, Italy; 3 Department of Experimental Medicine (DIMES), University of Genoa, Via L.B. Alberti 2, 16132 Genoa, Italy; 4 UO Neuro-oncology and Mutagenesis, IRCCS Ospedale Policlinico San Martino, Largo R. Benzi 10, 16132 Genoa, Italy; 5 Department of Ophthalmology, Ocular Oncology Center, E.O. Ospedali Galliera, Mura delle Cappuccine 14, 16128 Genoa, Italy; 6 Trento Institute for Fundamental Physics and Applications (TIFPA), Istituto Nazionale di Fisica Nucleare (INFN), Via Sommarive 14, 38123 Trento, Italy

**Keywords:** Eye cancer, Gene expression

## Abstract

Uveal melanoma (UM) is the most common intraocular tumor, and despite being rare, it accounts for nearly 13% of melanoma-related deaths. Indeed, patients with metastatic disease have typically survival rates of less than one year, with little improvement over the past few decades. Although *TP53* mutations are uncommon in UM, recent findings highlight a dysfunctional p53 pathway in this cancer. Given its crucial role in mediating DNA damage responses, we analyzed the p53 protein functionality and downstream target activation in a panel of UM cell lines in response to standard-of-care treatments (i.e., cisplatin and proton-beam irradiation). Although most of the analyzed cells retained a wild-type p53, we observed a wide range of p53 protein stabilization and targets’ activation. Recently, p53 isoforms have been recognized as modifiers of p53 activity, and their biology and functions depend on cellular context. We observed that UM cells express a broad spectrum of p53 isoforms, including Δ160p53α and Δ133p53β and the longer variants Δ40p53β and p53β. Interestingly, the down-regulation of the short p53 isoforms (Δ133/Δ160) revealed their contribution to promoting cell growth and in mitigating cell death triggered by standard-of-care therapies. Moreover, we verified the wild-type p53 status in a panel of 32 UM cases and analyzed the expression levels of p53 isoforms. Our results indicated a correlation between higher expression levels of Δ40p53α or Δ133p53γ isoforms and the development of more aggressive cancers. Our findings suggest that shorter p53 isoforms can promote cancer aggressiveness and therapy resistance, thereby providing crucial insights into UM pathogenesis.

## Facts

- Most UM cells have an aberrant p53 pathway and express a wide array of p53 isoforms, despite expressing a wild-type p53 protein.
- UM patients with a more severe phenotype were associated with higher levels of Δ40 and Δ133/Δ160 p53 isoforms or lower levels of p53β.
- Alterations affecting the p53 pathway or an unbalanced expression in the p53 isoforms may affect cancer aggressiveness and the responses to anti-cancer therapies.

## Introduction

Uveal melanoma (UM) is a rare tumor of the choroid, the ciliary body, or the iris with an annual incidence of 0.2–0.8 per 100,000 people and a North-South gradient across Europe; this latter feature correlates with the population prevalence of pale skin and light-colored eyes, which are two established risk factors [1, 2]. Despite the analogy with cutaneous melanoma, UV light is not associated with UM development since it is absorbed by the vitreous body and lens; consequently, the mutational burden and signature in UM differ from those found in skin cancer [3]. UM is the human tumor with the lowest mutational burden (17–30 mutations in coding sequences per genome and 0.5 mutations/DNA megabase) [4]. Frequently targeted driver genes include the G Protein Subunit Alpha Q or 11 (*GNAQ* and *GNA11*) and the tumor suppressor gene BRCA1-Associated Protein 1 (*BAP1*) [5, 6].

Due to its relatively low molecular complexity, the UM clinical behavior can be predicted with reasonable accuracy using the Liverpool Uveal Melanoma Prognosticator Online V3 (LUMPO3) tool [7, 8], along with a combination of pathological (e.g., tumor basal dimension) and cytogenetic (i.e., monosomy of chromosome 3 and amplification of chromosome 8q) criteria [9]. Molecular features, including the initiating mutation in *GNAQ* and *GNA11*, the metastasis driver mutations in *BAP1* [10], or the splicing factor *SF3B1* [11] or a gene expression-based classifier [12], also clearly distinguish metastatic risk classes. However, an accurate prognosis contrasts sharply with the lack of adjuvant therapies that could reduce the metastatic risk [13]. Furthermore, targeted- and immuno-therapies have shown minimal effects on metastatic UM and have, therefore, not been approved for the adjuvant setting. The rare and short-lasting responses to targeted- and immuno-therapy are explained by (i) the activation in *GNAQ* or *GNA11* mutated UMs of the two distinct signaling MAP-kinase [5, 14] and YAP/TAZ pathways [15], (ii) the low mutational burden, and (iii) the immunosuppressive environment of the anterior chamber of the eye [16] (which cannot be fully considered an immune privilege [17]) and the liver [18], the latter being the preferred site for UM metastasis. Currently, the only FDA-approved therapy for metastatic UM, Tebentafusp, a bispecific antibody that stimulates T cells to target Gp100-expressing UM cells [19], is expected to be approved for the adjuvant setting; however, it is limited to HLA-A*02:01-positive adult UM patients, unlikely to provide complete protection against metastasis.

The *TP53* gene, encoding for the tetrameric transcription factor p53, is the most frequently altered tumor suppressor in human cancers since the protein, through its ability to transactivate the expression of many downstream effector genes, controls several pathways whose dysregulation is highly selected in the carcinogenic process [20]. Disruption of the p53 pathway occurs mainly through missense mutations at the *TP53* locus, affecting the central sequence-specific DNA binding domain [21]. Still, other mechanisms of inactivation have been described in tumors, including the over-expression of the p53 negative regulators MDM2 and MDM4 [22].

Recently, it has become clear that the p53 functional scenario in cancer can also be altered by an unbalanced expression of p53 protein isoforms (i.e., p53α, p53β, p53γ, Δ40p53α, Δ40p53β, Δ40p53γ, Δ133p53α, Δ133p53β, Δ133p53γ, Δ160p53α, Δ160p53β, Δ160p53γ) resulting from a combination of alternative splicing, alternative promoters, and/or alternative translation start sites at the *TP53* locus [23]. Specifically, the transactivating forms p53α, p53β, and p53γ contain the entire transactivation domain, but p53β and p53γ lack the oligomerization domain; conversely, the Δ40, Δ133, and Δ160 p53 variants partially or entirely lack the transactivation domain. While p53β and p53γ have been shown to support tumor suppression (i.e., senescence induction by p53β [24] and p53γ association with a better prognosis in breast cancer patients [25]), Δ133 and Δ160 p53 variants have been associated with cancer aggressiveness, by stimulating proliferation, angiogenesis, and migration in several cancers [24, 26, 27]. The role of Δ40p53 isoforms in cancer is still debated [27, 28].

Initial reports in UM samples showed no (or very rare) mutations in the *TP53* gene analyzed by exome sequencing [29, 30]; conversely, disruption of the p53 pathway due to upstream or downstream mutations has been described [31], with over-expression of the p53 negative regulator MDM2 being a common mechanism in UM [32]. A study by Hussein and colleagues also showed the association of p53 protein over-expression in UM with some unfavorable histologic features, including invasion [33]. This observation was confirmed in a subsequent study that highlighted a correlation between high p53 protein expression and poor prognosis in UM [34]; accordingly, the inhibition of p53 expression was found to be associated with a decreased invasion of UM cell lines. Interestingly, Hajkova and colleagues recently identified a germline *TP53* mutation (I254V) in 2 patients with metastatic UM [35]. Finally, recent findings identified *TP53* as one of the significantly mutated genes (4%) in UM along with *BAP1*, *GNAQ*, *GNA11*, *SF3B1*, *EIF1AX*, and *PLCB4* [36].

All these studies suggest that p53 does not function properly as a tumor suppressor in UM, but the molecular basis of these observations remains to be elucidated. In this context, it is also known that the altered expression of p53 isoforms can affect the aggressiveness of cutaneous melanoma by interfering with p53-dependent responses through the interaction with full-length wild-type p53 [37].

Based on these premises, we decided to take a closer look at the p53 landscape of UM by analyzing 10 different cell lines and 32 UM samples. We first evaluated the *TP53* mutational status, showing that, in general, UM cell lines and patient samples express a wild-type p53 protein. The only exception was represented by the 92.1 cell line, which was characterized by the presence of the *TP53* K132T mutation along with two other wild-type copies of the *TP53* locus, an observation never reported before.

However, by analyzing the functionality of the p53 pathway in response to cisplatin and proton-beam irradiation, we showed that the UM cell lines displayed a wide range of p53-dependent responses, including cells with p53 protein stabilization and an intact activation of downstream targets and cells with an aberrant p53 pathway with a partial or poor stimulation of p53 protein and a corresponding inefficient upregulation of p53-dependent targets.

In parallel, we examined p53 isoforms expression in UM cell lines and patient samples, revealing the presence of different subtypes of p53 isoforms (i.e., p53α, p53β, Δ40p53α, Δ40p53β, Δ133p53β, and Δ160p53α); remarkably, the silencing of Δ133/160 p53 isoforms increased the sensitivity of UM cells to cisplatin. Additionally, UM patients with a more severe cancer phenotype (i.e., larger cancer size, metastatic disease, and high-risk subtype) were associated with higher levels of the oncogenic p53 isoforms (namely Δ40p53α or Δ133/160p53α) or lower levels of p53 isoforms that are considered tumor-suppressive (i.e., p53β).

Taken together, these results suggest that despite the wild-type p53 protein status in most UMs, alterations affecting the pathway (i.e., p53 stabilization and activation of downstream targets) or imbalanced expression of the shorter p53 isoforms may influence cancer aggressiveness and response to anti-cancer therapies in UM.

## Results

### All UM cell lines contain a wild-type p53 protein except for 92.1 cells

To assess the functional status of *TP53* in UM cell lines, we employed a well-established yeast-based assay (FASAY). By scoring the number of red colonies over the total number of transformants as a percentage, UPMM1, UPMM2, MEL270, MEL285, MEL290, UPMD1, UPMD2, OMM1, and OMM2.5 cells were characterized by a value of 7-17%, indicating the presence of a functional p53 protein (Supplementary Table 4). Sequencing confirmed the presence of a wild-type sequence along with various single nucleotide variants observed at p53 codons 72 and 213 (Supplementary Table 4). Conversely, the results from the 92.1 cell line suggested the presence of a *TP53* mutation (37.5%).

**Fig. 1:**
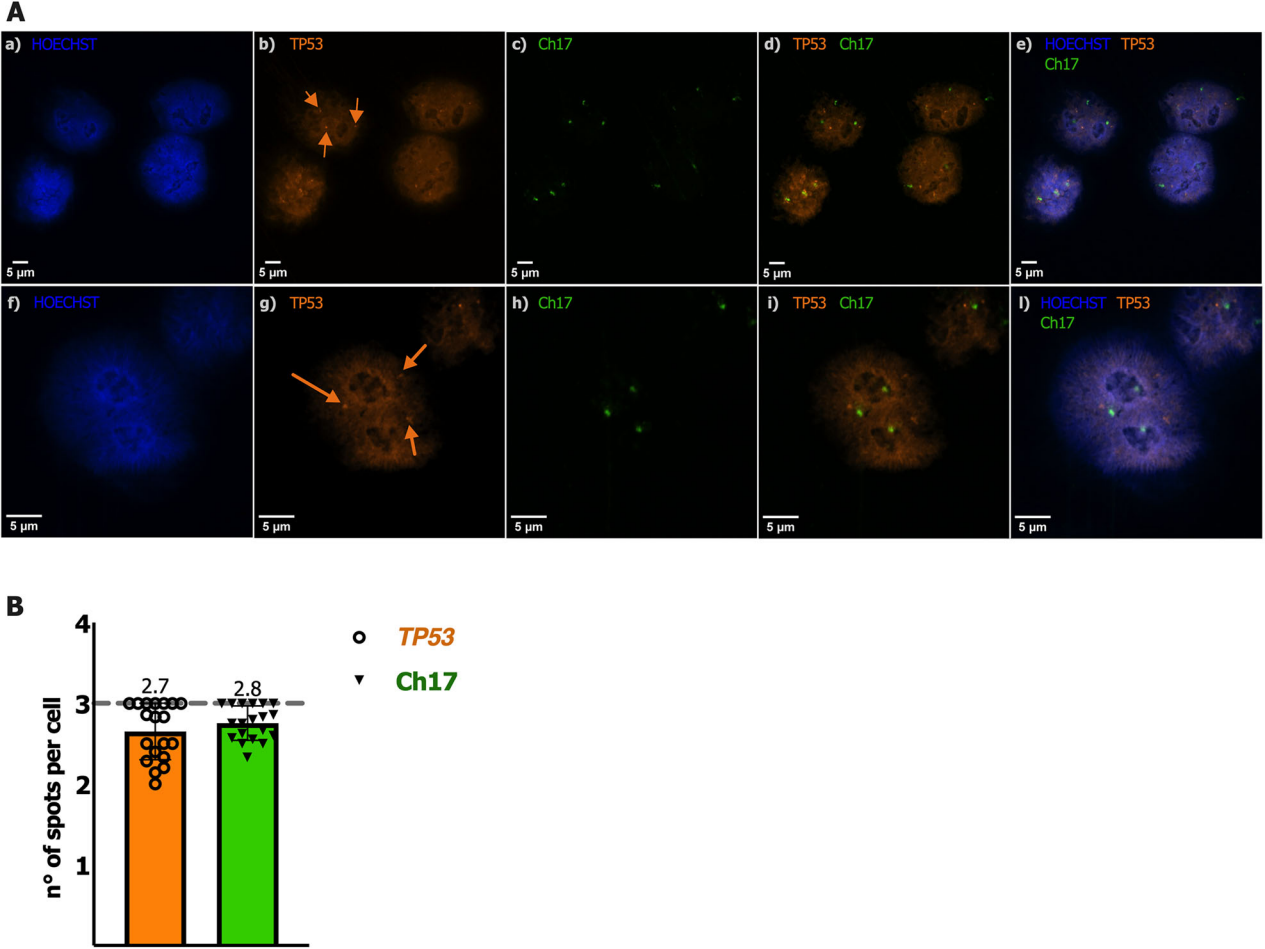
FISH analysis in 92.1 UM cell line. **A** FISH staining of 92.1 cell nuclei: (a,f) blue signal corresponding to Hoechst 33342; (b,g) orange signal corresponding to *TP53* gene which was highlighted by arrows; (c,h) green signal corresponding to chromosome 17 centromere; (d,i) merged images from b,g and c,h panels; (e,l) merged images of a,f, b,g, and c,h panels. **B** Relative quantification of the average number of green and orange spots per cell nucleus. Staining was done in a total of 120 cell nuclei.

By analyzing the *TP53* status of a cell line, it is expected that the presence of a non-functional *TP53* mutation in a heterozygous state typically results in approximately 50% of small red colonies. Therefore, our results suggested the presence of three copies of the *TP53* locus, one of which is affected by a non-functional *TP53* mutation. To confirm this hypothesis, we first sequenced the *TP53* coding sequence spanning the codons 42-375 by analyzing the PCR products derived from both cDNA and the red yeast colonies. The results revealed the presence of a *TP53* missense mutation at codon 132, causing the substitution of a Lysine with a Threonine (AAG > ACG, K132T). Then, we performed FISH analysis on this cell line, which revealed a trisomic gain for chromosome 17 and the presence of three copies of the *TP53* gene, located at 17p13.1 (Fig. 1A, B); the presence of the *TP53* K132T mutation, along with two wild-type copies of the *TP53* locus, has never been reported previously in the 92.1 UM cell line.

To better characterize the functional property of the *TP53* mutation identified in 92.1 cells, we took advantage of our yeast functional assay. The transactivation ability and dominant negative potential of the K132T p53 mutant protein were measured upon its expression in P21-5’, BAX A + B, PUMA, and MDM2P2C isogenic reporter strains. The results clearly showed the complete loss of transactivation activity (Supplementary Fig. 2A) and the dominant negative potential (Supplementary Fig. 2B) of the K132T mutation; in fact, this p53 mutant had a 0–1% transactivation ability in comparison with the wild-type p53 protein in all strains and temperatures tested, showing also a dominant potential (about 60%) on the wild-type p53 protein.

### UM cell lines present a functionally heterogenous p53-dependent pathway

**Fig. 2:**
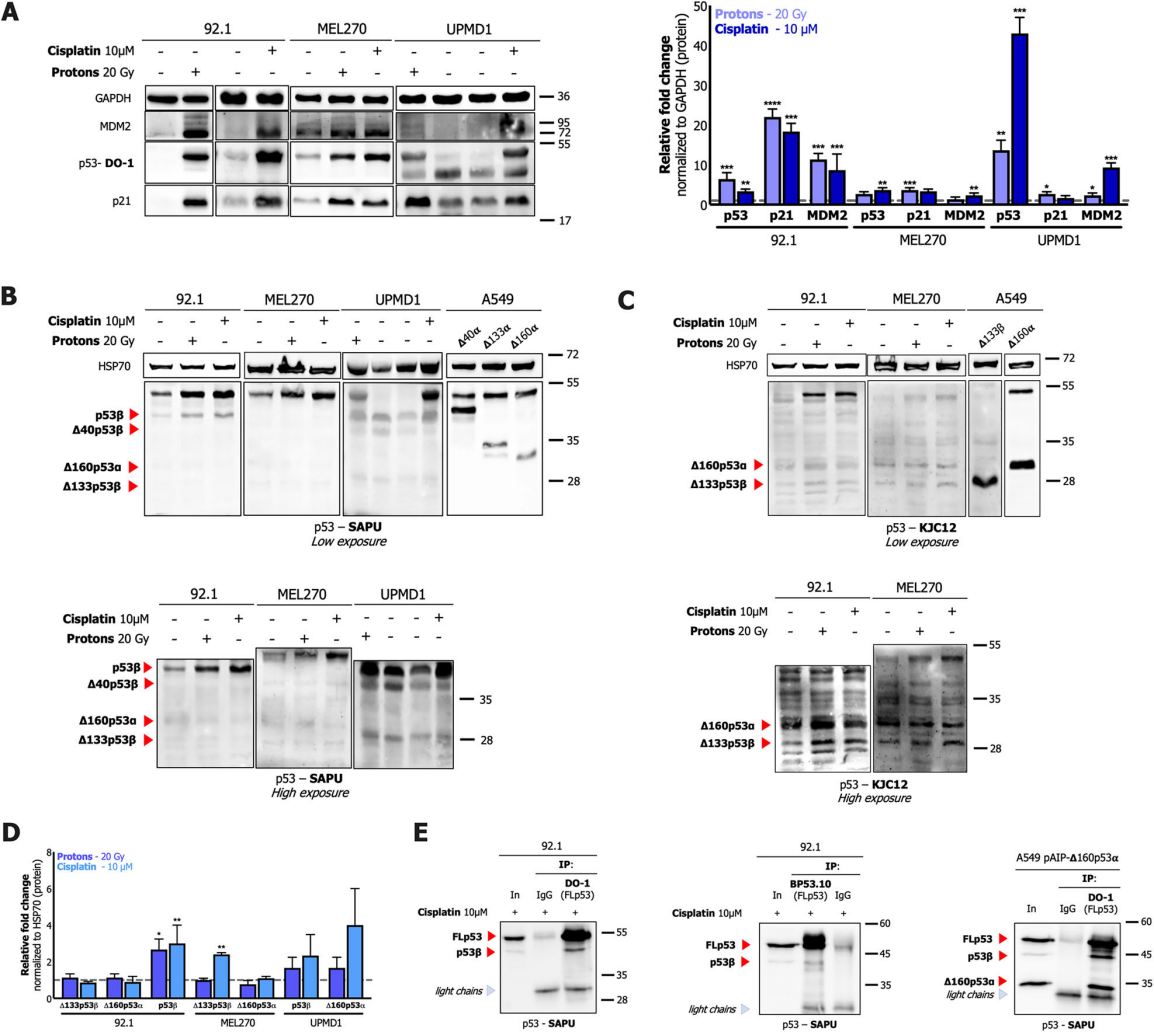
Characterization of p53 pathway functionality and p53 isoform expression in 92.1, MEL270, and UPMD1 primary UM cell lines by Western blot. **A** Left panels: a representative Western blot of p53 and p53 targets (p21, MDM2) expression in untreated (–) and treated (+) cells (10 μM cisplatin or 20 Gy proton irradiation). GAPDH expression was used as a reference protein. Right panel: relative quantification of the analysis; bars represent the average and the standard deviations of at least 3 biological replicates. A representative Western blot of p53 isoforms expression as in panel (**A**). SAPU (**B**) and KJC12 (**C**) primary antibodies, which can visualize all p53 isoforms, were used. During the detection, different exposure times allowed us to acquire, respectively, the signal coming from full-length and Δ40 p53 (upper panels low exposure) and shorter isoforms (bottom panels high exposure), respectively. A549 cells over-expressing either Δ40 or Δ133 or Δ160p53α/β were used as controls for the proper identification of the p53 isoforms. Specific isoform identification is highlighted with a red arrow tip and the name of the p53 isoform. In the case of the panels with high exposures, the over-expression controls were left out of the detection field to better identify the expressed p53 isoforms and aligned using the results with the low exposures. HSP70 expression was used as a reference. **D** Quantification of the analysis from panels B and C; bars represent the average and the standard deviations of at least 3 biological replicates. **E** Western blots derived from Co-IP to determine the interaction between different p53 isoforms and full-length p53α. Co-IP was performed using DO-1 antibody (left panel, to immuno-precipitate full-length p53α, β and γ) or BP53.10 (middle panel, to immuno-precipitate only p53α) on protein lysates derived from 92.1 cells treated with 10μM cisplatin and A549 clone over-expressing Δ160p53α isoform (right panel, again with DO-1 antibody). Input (5%) was used as positive control and mouse normal IgG as negative control. Western blots were performed using SAPU primary antibody to detect the presence of the different p53 isoforms. Whole panel: * = p < 0.05; ** = p < 0.01; *** = p < 0.001.

After observing that all UM cell lines, except 92.1 cells, harbor a wild-type p53 protein, we investigated the p53 pathway activation and the p53 isoforms’ expression in our panel of UM cell lines under stress-induced conditions (i.e., 10 μM cisplatin for 24 h or 20 Gy proton beam irradiation for 16 h). We characterized the expression of p53 isoforms at the protein level by using specific primary antibodies and controls (i.e., H1299 or A549 stably over-expressing Δ40/Δ133/Δ160p53 α, β, or γ). To define the ability of p53 to activate the downstream responses, for instance the induction of cell cycle arrest and apoptosis, we measured the mRNA and protein levels of known direct p53 target genes involved in these biological processes such as KILLER and PUMA (i.e., pro-apoptotic responses), p21 (i.e., cell cycle arrest activation), and MDM2 (i.e., p53 negative auto-regulation). The results highlighted a differential pattern of p53 stabilization and p53 target activation in response to treatment in the UM cell lines. Specifically, the primary 92.1 cell line exhibited a functional p53 pathway, as well as the UPMD1 cell line and, to a lesser extent, MEL270. Western blot analysis (Fig. 2A) and RT-qPCR (Supplementary Fig. 3A) showed p53 protein stabilization and significant induction of p21 and MDM2 p53 targets at the protein level, especially in the 92.1 cell line; PUMA and KILLER were also induced at the mRNA level (Supplementary Fig. 3A).

Interestingly, Δ160p53α and Δ133p53β appeared to be the shorter p53 isoforms that we found expressed in most of the analyzed panel of UM cell lines and they were slightly affected by the different treatments (i.e., only Δ133p53β in 92.1 after proton irradiation and in MEL270 after cisplatin treatment) (Fig. 2B–D). The presence of the long isoform p53β was also observed, whose expression was significantly increased after both treatments, only in the 92.1 cell line (Fig. 2B, D). UPMD1 cells showed high levels of p53β already at the baseline, which were partially increased upon treatments; moreover, these cells also expressed Δ40p53β and Δ133p53β (Fig. 2B, D).

Given the evidence that 92.1 cells contain a mutant p53, in addition to the wild-type protein, and that p53 is properly induced upon cellular stresses, we further investigated these findings by examining the localization of p53 protein using a cytoplasmic-nuclear fractionation protocol. We observed the stabilization of the transactivation competent FLp53α in both the cytoplasmic and chromatin fractions in cisplatin-treated samples (Supplementary Fig. 4A); to note, p53α appeared to be localized in the chromatin at a lower level than in the cytoplasm. This result is not entirely consistent with expectations: upon cytotoxic stress stimulation, p53 must enter the nucleus and selectively bind to chromatin to function as a transcription factor and to induce target genes as observed. The results could be due to the expression of the Δ160p53α isoform and its ability to bind the chromatin under unstressed conditions [37], affecting the detection of p53 protein; indeed, the Δ160p53α isoform showed in 92.1 cells a preferential localization in the chromatin-enriched fraction, as shown in Supplementary Fig. 4B, both at the baseline and after stimulation with cisplatin. Given the concomitant expression of FLp53α and other isoforms in UM cells, we also analyzed their potential interaction via Co-IP experiments followed by western blotting. Results indicated that in 92.1 cells upon the treatment with cisplatin (to stabilize FLp53α), endogenous FLp53α was able to interact with p53β directly (Fig. 2E, left and central panel). This observation was further confirmed by incubating the immunoprecipitated extracts with a β-specific antibody (79.3) (Supplementary Fig. 5A). Moreover, to confirm the potential also of shorter p53 isoforms to interact with FLp53α, we took advantage of the recently established clones over-expressing single p53 isoforms in A549 cells harboring a wild-type p53 protein (Moretta et al., in preparation). Interestingly, results starkly demonstrated that either Δ40p53α (Supplementary Fig. 5B), Δ133p53α (Supplementary Fig. 5C), or Δ160p53α (Fig. 2E) showed the ability to form complexes with FLp53α, potentially affecting its functions. Instead, we did not observe any direct interaction between Δ133p53β and FLp53α (Supplementary Fig. 5D), putatively due to the lack of the oligomerization domain.

**Fig. 3:**
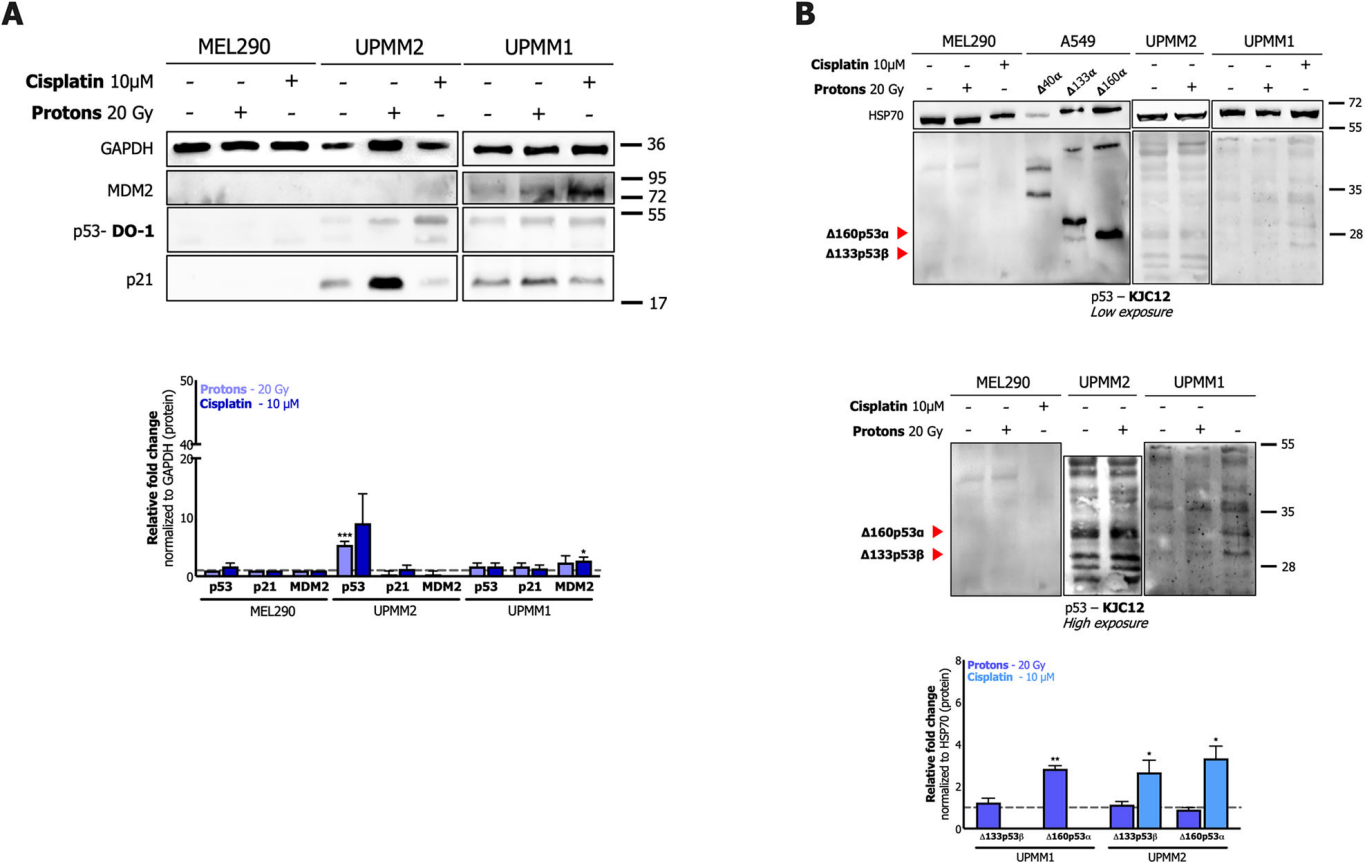
Characterization of p53 pathway functionality and p53 isoforms expression in MEL290, UPMM2, and UPMM1 primary UM cell lines by Western blot. **A** Upper panels: a representative Western blot of p53 and p53 targets (p21, MDM2) expression in untreated (–) and treated (+) cells (10 μM cisplatin or 20 Gy proton irradiation). The GAPDH expression was used as a reference protein. Bottom panel: relative quantification of the analysis; bars represent the average and the standard deviations of at least 3 biological replicates. **B** A representative Western blot of p53 isoforms expression as in panel A and detected as described in Fig. 2. Upper panels: low exposure; middle panels: high exposure; bottom panel: relative quantification of the analysis; bars represent the average and the standard deviations of at least 3 biological replicates. Whole panel: *= p < 0.05; ** = p < 0.01; *** = p < 0.001.

In contrast to the previous UM cell lines, MEL290, UPMM1, and UPMM2 cells showed poor or negligible functionality of the p53 pathway (Fig. 3). Remarkably, the DNA damage-inducing treatments failed to stabilize the p53 protein (except in UPMM2) and induce the corresponding p53 targets (Fig. 3A), despite a significant increase at the mRNA level (Supplementary Fig. 3B). The analysis of p53 isoforms expression revealed that UPMM1 and UPMM2 cells expressed both Δ160p53α and Δ133p53β isoforms, which were generally up-regulated in response to the treatments (i.e., both variants after cisplatin in the case of UPMM1 and only Δ160p53α after proton irradiation in UPMM2) (Fig. 3B).

Furthermore, UPMD2 and MEL285 primary UM cell lines did not show differential expression of p53 protein and downstream targets in response to the administration of DNA-damaging agent cisplatin, indicating an inactive p53 pathway. Western blot analysis highlighted elevated levels of p53, p21, and MDM2 protein expression in both cell lines under untreated conditions, with no further increase or significant changes upon treatment (Supplementary Fig. 6A), despite the upregulation of p53 target mRNAs (Supplementary Fig. 3C). Regarding p53 isoforms, MEL285 showed a slight treatment-dependent expression of the p53β isoform. Instead, the UPMD2 cell line expressed the Δ40p53β isoform, which was slightly but significantly induced by cisplatin treatment (Supplementary Fig. 6B).

We also analyzed the p53 pathway activation and isoforms’ expression in the metastatic OMM1 and OMM2.5 cells. In OMM1, the transcriptional levels of p21, PUMA, KILLER, and MDM2 were all upregulated after cisplatin treatment (Supplementary Fig. 3D). However, the p53 protein was not stabilized after either cisplatin treatment or proton irradiation. Regarding p53 targets, MDM2 was slightly increased upon DNA-damaging treatments, while p21 remained barely detectable (Supplementary Fig. 7A). The OMM2.5 cell line was derived from a liver metastasis of the patient from which the primary MEL270 cell line has been obtained. Interestingly, the former cell line already showed high levels of p53 expression in the untreated state, which was not accompanied by further stabilization. p21 was not expressed in these cells, and the observed increase at the protein level of MDM2 in correlation with the mRNA level suggested a p53-independent regulation (Supplementary Fig. 3D; Supplementary Fig. 7A). Concerning the p53 isoforms’ expression, an induction of Δ40p53β isoform was observed in the two metastatic cell lines (Supplementary Figs. 7B-D). Furthermore, in OMM1 cells, the expression of Δ160p53α and Δ133p53β isoforms was slightly increased upon both treatments (Supplementary Figs. 7B-D).

To further confirm the nature of the identified p53 isoforms in our panel of UM cell lines, we performed additional western blots using isoform-specific antibodies: β-specific (β-sheep antibody) and α-specific (BP53.10) (Supplementary Fig. 8). Results confirmed the expression of p53β, Δ40p53β, and Δ133p53β (Supplementary Fig. 8A–F), and Δ160p53α (Supplementary Fig. 8E) in a subset of UM cell lines as shown above with SAPU and KJC12 pantropic antibodies.

The observation of a non-functional p53 pathway in different UM cell lines stimulated us to evaluate whether the p53 activation/stabilization might occur at earlier time points, which we could have missed by analyzing the response only at 24 h. Results from 92.1, UPMM1, MEL285, and OMM2.5 cells (selected for the differential activation of the p53 pathway) treated with cisplatin for 8 and 24 h confirmed the previous results, being p53 stabilized and p21 target activated already 8 h post-treatment only in 92.1 cells (even to a lesser extent, as potentially expected). While in UPMM1 cells p53 was only slightly stabilized and p21 was not activated either at 8 or 24 h after treatment, in MEL285 and OMM2.5 cells, although the p53 levels were already higher at the baseline, p53 was not stabilized and p21 was not induced upon the treatments (Supplementary Fig. 9).

### *TP53* status in UM patient samples

The *TP53* functional status was also evaluated in 32 UM patient samples using the previously described experimental approach. Again, all UM samples were characterized by a percentage of 0.18–16.97% of red colonies, indicating the presence of a functional p53 protein. The Sanger sequencing confirmed the presence of a wild-type *TP53* sequence along with various polymorphism conditions at codon 72 (Supplementary Table 5).

### UM cell lines and patient samples differently express p53 isoforms

**Fig. 4:**
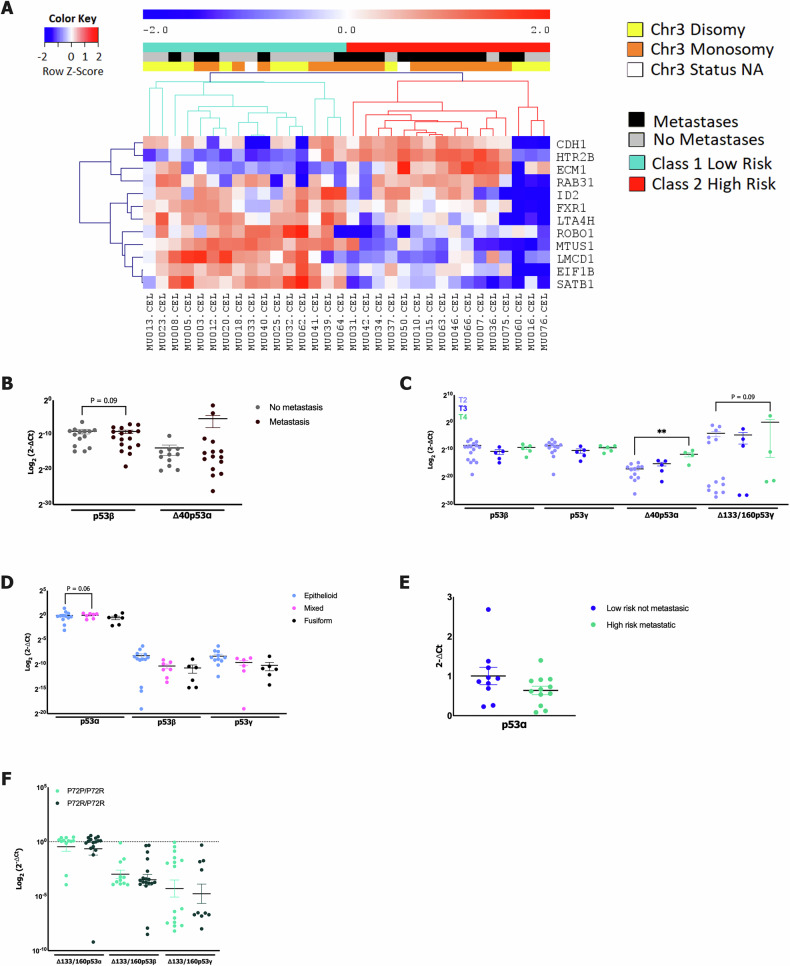
Relationship between p53 isoforms expression and clinical features in UM patient samples. **A** Hierarchical cluster heatmap showing the differential gene expression among the UM patients, according to the Harbor 15 genes signature. Presented are the 12 genes whose expression was relevant for discriminating the UM prognosis, while the 3 genes not included are reference genes (i.e., MRPS21, RBM23, and SAP130). The two clusters (red = Class 2 Risk, turquoise = Class 1 Risk) were enriched for cases with primary UM that developed metastases (black = metastases) and Chr3 monosomy (orange = monosomic) (right cluster) and with primary UM that did not develop metastases (gray = metastases-free) and Chr3 disomy (yellow = disomic) (left cluster). The expression values are reported by a color scale (blue = expression below the mean, red = expression above the mean, white = expression at the mean; the intensity is related to the distance from the mean). Cases with missing information = white. **B–E** Correlation of p53 isoforms expression with presence or not of metastasis, size of the tumor, histology of the tumor, and risk class defined with Harbor classification combined with the presence or not of metastasis. **F** Correlation of Δ133/Δ160p53 isoforms expression with rs1042522:C > G SNP status (*TP53* polymorphism at codon 72); the comparison was done between heterozygous and P72R homozygous UM patients. Whole panel: ** = p < 0.01.

To determine the basal expression levels of p53 isoforms in UM, we evaluated the mRNA expression of 9 p53 isoforms in 4 of the 10 cell lines (Supplementary Fig. 10) and 32 patient samples (Fig. 4) using a two-step nested quantitative PCR method adapted from our recently optimized protocol [38]. The 92.1 and MEL270 cell lines were selected based on the integrity of the p53 pathway, while OMM1 and OMM2.5 cells were analyzed for metastatic features. As shown in Supplementary Fig. 10A, short p53 isoforms (i.e., Δ133/160p53α, β, γ) retain moderate level of expression, even comparable to that of full-length p53 (except for MEL270) in line with what was previously observed at the protein level. Notably, Δ133/160p53β and γ were increased when comparing primary (MEL270) and metastatic (OMM2.5) cells (Supplementary Fig. 10B), suggesting again a putative pro-aggressive role of p53 short isoforms. Since material from UM biopsies was not suitable for protein analysis (as they originate from a previous research project with an ethical committee that only permitted the analysis of nucleic acids), we investigated only the mRNA expression of p53 isoforms in UM patient samples. First, we stratified the patient cohort (32 samples) using the Harbor classification (Fig. 4A), which is based on the expression of 15 specific genes and allowed us to distinguish between high- and low-risk UM [39, 40]. Combining the results of patient stratification with additional clinical data (Supplementary Table 2) (i.e., presence or absence of metastasis, TNM stage, and tumor cell type at the time of diagnosis), a correlation analysis with the p53 isoforms expression was performed (Fig. 4B–E). Interestingly, despite the lack of statistical significance we observed a tendency towards a lower expression of the p53β variant in tumors with high metastatic risk compared to low-risk tumors (Fig. 4B). This result is consistent with the expected role of p53β as an enhancer of p53α tumor suppressor activity at the promoter of target genes [41]. Conversely, increased levels of the oncogenic Δ40p53α appeared to be positively correlated with an increased size of the tumor (Fig. 4C, T4 > T3 > T2); also, Δ133p53γ showed a trend to increase in samples with the highest tumor stage. Interesting but not significant trends were also observed for the variation in expression of full-length p53 isoforms according to the histological features (i.e., cell type) of the tumor (Fig. 4D). Lastly, p53α mRNA (i.e., full-length p53) levels tended to be lower in high-risk metastatic patients compared to low-risk non-metastatic ones, leading to a decrease in the transcriptional activation of target genes, a scenario compatible with worse prognosis and more aggressive tumors (Fig. 4E).

It has been proposed that polymorphisms within the *TP53* internal promoter P2 may affect p53 transcriptional activity and influence the expression of the p53 isoforms produced by that regulatory region (Δ133/Δ160p53 isoforms) [42–44]. Therefore, we compared the expression levels of Δ133/Δ160p53 α, β, and γ isoforms with the *TP53* status at codon 72 (P72 vs. R72) in 30 out of 32 UM patients; the two homozygous P72P samples were excluded from the analysis due to the low frequency. Results showed a trend (even if not statistically significant) of Δ133/Δ160p53 isoform levels (particularly for β and γ isoforms), being higher in heterozygous UM patients in comparison with the homozygous P72R ones (Fig. 4F).

### The down-regulation of Δ160p53α isoform increases the sensitivity to anti-cancer treatments in 92.1 and MEL270 cell lines

**Fig. 5:**
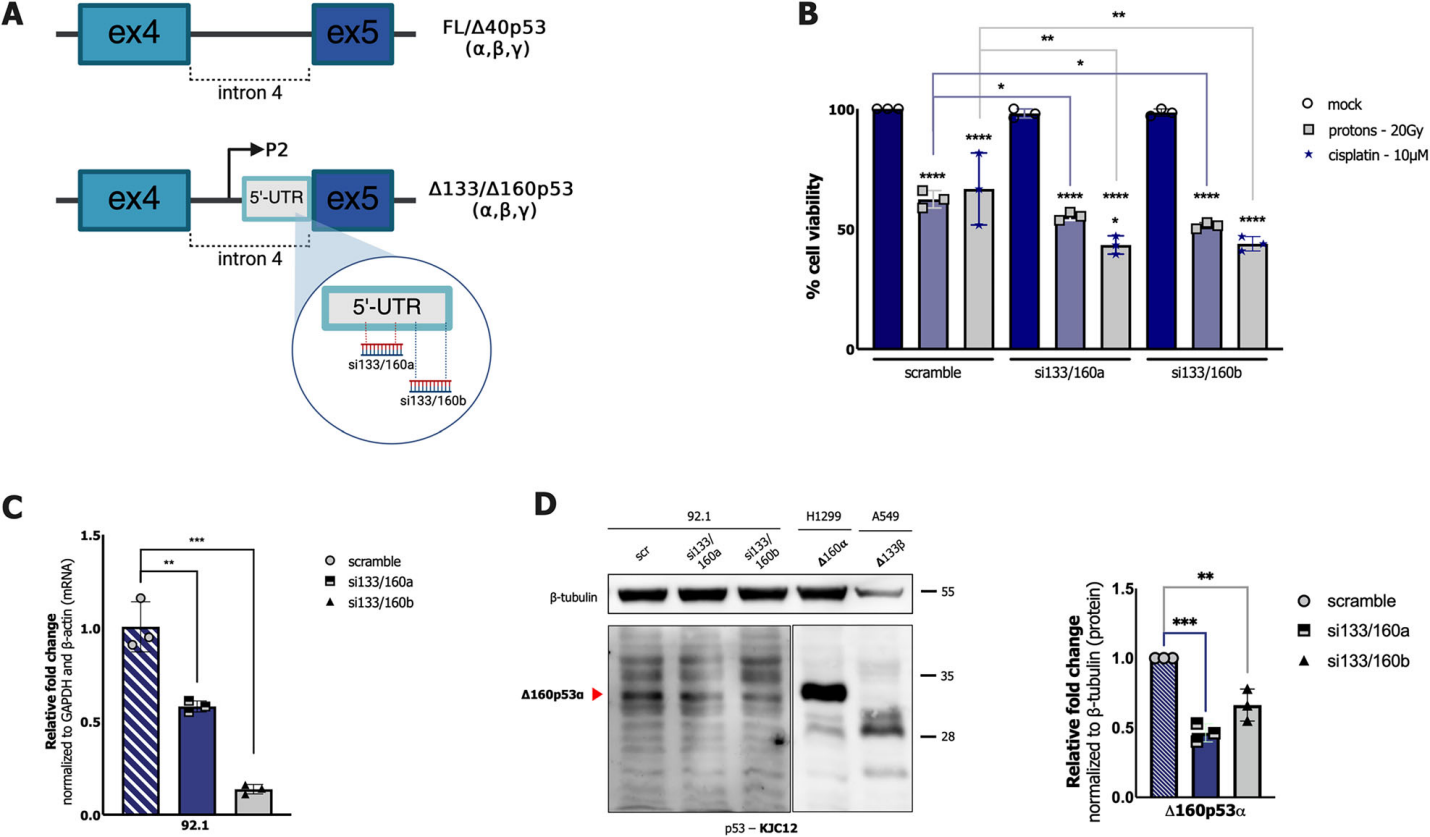
Combination of cisplatin treatment and proton beam irradiation with Δ160p53 and Δ133p53 isoforms silencing in 92.1 cells. **A** A schematic description about how 133/160a and 133/160b siRNAs have been designed according to Joruiz and Bourdon [23]; created with BioRender.com. **B** Percentage of cell viability of 92.1 cells evaluated by MTT assay to compare the effect of cisplatin treatment and irradiation with proton beam in cells silenced with specific p53 siRNAs or with a negative control (scramble). Twenty-four hours after the transfection with either 133/160a or 133/160b siRNAs, cells were treated with 10 μM cisplatin or irradiated with 20 Gy proton beam. DMSO administration was used as a negative control. Cell viability was measured 72 h post-transfection and 48 hours after treatment. **C** Evaluation of p53 isoforms silencing efficacy at transcriptional level by means of RT-qPCR in 92.1 cells. Specific primers (preamp-p53-Fw and TAp53-Rv) for Δ160p53 and Δ133p53 isoforms were used. GAPDH and β-Actin were used as reference genes. **D** Evaluation of p53 isoforms silencing efficacy at protein level by western blotting. p53 isoforms were detected by KJC12 pantropic antibody as indicated in Fig. 2 and β-tubulin served as reference protein. Lysates from H1299 as well as A549 cells over-expressing single p53 isoforms were used as controls and detected only at a lower exposure. On the right, the quantification of the detected bands; bars represent the average and the standard deviations of at least 3 biological replicates. Whole panel: * = p < 0.05; ** = p < 0.01; *** = p < 0.001; **** = p < 0.000001.

It is known that the deregulation of p53 isoforms expression can promote or inhibit tumor progression, with a prognostic value associated with the cell context. In particular, in cutaneous melanoma, the increased expression of Δ133p53β defines poor outcomes, and Δ160p53α over-expression can stimulate cell proliferation and migration [37]. As previously observed, 92.1, UPMM1, UPMM2, MEL270, UPMD1, and OMM1 cell lines showed Δ160p53α isoform expression, which remained stable between treated and untreated conditions in almost all of them (Fig. 2B–D, Fig. 3B; Supplementary Fig. 7B-C); in addition, 92.1, UPMM2, MEL270, and OMM1 cell lines showed concomitant expression of the Δ133p53β isoform. Therefore, we investigated whether the expression of these shorter p53 isoforms could affect the response to treatment, focusing on the 92.1 cell line. To perform p53 isoforms silencing, we used two different siRNAs, named si-133/160a and si-133/160b, which specifically target the 5’UTR of Δ133p53 and Δ160p53 mRNA variants (described in Fig. 5A) [23]; by using these experimental set-up we were able to clearly detect only Δ160p53α variant that was knocked-down both at RNA and protein levels, silencing either si-133/160a or si-133/160b in 92.1 cells (Fig. 5C, D). Then, we combined the silencing of shorter p53 isoforms with cisplatin treatment or proton irradiation in 92.1 cells; remarkably, cell viability was significantly reduced in this cell line (Fig. 5B). These results again support the involvement of Δ160p53 isoforms in cancer cell growth and the impairment of the response to therapy.

### p53 characteristics from publicly available omic data

To highlight the importance of our previous investigations, we analyzed available mutational and epigenetics data from published UM datasets to evaluate *TP53* genomic features in UM. Newell and coauthors have recently reported somatic genomic events affecting *TP53* and related pathways in patients diagnosed with UM; specifically, 17 patients with primary tumors from different sites have been studied (i.e., 2 ciliary bodies, 2 iris, 12 choroidal melanomas, and one unknown) [36]. Among them, 4 UM samples had single nucleotide variants (SNVs) or insertions/deletions (indels) in *TP53*, of which the majority led to a loss of p53 functionality (Supplementary Fig. 11A, Supplementary Table 6), while Copy Number Alterations (CNAs) and structural variants were detected in the p53 pathway of 14 patients (as reported in Supplementary Fig. 11 and Supplementary Table 2 of the work by Newell and collaborators [36]).

To evaluate the presence of epigenetic modifications affecting *TP53* in UM, we analyzed the largest publicly available multi-genomic dataset on UM samples, as published by Robertson and coauthors [45], and accessible as a TCGA dataset (https://portal.gdc.cancer.gov/). No somatic mutations in *TP53* were present among the samples considered in the TCGA UVM dataset, but 7 patients had CNAs on the *TP53* gene: 2 loss and 5 gain events, respectively (Supplementary Fig. 11B). Regarding the expression profile of *TP53*, no difference was observed between high and low-risk patients (chromosome 3 monosomic vs. disomic) as presented in Supplementary Fig. 11C. However, a difference in the mean methylation level of the *TP53* gene was evident in the former group, being the *TP53* methylation level higher in the high-risk patients versus the low-risk (Supplementary Fig. 11D, pvalue = 1.06 * 10^–7^, Welch two sample ttest); consistently, high-risk UM have generally overall increased genomic methylation levels compared to low-risk ones [45].

Taken together, it can be observed that *TP53* somatic alterations are part of the mutational landscape of a subset of UM samples and that the possible impact of *TP53* epigenetic alterations on UM evolution towards metastatic disease need to be further investigated.

## Discussion

*TP53* is a tumor suppressor gene that plays a critical role in maintaining genetic stability and preventing cancer development; indeed, its function is ubiquitously lost in most human cancers, mainly due to *TP53* gene mutations. Recent pieces of evidence suggest that the p53 tumor suppressive activity can be impaired in UM, but the molecular mechanisms underlying this effect have not been clarified yet [31, 34].

Here, we analyzed the *TP53* mutational status in 10 UM cell lines using a yeast functional assay (FASAY), confirming that all cell lines, except for 92.1, exclusively exhibited wild-type p53 protein (Supplementary Table 4). Interestingly, we described for the first time by FISH analysis a trisomic asset at the *TP53* locus of the 92.1 cell line with the presence of a mutant allele (K132T) along with two wild-type alleles (Fig. 1A, B). Then, with the aim of better characterizing p53 functionality, we investigated the p53 pathway activation in a wide panel of UM cell lines (i.e., primary UPMM1, UPMM2, 92.1, MEL270, MEL285, MEL290, UPMD1, and UPMD2 cells along with metastatic OMM1 and OMM2.5 cells) both in basal conditions and in response to standard UM therapies (i.e., cisplatin and proton-based radiotherapy).

Our findings revealed significant differences in terms of p53 stabilization and p53 targets’ activation, as well as p53 isoforms’ expression. High p53 levels following the treatments were observed in 92.1, MEL270, and UPMD1 primary UM cell lines, coupled with significant activation of the p53 pathway, as evidenced by increased levels of p21, MDM2, PUMA, and KILLER (Fig. 2A, Supplementary Fig. 3A). These results, together with the analysis in 92.1 cells of p53 protein internalization in the nucleus and binding to the chromatin in response to cisplatin treatment (Supplementary Fig. 4A), confirmed that the identified *TP53* mutation (K132T, present as a single copy) does not affect the overall p53 ability to function as a transcription factor in response to cellular stresses despite its classification by our yeast-based functional assays as a loss of function p53 mutant protein and able to act as dominant-negative over one single wild-type p53 protein (Supplementary Fig. 2A,B).

**Fig. 6:**
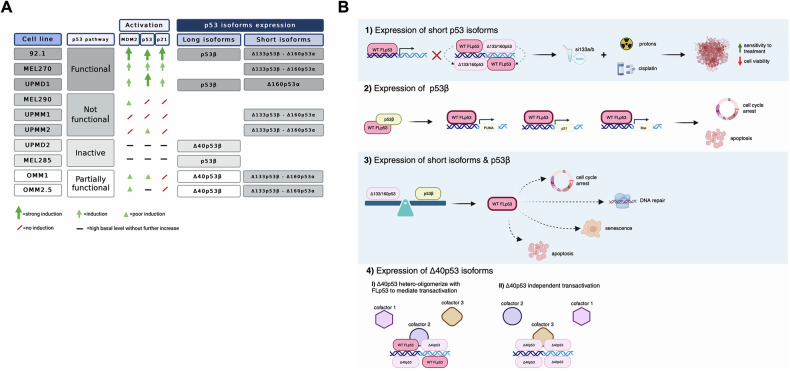
The p53 tumor suppressor pathway showed functional heterogeneity in uveal melanoma. **A** A summary of the p53 pathway functionality and p53 isoform expression in our panel of 10 UM cell lines. **B** A graphical representation of the impact of p53 isoforms altered expression on the p53-dependent transcriptional regulation and the influence on downstream pathways. Created with BioRender.com.

Regarding the other cell lines, we were able to highlight poor or almost no p53 pathway activation (Fig. 3A; Supplementary Fig. 6A; Supplementary Fig. 7A; Supplementary Fig. 3B-D); indeed, p53 and the expression levels of its targets did not display great differences between treated and untreated conditions. A summary of the p53 pathway functionality from the different UM cell lines is presented in Fig. 6A.

Previously, it has been shown that p53 isoforms, in particular the low-molecular-weight ones, are expressed in several malignancies [46, 47] and can contribute to defining cutaneous melanoma aggressiveness [37]. In this study, we confirmed the expression of different types of p53 isoforms among the UM cell lines, either induced or not by the cellular stresses we used; indeed, these distinct patterns of p53 isoforms’ expression might also account for the heterogeneity in p53 pathway functionality that we described.

Recently, it has been shown that Δ160p53α short isoform can stimulate the proliferation and migration of cutaneous melanoma cells, being described as the most variable isoform in terms of expression and prone to modification by DNA-damaging agents. Δ160p53α is imported into the nucleus where it exhibits chromatin binding capability, potentially influencing gene expression and biological processes [37]. Accordingly, in our panel of UM cell lines, the analysis highlighted Δ160p53α expression in most of them (i.e., 92.1, MEL270, UPMD1, UPMM1, UPMM2, and OMM1) (Figs. 2D, 3B, and Supplementary Fig. 7), appearing as the most variable and widely expressed isoform. In 92.1 and MEL270 cells, the expression of Δ160p53α did not vary between basal conditions and after treatment with DNA-damaging agents; in these cell lines very strong to mild p53 pathway responses were previously observed (Fig. 2A). Conversely, UPMD1, UPMM2, and UPMM1 cells that display elevated Δ160p53α protein levels as a consequence of the treatments with cellular stresses (Figs. 2D, 3B), exhibited a heterogeneity in p53 responses, being significant as p53 induction with corresponding targets only in UPMD1 cells (Fig. 2A vs. 3A). Recent studies highlight that Δ133p53 and Δ160p53 short isoforms may inactivate full-length p53 by tetramerization and fast aggregation, potentially exerting a “dominant-negative effect” or promoting cancer aggressiveness by “gain of function” mechanism [48]. By using a control cell line over-expressing specific p53 isoforms, we were able to confirm the direct interaction between both Δ133p53α (Supplementary Fig. 5C) and Δ160p53α (Fig. 2E) with FLp53α. These observations are in line with the recent study by Zhao and co-workers that showed how Δ133p53 and Δ160p53 can form oligomers with FLp53α negatively influencing its functions by their high propensity to aggregation [49]. Also Tomas and colleagues demonstrated a negative impact of Δ133p53 and Δ160p53 on FLp53α by altering its active conformation [50]. Our results also indicate that the high Δ160p53α expression might alter the p53 tumor suppressor functionality, hindering its capability to induce cell responses such as cell cycle arrest and apoptosis. However, the nuclear import and chromatin binding capability of Δ160p53α form (Supplementary Fig. 4B) did not affect the p53 response in 92.1 cells (Fig. 2A).

Besides, we observed the expression of Δ133p53β variant in 92.1, MEL270, UPMM1, UPMM2, and OMM1 cell lines, whose protein levels were increased after cisplatin treatment in MEL270 and UPMM2 cells (Figs. 2D, 3B). Previously, the presence of the Δ133p53β isoform was predicted to define poorer outcomes in patients with cutaneous melanoma [38], also in virtue of its ability to antagonize p53-mediated apoptosis, inducing the expression of anti-apoptotic BCL-2 family members [51]. Thus, it is possible that even in UM, the expression of this p53 isoform favors cancer cell growth and aggressiveness, in turn counteracting the effect of therapies.

To delve deeper into all these observations, a siRNA-based approach has been used to silence the expression of both Δ133p53 and Δ160p53 isoforms in the 92.1 cell line. Although we were able to confirm the silencing of only Δ160p53α, interestingly, the rate of cell viability was slightly but significantly reduced when combining Δ160p53α down-regulation with cisplatin and proton beam administration, indicating that reduced levels of at least this p53 variant make the UM cells more responsive and sensitive to anti-cancer treatments (Fig. 5B) (Fig. 6B, panel 1).

Some of the UM cells exhibited also the expression of long isoform p53β (Fig. 2D; Supplementary Fig. 6B); in 92.1 cells as well as in UPMD1 and MEL285 and cell lines, our analysis pointed out the presence of a signal corresponding to a protein with a 47 kDa molecular weight (shared by p53β and Δ40p53α) and the usage of α- and β-specific antibodies allowed us to identify p53β expression (Supplementary Fig. 8A–D, F). Functionally, p53β has been shown to form a complex with the p53α variant, boosting its activity on the *BAX*, *P21*, and *PUMA* promoters, acting as an enhancer [41, 52]. Indeed, we showed by Co-IP a direct physical interaction between FLp53α and p53β in 92.1 cells (Fig. 2E and Supplementary 5A). Regarding its clinical relevance, while this isoform represents a marker of good prognosis in colon cancer [24], AML (acute myeloid leukemia) [53], and renal cell carcinoma [54], the expression of p53β was instead associated with worse survival in ovarian cancers with a wild-type *TP53* status [55], with reduced cutaneous melanoma-specific survival [56], and with tumor progression in multiple myeloma patients [57]. An increased expression of p53β was also found in most cutaneous melanoma cell lines in response to cisplatin treatment [48]. Given that in other cancer cells p53β appears to regulate the transcriptional activity of endogenous wild-type p53, we can suggest the existence of a similar effect also in UM cell lines, since we showed induction or high basal levels of p21 in these cell lines (Fig. 2A, Supplementary Fig. 6A) (Fig. 6B, panel 2). The UPMD1 cells showed a reduced p21 induction with respect to 92.1 cells (Fig. 2A); this could be due to the presence in this cell line of elevated levels of Δ160p53α (Fig. 2D), given that it is known that the expression balance among p53 isoforms with opposite functions (i.e., tumor suppressive vs. oncogenic, Fig. 6B, panel 3) has a relevant impact on the overall p53 transcriptional activity [23].

In addition to the p53β variant, the other long isoform whose expression has been identified is Δ40p53β, being present in the primary UPMD2 cell line and in the metastatic cells OMM1 and OMM2.5; interestingly, the high levels of Δ40p53β isoform were also inducible by the treatment with cisplatin or 20 Gy proton-beam irradiation (Supplementary Figs. 6B,7D,8C-D). As mentioned above, Δ40p53 can modulate p53 target gene expression in both positive and negative manner; in fact, its role is dependent on the full-length p53:Δ40p53 ratio and on the cellular context in which it elicits a tumor suppressor or pro-tumorigenic function (Fig. 6B, panel 4) [46, 58]. However, limitations of the mentioned previous studies might rely on the use of over-expression models and the lack of knowledge on C-terminal variants Δ40p53β and Δ40p53γ [28].

A summary of the described p53 isoforms’ expression in UM cell lines is presented in Fig. 6A.

In the present study, we also analyzed the *TP53* gene status and the pattern of expression of p53 isoforms in 32 UM patient samples. The whole panel of UM samples presented a wild-type *TP53* coding sequence (Supplementary Table 5), consistent with the low *TP53* mutation rate found in UM (around 4%) [36]. Interestingly, we showed that p53 isoforms (i.e., Δ40p53α or Δ133/Δ160p53) previously associated with cancers with more aggressive characteristics [23, 46, 47] tended (even if in a few cases not reaching the standard statistical significance) to be associated in UM samples with clinical parameters linked to a worse prognosis, such as tumor size, the increased risk of developing metastases, or the histological features (Fig. 4B–E).

Splicing of the primary transcripts could also be affected by somatic mutations of the splicing factor SF3B1 that in UM are associated with intermediate risk of delayed metastasis [59]; however, the analyses of the effects of these mutations have not identified *TP53* splicing variants or different isoform expression patterns [60]. Further analyses on MEL202 cell line, carrying the SF3B1^R625G^ mutation, might provide valuable insights into the identification of specific effects of this mutation; though, MEL202 cells strongly respond to PARP-inhibitors [61] and an involvement of the p53 pathway cannot be excluded.

Lastly, we explored the association between the well-known *TP53* gene polymorphism (rs1042522:C > G) within *TP53* internal promoter P2 and the expression levels of Δ133/Δ160p53 isoforms in UM patients. Since higher of Δ133/Δ160p53 isoforms levels were associated with cancer aggressiveness, our results indicated a trend in the presence of the Proline at codon 72 along with the production of this set of p53 isoforms (Fig. 4F). Our results are in line with several observations (including cutaneous melanoma [62]) associating the presence of this amino acid at codon 72 with a worse prognosis (lung and breast cancer among others [63]), even if other fewer reports indicated the opposite.

In conclusion, our current findings underscore a dysregulation of the p53 pathway in UM mainly linked to altered expression of p53 isoforms, which have been implicated as pro-tumorigenic in other malignancies such as cutaneous melanoma; our results lay the groundwork for subsequent studies aimed at elucidating their specific role in this type of cancer. Many questions remain unsolved: how do p53 long and short isoforms’ expression impact therapy resistance and cancer cell aggressiveness in UM patients? Which molecular mechanisms are involved? Why do primary and metastatic UM cell lines express a different pattern of p53 isoforms, and how does this influence the patient’s prognosis? The dissection of these questions might help to identify additional therapy targets for UM in the future.

## Materials and methods

### Cell lines

The UM cell lines UPMM1 (RRID:CVCL_C299), UPMM2 (RRID:CVCL_C294), 92.1 (Human uveal melanoma, RRID:CVCL_C8607), MEL270 (RRID:CVCL_C302), MEL285 (RRID:CVCL_C303), MEL290 (RRID:CVCL_C304), UPMD1 (RRID:CVCL_C297), UPMD2 (RRID:CVCL_C298), OMM1 (RRID:CVCL_6939), and OMM2.5 (RRID:CVCL_C307) were used since they recapitulate the genetic alterations commonly observed in UM (Supplementary Table 1). UPMM1, UPMM2, UPMD1, and UPMD2 cells were obtained from Prof. Michael Zeschnigk (University Hospital Essen, University Duisburg-Essen, Germany). 92.1, MEL270, MEL285, MEL290, OMM1, and OMM2.5 were kindly provided by Prof. Martine J. Jager (University of Leiden, The Netherlands). NCI-H1299 and A549 cells over-expressing single p53 isoforms were generated using lentiviral infections (pAIP vector-based) and selection of pooled clones with Puromycin antibiotic as previously described by Tadijan and co-workers [37] and by Moretta and colleagues (unpublished), respectively. Cells were cultured in RPMI 1640 medium (Gibco, Life Technologies, ThermoFisher Scientific, Milan, Italy) and supplemented with Fetal Bovine Serum (FBS, 10%, Gibco), Penicillin-Streptomycin (1%, Gibco), and L-Glutamine (2 mM, Gibco) (37 °C, 5% CO_2_). In the case of NCI-H1299 and A549 cells over-expressing p53 isoforms, 1μg/ml Puromycin was added to the complete medium. UM cells were kept in culture for no more than 1 month from thawing. All cell lines used in this study were authenticated using Short Tandem Repeats (STR) profiling within the last three years. All experiments were performed with mycoplasma-free cells.

### UM patient samples and clinical data

UM patients were enrolled at the Galliera Hospital (Genoa, Italy). Mutational status, somatic mutations, cytogenetic alterations, gene expression profiles, and clinical follow-up were available (GSE51880, GSE27831; Supplementary Table 2).

### RNA extraction, cDNA synthesis, PCR amplification, and sequencing

RNA extraction was performed using the RNeasy Plus Mini Kit (Qiagen, Milan, Italy), following the manufacturer’s protocol. Briefly, cells were harvested and subjected to lysis with Buffer RLT containing beta-mercaptoethanol; the lysate was homogenized and cleared of genomic DNA and cellular debris by passing through a QIAshredder spin column. The RNA was then captured on a silica-based membrane and purified by sequential washing with a series of buffers to remove contaminants; the RNA was then eluted with RNase-free water and the concentration was determined by NanoDrop spectrophotometer. cDNA synthesis was performed by using hexamer primers (cDNA synthesis Kit, Biotech Rabbit, Berlin, Germany).

### Yeast cells culture conditions and transformation

Yeast cells were grown in YPDA medium (1% Yeast extract, 2% Peptone, 2% Dextrose, 200 mg/L Adenine) or in selective medium containing dextrose as carbon source and adenine (5 mg/L for FASAY or 200 mg/L for yeast p53 functional assay) without leucine or leucine plus tryptophan based on the selection of the expression vectors (Merck/Sigma-Aldrich, Milan, Italy). The manipulation of yeast cells was performed as previously described [64].

### Evaluation of *TP53* coding sequence status in UM cell lines and patient samples by FASAY (Functional Analysis of Separated Alleles in Yeast) assay and sequencing

FASAY assay was used to define the *TP53* functional status [65]. This assay exploits homologous recombination in the yIG397 *S. cerevisiae* strain between the terminal ends of p53 PCR product and a double-digested vector, generating a plasmid expressing the entire p53 coding sequence. The yIG397 strain contains at genomic level the *ADE2* reporter gene involved in the biosynthetic pathway of adenine under the control of a human p53 response element (RE). When yeast cells express a functional p53 (i.e., wild-type) that is able to bind and transactivate from the RE, they produce the phosphoribosyl aminoimidazole carboxylase enzyme (ADE2) and form white colonies on plates containing a limiting amount of adenine; conversely, cells containing a mutant p53 that is unable to transactivate the reporter gene grow as small red colonies on the same plates due to the lack of expression of ADE2 reporter and the accumulation of a colored intermediate in the adenine biosynthetic pathway. Specifically, to perform the assay, the p53 coding sequence corresponding to codons 42-375 was amplified using primers P3 and P4 [65] and Pfu DNA Polymerase (Biotech Rabbit); cDNA from UM cell lines or samples was used as template. The yIG397 strain was co-transformed with the unpurified PCR product from UM cell lines or patient samples, together with the pRDI22 double-digested (HindIII/StuI, New England Biolabs, Euroclone, Milan, Italy) vector (50 ng). The cells were grown for 3 days at 30 °C on selective plates containing 5 mg/L adenine and scored as red colonies over the total number of transformants (red/red plus white as percentage, %); the presence of a temperature-sensitive *TP53* mutation was also evaluated by streaking yeast colonies at three different temperatures (24 °C, 30 °C, and 37 °C).

The PCR products were purified (GenUP PCR Cleanup Kit, Biotech Rabbit) and sequenced with primers P5 (5’-TGGCCATCTACAAGCAGTCA-3’) and P6 (5’-GGGCACCACCACACTATGTC-3’) by the Sanger method (BMR Genomics, Padoa, Italy); electropherograms were interpreted by using the ApE v3.1.6 software. Yeast colony PCR was also performed from at least four red colonies derived from the FASAY assay on the 92.1 cell line by using 2x Hot-Start PCR Master Mix (Biotech Rabbit); the PCR amplification was preceded by incubation at 95 °C for 8 min to disrupt the yeast cell wall. The PCR products were analyzed at the molecular level as previously described (BMR Genomics).

### Construction of the mutant *TP53* K132T allele by two-step PCR mutagenic approach and cloning in yeast expression vectors

A pair of complementary 30-mer oligonucleotides (used as forward and reverse primers) was synthesized with the mutated base adjacent to the central position of the oligonucleotide (K132T forward: 5’-TCC CCT GCC CTC AAC **ACG** ATG TTT TGC CAA -3’; K132T reverse: 5’-TTG GCA AAA CAT **CGT** GTT GAG GGC AGG GGA-3’) [64]. The forward and reverse primers were used in two separate PCR reactions (Pfu DNA Polymerase) and paired with the P4 and P3 primers, respectively, using the pLS76 plasmid containing the wild-type *TP53* coding sequence as template. As before, the yIG397 yeast strain was co-transformed with the unpurified PCR products together with the pRDI22 digested vector, taking advantage of the sequence homology of the PCR fragments. The resealed plasmid DNA (pLS-based, LEU2 selection marker, constitutive p53 expression under *ADH1* promoter) was recovered from yeast yIG397 transformants by genomic extraction and expanded in *E. coli*. The presence of the specific *TP53* mutation (K132T) was confirmed at the molecular level by DNA sequencing (BMR Genomics). The *TP53* mutation K132T was reconstructed in the galactose-inducible pTSG-based vector (TRP1 selection marker, *GAL1,10* promoter) by SgraI/StuI double-digestion and subsequent ligation (New England Biolabs) from the pLS-based vector.

### Yeast functional reporter assay

The yLFM-P21-5’, yLFM-PUMA, yLFM-MDM2, and yLFM-BAX A + B strains were used to evaluate the transactivation ability of the *TP53* K132T mutation in comparison with wild-type p53 protein; all strains are isogenic except for the different response element located upstream of the *LUC1* luciferase reporter gene [66]. pRS314 (*TRP1*) and pRS315 (*LEU2*) were used as empty vectors. Briefly, yeast strains were transformed with pTSG-based (*TRP1*) expression vectors along with the empty vector pRS314. The yLFM-P21-5’ strain was used to evaluate the dominant potential (i.e., the ability of the K132T p53 mutant protein to inhibit the activity of the wild-type p53 protein). The pLS-based vector and the pLS89 plasmid (*TRP1* and *GAL1,10* promoter), expressing the K132T p53 mutant protein and wild-type p53 protein, respectively, were co-transformed into the yeast strain and compared, as reporter activity, to single wild-type p53 protein expression (pLS89 plus pRS315). Yeast transformants were resuspended in a selective liquid medium (i.e., without tryptophan or tryptophan plus leucine) containing raffinose as a carbon source and galactose using a 96-well transparent plate. Reporter expression was measured after 8 h of incubation at 30 °C or 37 °C. Luciferase assays were performed in white 96-well plates using the Bright-Glo™ Luciferase Assay Kit (Promega, Milan, Italy) and detected using the Mithras LB940 multi-plate reader (Berthold Technologies, Bad Wildbad, Germany). Relative light unit (RLU) values were normalized to the OD_600_ absorbance of each culture measured from the 96-well transparent culture plates using the same plate reader. Results were expressed in terms of fold induction using the RLU values obtained from transformants with the empty vector(s) as a reference. Fold induction was used to evaluate the percentage of transactivation ability and dominant potential of the *TP53* K132T mutation compared with wild-type p53.

### Analysis of *BAP1* coding sequence status

The coding sequence of the *BAP1* gene in UM cell lines was defined by Sanger sequencing (BMR Genomics); specifically, B1 (5’-ATGAATAAGGGCTGGCTGGA- 3’) and B5 (5’-TCACTGGCGCTTGGCCTT-3’) primers were used to amplify the 2.1 Kb BAP1 coding sequence (transcript variant 1 from RefSeq NM_004656.4) with Pfu DNA Polymerase (Biotech Rabbit). Prior to sequencing, PCR products were purified using QIAquick Gel Extraction (Qiagen). B2 (5’-CACCTTCAGCACATGCAGCC- 3’), B3 (5’ -CTCAGGGCTGAAACCCTTGG), and B4 (5’-CTCCAAGGTGCTTTTTGGAG) primers were used for sequencing. Sequences were analyzed as described above.

### FISH analysis on 92.1 UM cell line

FISH (Fluorescence in Situ Hybridization) was performed on whole cells, using a two-color hybridization to quantify the number of *TP53* gene copies in cancer cell nuclei from the 92.1 cell line. To fix cells, 92.1 line was detached from culture flasks and resuspended in pre-warmed 75 mM KCl for 15 min at 37 °C. The corresponding pellets were first resuspended in ice-cold fixative solution (1:3; acetic acid:MetOH) and kept at –20 °C for 2 h and then in maintenance solution (2:5; acetic acid:MetOH); cells were then spotted on glass slides.

FISH analysis was performed using the probes Vysis LSI TP53 SpectrumOrange/CEP 17 SpectrumGreen Probe according to the manufacturer’s instructions (Abbott Molecular, Des Plaines, IL, USA). Nuclei were counterstained with BD Hoechst 33342 solution, and fluorescence signals were captured using the Nikon ECLIPSE Ti2-confocal microscope at the CIBIO Advanced Imaging facility.

### Treatments of UM cell lines

UM cell lines were seeded in T25 flasks to reach 80% confluence and then treated with cisplatin or exposed to proton beam irradiation. Cisplatin (Selleckchem, Aurogene, Rome, Italy) treatment was performed by adding the chemotherapeutic agent directly to the cell culture at a concentration of 10 μM; cells used as mock were treated with DMSO. Proton beam irradiation (148 MeV/70 mA; 2 Gy/minute) was performed at the Proton Therapy Center (PTC) in Trento, with a total dose of 20 Gy.

After the cisplatin treatment or the proton beam irradiation, cells were kept in the incubator at 37 °C in a humidified atmosphere with 5% CO_2_; cell pellets were collected and processed for further analysis, such as Western blot and RT-qPCR analysis after cisplatin treatment (8 and 24 h) or after proton beam irradiation (16 h). These time points were chosen to appreciate the activation of the p53 pathway better.

### RNA extraction, cDNA synthesis, and evaluation of p53 targets expression by Real-time PCR

RNA was harvested using TRI Reagent® and the Direct-zol RNA Miniprep Kit (Zymo Research, Aurogene). cDNA was prepared using the RevertAid^TM^cDNA Synthesis Kit (Thermo Fisher Scientific, Milan, Italy). To analyze p53 isoforms RNA levels by RT-qPCR in UM samples, an additional step was performed by treating the RNA with 1U RNase-free DNase I enzyme (Thermo Scientific). Quantitative RT-PCR was performed on 25 ng of template cDNA, using the qPCRBIO SyGreen master mix (PCRBiosystems) and run on the QuantStudio5 Real-Time PCR system (Applied Biosystems, Thermo Fisher Scientific). *GAPDH* and *ACTB* were used as housekeeping genes; relative fold change was calculated using the ΔΔCt method as previously described [66]. Primer pairs were tested both for specificity and efficiency as earlier [67], and primer sequences are reported in Supplementary Table 3.

### Western blot analysis

Western blot was performed as previously reported [68]. Briefly, total protein cell extracts were obtained by lysing the cells with NP-40 buffer (1% NP-40, 150 mM NaCl, 50 mM Tris-HCl pH=8) supplemented with 1X protease inhibitors (PI) (Roche, Milan, Italy). Proteins were quantified using the BCA method (Pierce, ThermoFisher Scientific), and then 50 μg of proteins were loaded on 10–12% polyacrylamide gels for SDS-PAGE. After the separation, the proteins were transferred onto nitrocellulose membranes (Amersham, Merck), which were kept in blocking solution (5% skimmed milk-PBS-0.1% Tween solution) for 1 h at room temperature. Membranes were then incubated over-night at 4 °C with the following specific antibodies (diluted in 1–3% skimmed milk-PBS-0.1% Tween solution): HSP70 (C92F3A-5, Santa Cruz Biotechnology, DBA Italia, Milan, Italy), GAPDH (6C5, Santa Cruz Biotechnology), β-tubulin (3F3-G2, Santa Cruz Biotechnology), Vinculin (7F9, Santa Cruz Biotechnology), Histone H3 (ab18521, Abcam, Prodotti Gianni, Milan, Italy), p53 (DO-1, Santa Cruz Biotechnology), p53beta (79.3 and β-sheep, both provided by Dr. J.C. Bourdon, University of Dundee, Scotland, UK), pantropic p53 (KJC12 and SAPU, both from Dr. J.C. Bourdon), p53alpha (TSR and BP53.10, both from Dr. J.C. Bourdon), MDM2 (MA113, Thermo Fisher Scientific), and p21 (EPR362, Abcam). A scheme showing the epitopes recognized by p53-specific antibodies is presented in Supplementary Fig. 1. HRP-conjugated secondary antibodies were diluted in 1% skimmed milk-PBS-0.1% Tween solution and were obtained from Merck/Sigma-Aldrich (anti-mouse and -rabbit) or Jackson ImmunoResearch Europe (Prodotti Gianni) (anti-sheep). Detection was performed with ECL Select Reagent (Amersham) using the UVITec Alliance LD2 (UVITec Cambridge, UK) imaging system. Quantifications were performed using the Image J tool by analyzing the area of interest normalized for the specific reference protein.

### Cytoplasmic-nuclear fractionation

The MNase (Microccoccal Nuclease)-based subcellular fractionation protocol was performed as previously described [69]. Briefly, cellular pellets were lysed on ice using NBS (Nucleus Separation Buffer, 10 mM KCl, 1.5 mM MgCl_2_, 10 mM HEPES, 0.34 M Sucrose, 10% Glycerol, 1 mM DTT, 0.1% Triton X-100, supplemented with 1X Protease Inhibitors). Then, cytoplasmic fractions were collected and pellets containing the nuclei were resuspended in NBS supplemented with 1 mM CaCl_2_ and 2000 gel units/ml MNase and left at 37 °C for 10 min. After centrifugation, the remaining pellets were resuspended in NBS supplemented with 600 mM NaCl and left rotating at 4 °C overnight. The day after, samples were centrifuged, and supernatants, corresponding to the chromatin-enriched fractions, were collected.

### Co-ImmunoPrecipitation (Co-IP)

A549 cells over-expressing Δ40p53α, Δ133p53α, Δ133p53β, and Δ160p53α isoforms, and 92.1 cells were seeded in two P100 mm dishes, each condition. 92.1 cells were treated with 10 μM cisplatin to stabilize endogenous p53 protein. Twenty-four hours post-seeding or post-treatment, Co-IPs were conducted as previously described [69] using CHAPS lysis buffer (0.5% CHAPS, 50 mM Tris-HCl pH 7.4, 150 mM NaCl, 10% Glycerol with the addition of 1X PI), Protein G-Dynabeads (Life Technologies), 2 μg of DO-1 (to immunoprecipitate FLp53), BP53.10 (to immunoprecipitate FLp53α), or normal mouse IgG (as a control, Santa Cruz Biotechnology) antibodies for the IP, and the detection of enriched p53 isoforms bound by FLp53α was performed by western blotting as above using the pantropic SAPU and the β-specific 79.3 primary antibodies. Input was obtained as 5% of each lysate prior to IP. When appropriate, HSP70 was used as a reference protein for the inputs.

### RNA interference

Small interfering RNAs (siRNAs, Integrated DNA Technologies, IDT, Coralville, IA, USA) and the transfection reagent INTERFERin® (Polyplus-Transfection, Euroclone) were used to reduce the expression of target RNAs as previously described [69]; a scrambled non-targeting siRNA was used as a control. Briefly, cells were seeded in 6-well plates with a concentration of 400,000 or 600,000 cells/each well for the 92.1 or MEL270 cells, respectively. Twenty-four hours after the seeding, 25 nM siRNA duplexes targeting both Δ133 and Δ160 p53 isoform mRNAs (named si133/160a and si133/160b) [23] were diluted in 200 μL of medium without serum or in Opti-MEM® medium (Life Technologies); 12 μL of INTERFERin® reagent were added to the siRNA duplexes. The mix was homogenized by vortexing for 10 seconds and incubated for 10 min at room temperature before adding it to each well; gene silencing was measured between 48 h and 72 h for mRNA and protein levels.

### MTT assay

Colorimetric MTT assay [3-(4,5-dimethythiazol-2-yl)-2,5-diphenyl tetrazolium bromide] was used to quantify cell viability among different conditions tested. 15,000 92.1 cells were seeded in a 96-well plate and were left growing in RPMI supplemented with 5 mM Sodium Pyruvate solution overnight. The day after, cells were treated with 25 nM siRNA duplexes that were diluted in 50 μL of medium without serum or in Opti-MEM® with the addition of 1 μL INTERFERin® reagent. Twenty-four hours after transfection, 10 μM cisplatin was added to each well; DMSO-treated cells were used as a control. After 48 h of treatment, 10 μl of MTT reagent solution was added to each well, and the plate was kept at 37 °C for 3 h. After incubation, the medium was discarded, and cells with formazan crystals were solubilized by adding 100 μl of DMSO. Then, the plates were gently shaken for 10–15 min, and formazan absorbance levels were detected at 570 nm with the Varioskan LUX Multimode Microplate Reader (Thermo Fisher Scientific).

### Evaluation of p53 isoforms expression in UM cell lines and patient samples

To distinguish 9 different *TP53* isoforms, a quantitative PCR was performed according to the recently developed two-step nested qPCR method we adopted and slightly modified, as previously described [38]. A total of 25 ng (for full length and ∆40p53) or 50 ng (for ∆133p53) of cDNA was used for the pre-amplification steps using Go-Taq MasterMix (Promega, Madison, WI, USA). qPCR was then performed using 1:400 (for full length and ∆40p53) or 1:100 (for ∆133p53) diluted pre-amplified PCRs using the qPCRBIO SyGreen master mix (PCRBiosystems) and was run on the QuantStudio5 Real-Time PCR system (Applied Biosystems, Thermo Fisher Scientific). Relative fold change was calculated using the ΔΔCt method as mentioned above. Results were analyzed with Design & Analysis software v2.7.0 (Thermo Fisher Scientific), normalized with Ct values for total p53, and antilog values of 2^−∆Ct^ were presented as bars or dots using GraphPad Prism 9 (GraphPad Software, La Jolla, CA, USA). Primer pairs were tested for specificity and efficiency, and the sequences are reported in Supplementary Table 3.

### Gene expression profiling

Microarray gene expression data were analyzed in R/BioConductor. Quantile normalization was performed using RMA. The association of metastatic disease and chromosome 3 status with prognostic molecular classes was assessed for 32 UM samples derived from GSE27831 and GSE22138 using R and Bioconductor with ComplexHeatmap [70]. The gene expression based on Harbor’s prognostic classifier [39, 40] was used for the hierarchical clustering of UM cases by applying average linkage and Pearson distance measure as described earlier [71].

### Omics analysis on already published NGS UMs dataset

RNAseq data of the UVM TCGA dataset was extracted from the Combat gene expression file (work by Piaggio and colleagues [3]); gene mean methylation data for the same dataset was downloaded from the Broad GDAC Firehose website (http://gdac.broadinstitute.org/). Gene expression and methylation data were reported with raincloud plots using the introdataviz R package. Welch two sample t test pvalues were computed with R; the CNA plot was made with the karioploteR R package [72] and based on CNA array data of the UVM TCGA dataset (http://gdac.broadinstitute.org/). *TP53* lollipop plot was created with MutationMapper [73].

### Statistical analysis

The paired or unpaired Student’s t test or one-way ANOVA test were used when appropriate. p values are shown as following: (* p < 0.05; ** p < 0.01; *** p < 0.001). Analyses were performed using PRISM v 9.4 (GraphPad).

## Supplementary information

Supplementary Figure Legends

Supplementary Figure 1

Supplementary Figure 2

Supplementary Figure 3

Supplementary Figure 4

Supplementary Figure 5

Supplementary Figure 6

Supplementary Figure 7

Supplementary Figure 8

Supplementary Figure 9

Supplementary Figure 10

Supplementary Figure 11

Supplementary Table 1

Supplementary Table 2

Supplementary Table 3

Supplementary Table 4

Supplementary Table 5

Supplementary Table 6

Original data

## Data Availability

Data sources and handling of the publicly available datasets used in this study are described in the Materials and Methods. Further details and other data that support the findings of this study are available from the corresponding authors upon request. The full length uncropped original western blots are shown in the ‘Supplementary Material’.
