# Supplementary material for: Exploring p53 isoforms: unraveling heterogeneous p53 tumor suppressor functionality in uveal melanoma
Source: Cell Death Discov. 2025 Dec 5;12:39. doi: 10.1038/s41420-025-02891-1 (PMC12827457; doi:10.1038/s41420-025-02891-1)
Supplement: Supplementary file 1 — Supplementary Figure Legends [file 41420_2025_2891_MOESM1_ESM.docx]

**Supplementary Figure 1. Schematic representation of the *TP53* gene structure and the 12 human p53 isoforms adapted from Joruiz and Bourdon** ^23^**.** The color of the protein mirrors that of the encoding exon. On top is represented canonical p53α with its functional domains: the TransActivation domains (TA-1, TA-2), the Proline-Rich Domain (PRD), the DNA-binding domain, the Hinge Domain (HD), the Oligomerization Domain (OD), and the carboxy terminal regulatory domain (α). Then Δ40, Δ133, and Δ160 p53 isoforms are represented with the corresponding molecular weight on the right. Dotted lines illustrate epitope boundaries of p53 antibodies used for Western blot analysis (the text color is the same of the recognized region in the protein). Red arrows indicate the multiple epitopes recognized by SAPU polyclonal antibody. Created with BioRender.com.

**Supplementary Figure 2. Functional characterization of *TP53* K132T mutation from 92.1 cell line by yeast reporter assays.** **A)** Transactivation ability (percentage versus wild-type set as 100%) of *TP53* K132T mutation by using four different yeast reporter strains (yLFM-P21-5’, yLFM-BAX A+B, yLFM-PUMA, and yLFM-MDM2P2C) after 8 hours of growth in 0.128% Galactose at 30°C and 37°C. **B)** Dominant negative potential (percentage versus wild-type set as 100%) of *TP53* K132T mutation in yLFM-P21-5’ yeast reporter strain after 8 hours of growth in 0.016% Galactose.

**Supplementary Figure 3. Characterization of p53 targets transcriptional activation in UM cell lines by RT-qPCR.** **A)** RT-qPCR analysis of expression changes in p53 targets (p21, MDM2, PUMA, KILLER) in untreated and treated cells with 10 μM cisplatin or 20 Gy proton irradiation in 92.1, MEL270, and UPMD1 cells; bars represent the average and the standard deviations of at least 4 biological replicates. GAPDH and β-Actin were used as reference genes. **B)** Analysis as in A in MEL290, UPMM1, and UPMM2 cells; bars represent the average and the standard deviations of at least 3 biological replicates. **C)** Analysis as in A in UPMD2 and MEL285 cells; bars represent the average and the standard deviations of at least 3 biological replicates. **D)** Analysis as in A in OMM1 and OMM2.5 cells; bars represent the average and the standard deviations of at least 3 biological replicates. Whole panel: * = p<0.05; ** = p<0.01; *** = p<0.001.

**Supplementary Figure 4. Localization of p53 protein** **by cytoplasmic-nuclear fractionation in 92.1 cells untreated and treated with 10 μM cisplatin for 24 hours.** **A)** A representative Western blot of the analysis using p53-DO1 to detect full-length p53 and relative quantification (at least three independent biological replicates). The quantifications show the significance values of each treated condition relative to the untreated one using the multiple T-test (** = p<0.01; *** = p<0.001). **B)** A representative Western blot of the analysis using KJC12 to detect the Δ160p53α isoform. GAPDH, HSP70, and Histone H3 were used as loading controls for the cytoplasmic and the chromatin-bound protein fraction, respectively.

**Supplementary Figure 5.** **Determination of direct interaction of different p53 isoforms with full-length p53α.** Shown are western blots derived from Co-IP experiments on protein lysates from 92.1 cells treated with 10 μM cisplatin (**A**) or A549 clones over-expressing Δ40p53α (**B**), Δ133p53α (**C**), and Δ133p53β (**D**) isoforms. IPs were performed with BP53.10 (**A**) and DO-1 antibody (**B-D**) (to immunoprecipitate full-length p53α). Input (5%) was used as positive control and normal IgG antibody as negative control. Western blots were performed using 79.3 (β-specific) (**A**) and SAPU (pantropic) (**B-D**) primary antibodies to detect respectively the presence of full-length p53β (**A**) and full-length p53α, Δ40p53α, Δ133p53α, and Δ133p53β (**B-D**). HSP70 was used as loading control.

**Supplementary Figure 6.** **Characterization of p53 pathway functionality and p53 isoforms expression in UPMD2 and MEL285 primary UM cell lines. A)** Left panels: a representative Western blot of p53 and p53 targets (p21 and MDM2) expression in untreated (-) and treated (+) cells with 10 μM cisplatin. GAPDH expression was used as a reference protein. Right panel: relative quantification; bars represent the average and the standard deviations of at least 3 biological replicates. **B)** Left panels: a representative Western blot of p53 isoforms expression as in panel A and detected as described in Figure 2 (low exposure). p53 isoforms were detected using KJC12 pantropic antibody. Right panels: Western blot as in B (high exposure) with relative quantification; bars represent the average and the standard deviations of at least 3 biological replicates. Whole panel: * = p<0.05.

**Supplementary Figure 7.** **Characterization of p53 pathway functionality and p53 isoforms expression in OMM1 and OMM2.5 metastatic UM cell lines. A)** Left panels: a representative Western blot of p53 and p53 targets (p21 and MDM2) expression in untreated (-) and treated (+) cells with 10 μM cisplatin or 20 Gy proton irradiation. GAPDH was used as a reference protein. Right panel: relative quantification; bars represent the average and the standard deviations of at least 3 biological replicates. **B-C)** A representative Western blot of p53 isoforms expression as in panel A and detected as described in Figure 2 with SAPU (**B**) and KJC12 (**C**) p53 pantropic antibodies (upper panels low exposure; bottom panels: high exposure). Shown results were obtained staining firstly with SAPU antibody and subsequently by re-blotting the same membrane with KJC12 antibody after stripping. **D)** Quantification of the analysis from B and C; bars represent the average and the standard deviations of at least 3 biological replicates. Whole panel: * = p<0.05; ** = p<0.01.

**Supplementary Figure 8. Confirmation of the identified p53 isoforms. A-D)** Representative Western blots of different version of p53β isoforms expression in untreated (-) and treated (+) UM cells with 10 μM cisplatin detected with β-sheep antibody (β-specific). Vinculin was used as a reference protein. **E-F)** Representative Western blots of different version of p53α isoforms expression in untreated (-) and treated (+) UM cells with 10 μM cisplatin detected with BP53.10 antibody (α-specific). Vinculin was used as a reference protein.

**Supplementary Figure 9.** **Evaluation of earlier time points’ effect on p53 pathway functionality in 92.1, UPMM1, MEL285, and OMM2.5 UM cell lines by Western blot. A)** A representative Western blot of p53 and p21 expression in untreated (-) and treated cells with 10 μM cisplatin for 8 and 24 hours. GAPDH was used as a reference protein. **B)** Quantification of the analysis from A; bars represent the average and the standard deviations of at least 3 biological replicates. **** = p<0.0001.

**Supplementary Figure 10.** **Characterization of p53 isoforms expression at mRNA level in 92.1, MEL270, OMM1, and OMM2.5 UM cell lines. A)** Analysis of expression changes of short (Δ133/160p53α/β/γ) and long (Δ 40p53α/β/γ and p53α/β/γ) p53 isoforms in untreated 92.1, MEL270, OMM1, and OMM2.5 cells by two-step nested qPCR. A region between exons 5 and 8 of the *TP53* gene shared by all the p53 isoforms was used as a reference. **B)** Comparison of the results from panel A for the evaluation of p53 isoforms expression changes in MEL270 primary cell line with respect to OMM2.5 metastatic cell line, both deriving from the same patient. Whole panel: * = p<0.05; ** = p<0.01; *** = p<0.001.

**Supplementary Figure 11. p53 genomics landscape from publicly available omic data. A)** *TP53* lollipop plot presenting variants in UM samples of the dataset from Newell and collaborators. *TP53* variants are reported as dots, amino acids substitutions are colored in green. Protein domain and exon structure are reported (green P53_TAD: p53 transactivation domain; purple P53_tetramer: p53 tetramerization domain; the central DNA binding domain is indicated as a red P53 domain. **B)** *TP53* CNAs profile in the TCGA UM dataset. CNA events on chromosome 17: losses are represented in blue, gains in red. **C)** *TP53* expression from the TCGA UM dataset. High (chr3 monosomy) and low chr3 disomy) risk patients are reported in red and blue, respectively. The upper panel reports the distributions of patients that developed metastasis during follow-up, while the remaining are reported in the bottom panel. *TP53* expression differences are not significant in either of the two groups (Welch two sample t-test, p-value = 0.97). Data have been extracted from the Combat gene expression file published by Piaggio and colleagues ^3^. **D)** *TP53* methylation score reported as β-value * 100 as in panel C. The difference in methylation levels between high risk and low risk samples is significant (Welch two sample t-test, p-value < 10^-7^) in patients that develop metastasis.
