# Supplementary figures and images for: Exploring p53 isoforms: unraveling heterogeneous p53 tumor suppressor functionality in uveal melanoma
Source: Cell Death Discov. 2025 Dec 5;12:39. doi: 10.1038/s41420-025-02891-1 (PMC12827457; doi:10.1038/s41420-025-02891-1)

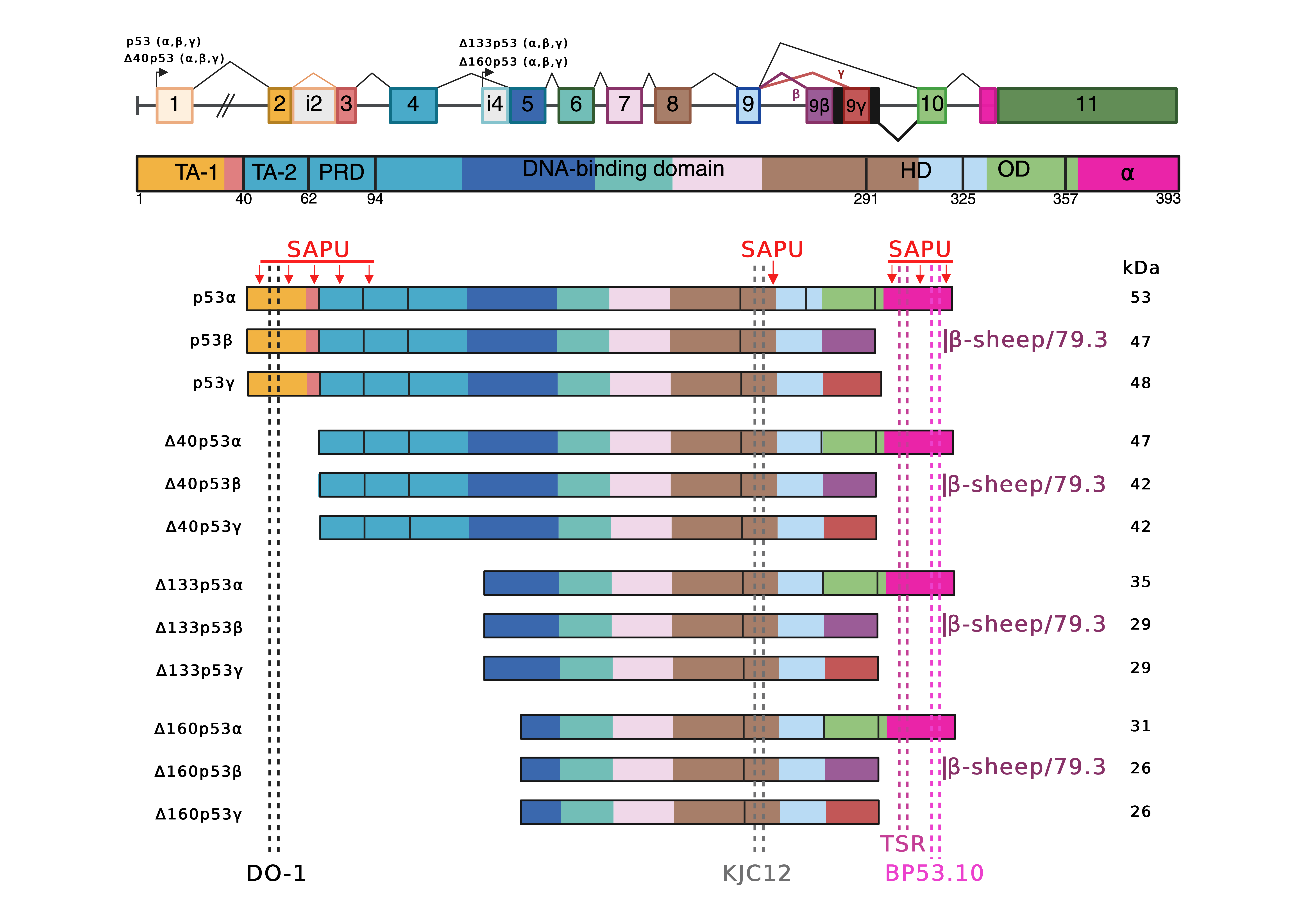

Supplement: Supplementary file 2 — Supplementary Figure 1 [file 41420_2025_2891_MOESM2_ESM.png]

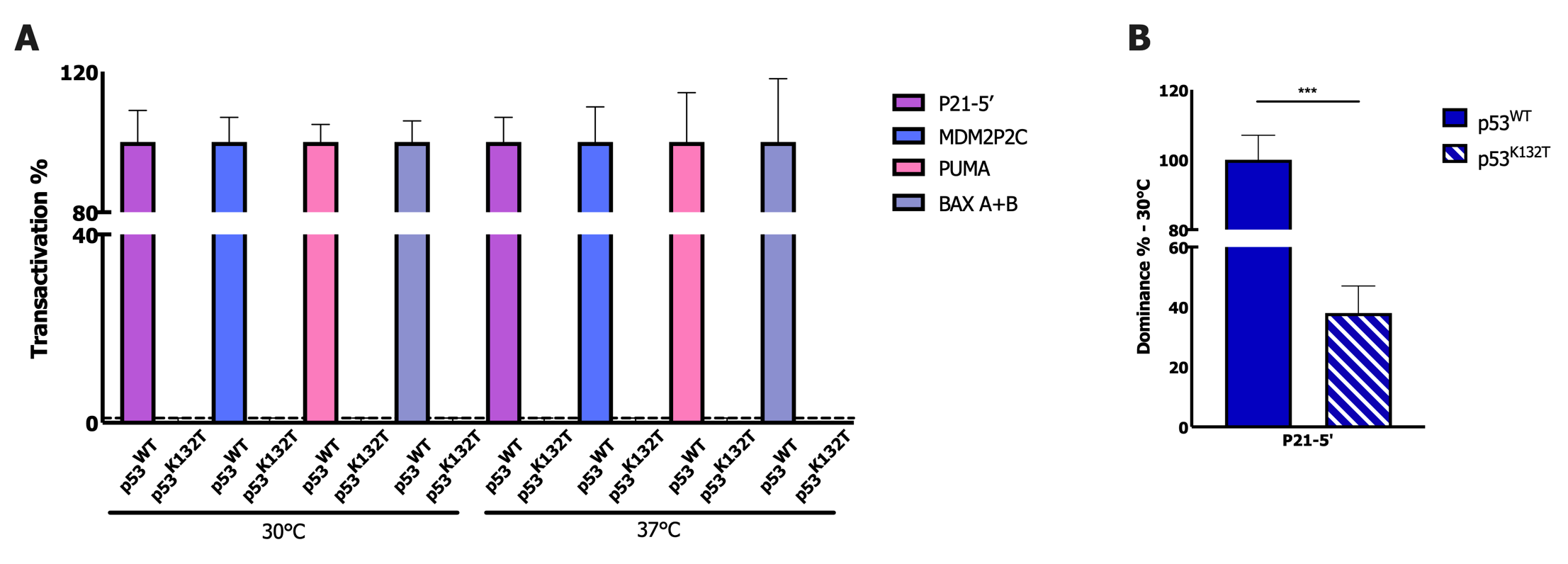

Supplement: Supplementary file 3 — Supplementary Figure 2 [file 41420_2025_2891_MOESM3_ESM.tif]

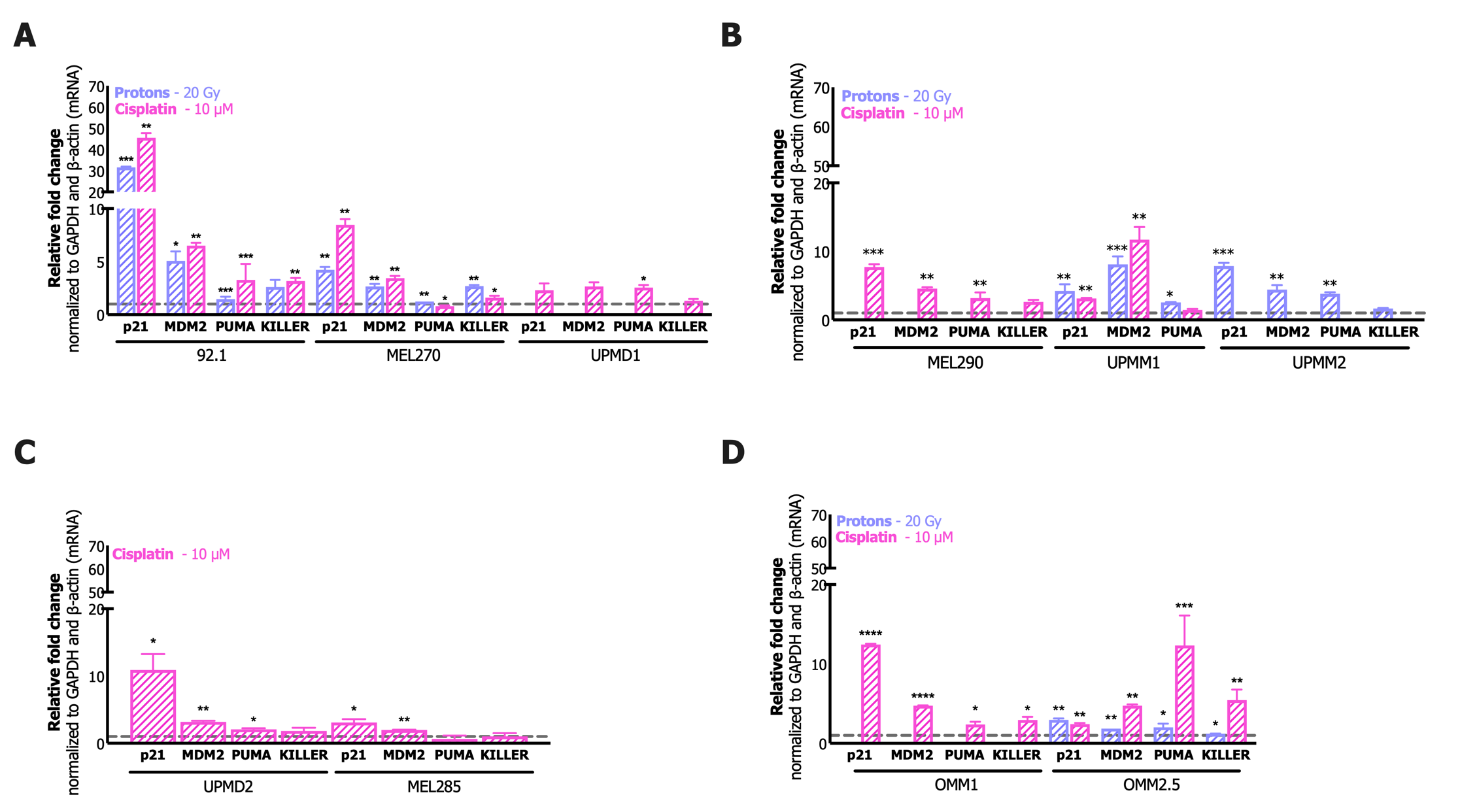

Supplement: Supplementary file 4 — Supplementary Figure 3 [file 41420_2025_2891_MOESM4_ESM.tif]

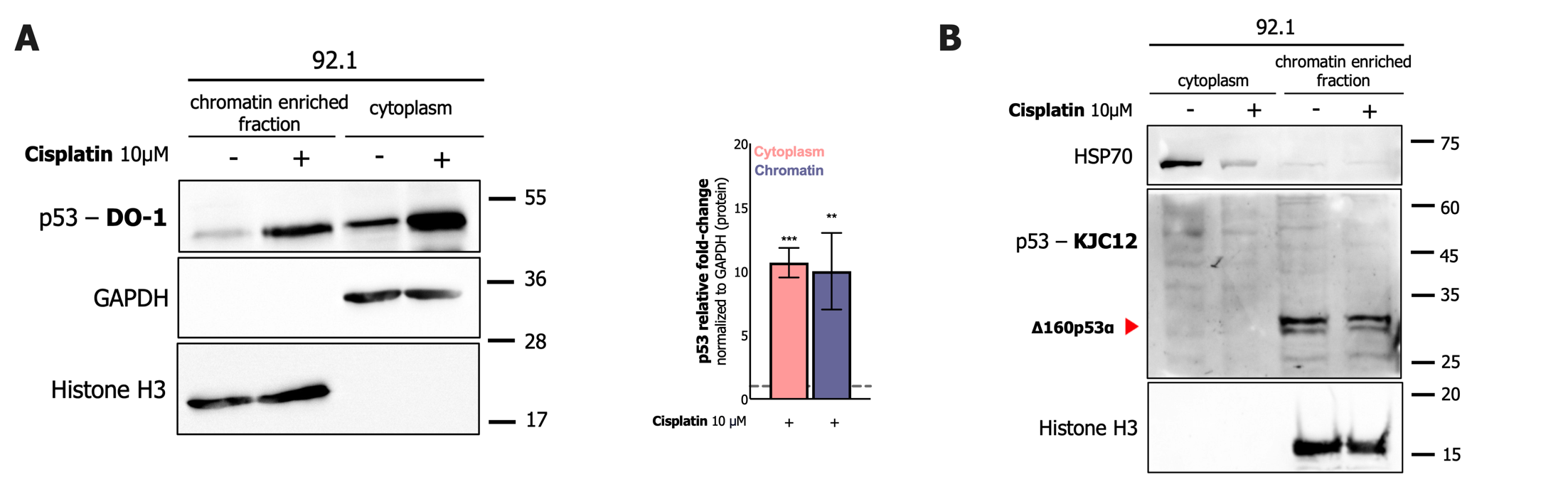

Supplement: Supplementary file 5 — Supplementary Figure 4 [file 41420_2025_2891_MOESM5_ESM.tif]

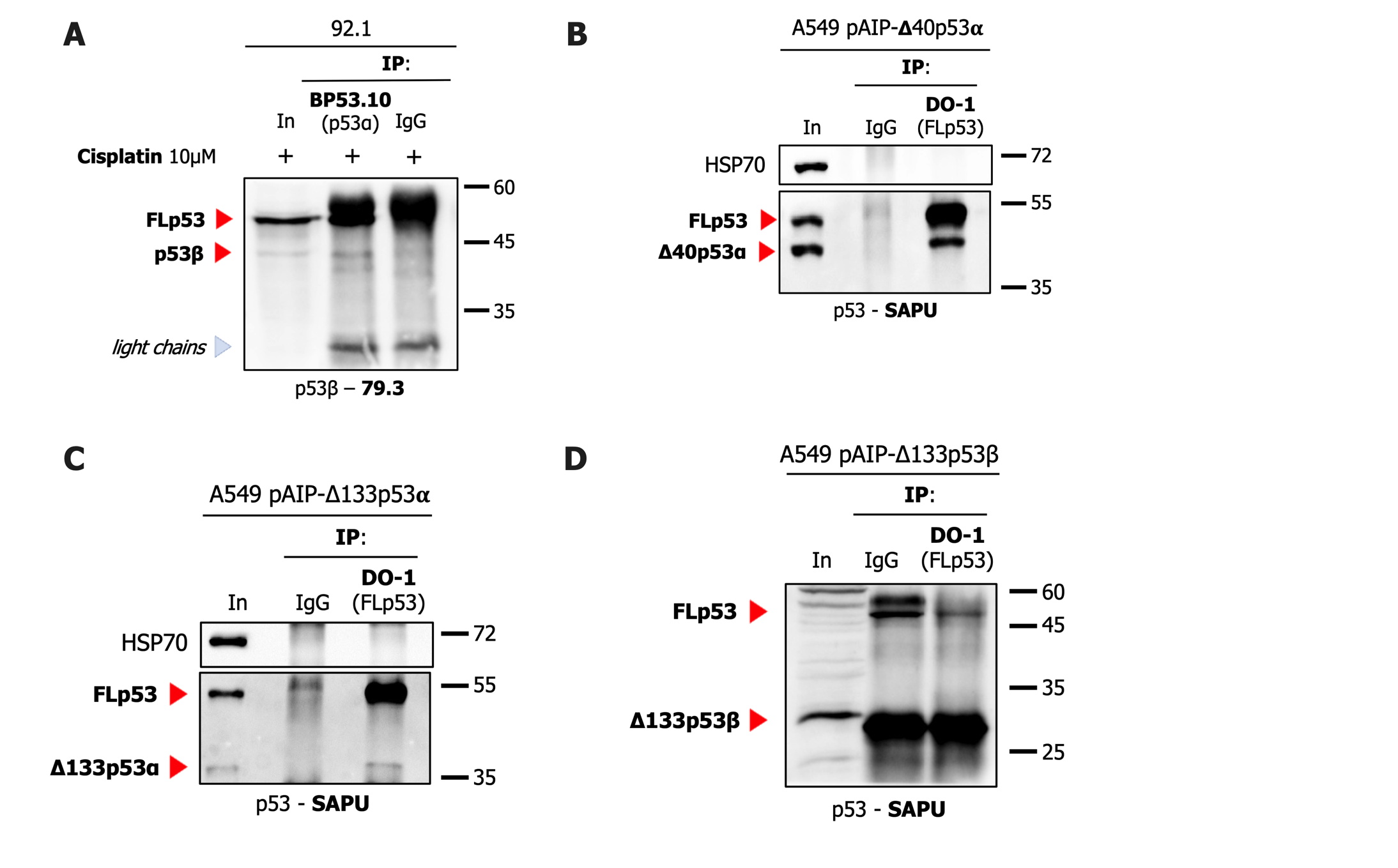

Supplement: Supplementary file 6 — Supplementary Figure 5 [file 41420_2025_2891_MOESM6_ESM.tif]

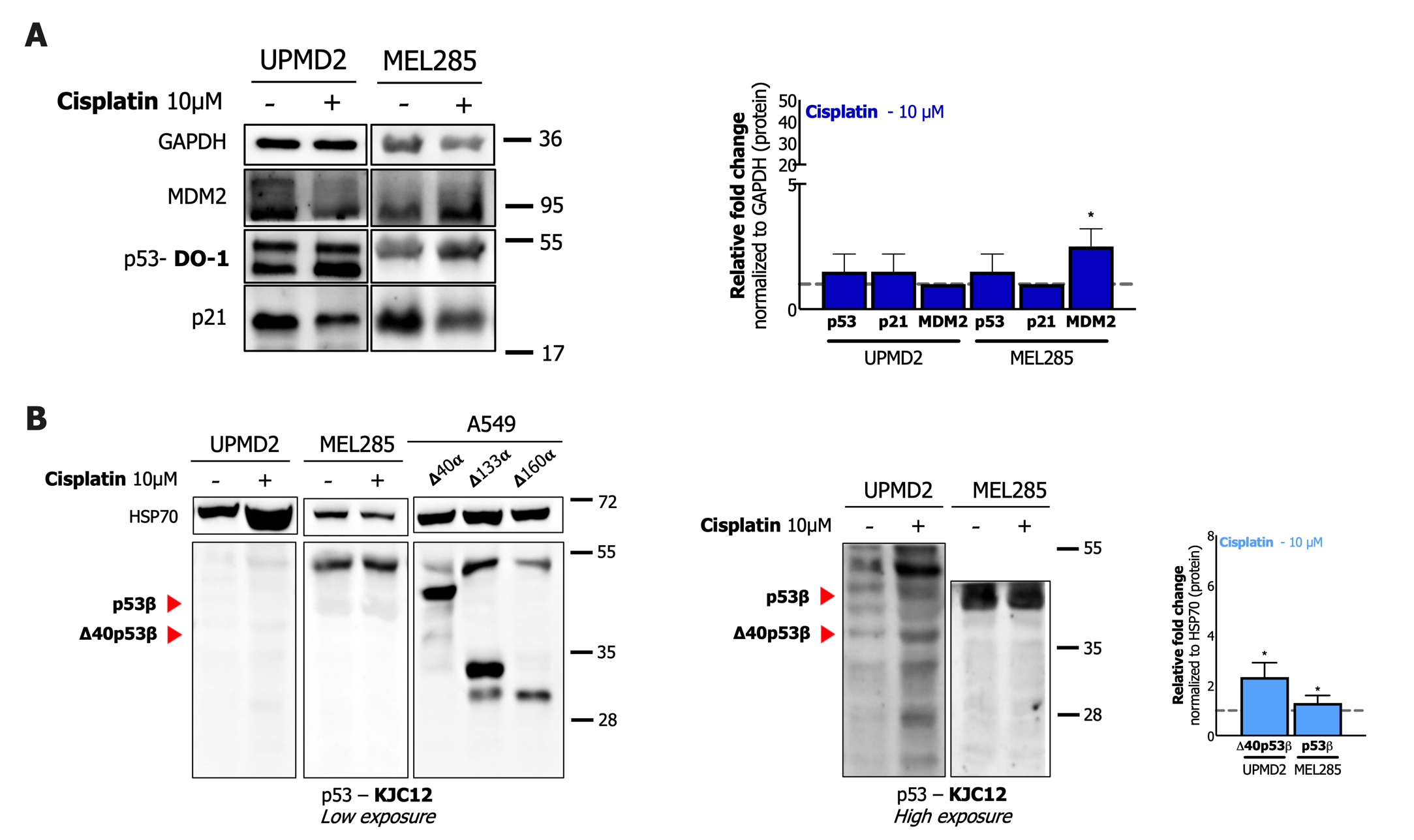

Supplement: Supplementary file 7 — Supplementary Figure 6 [file 41420_2025_2891_MOESM7_ESM.tif]

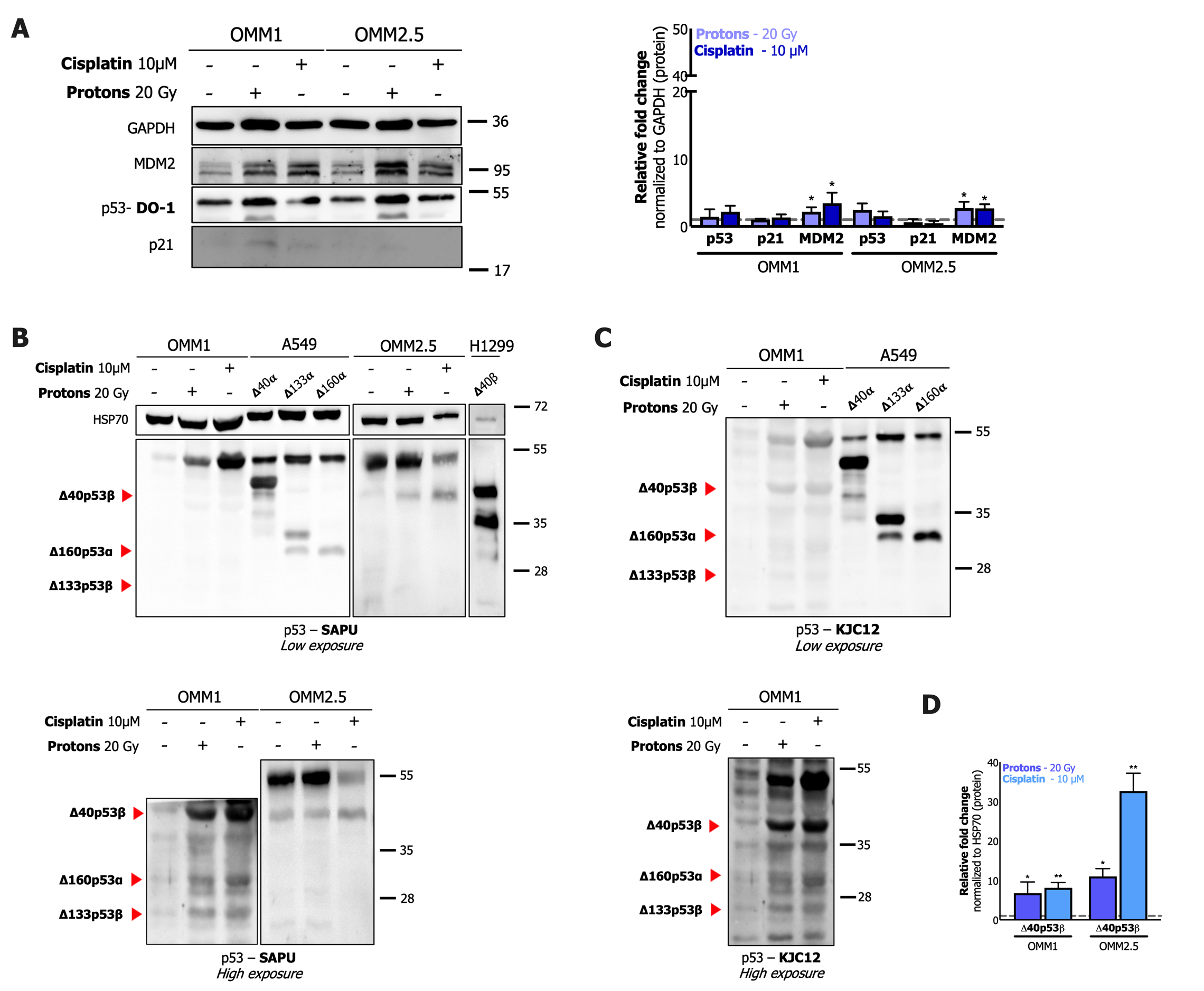

Supplement: Supplementary file 8 — Supplementary Figure 7 [file 41420_2025_2891_MOESM8_ESM.tif]

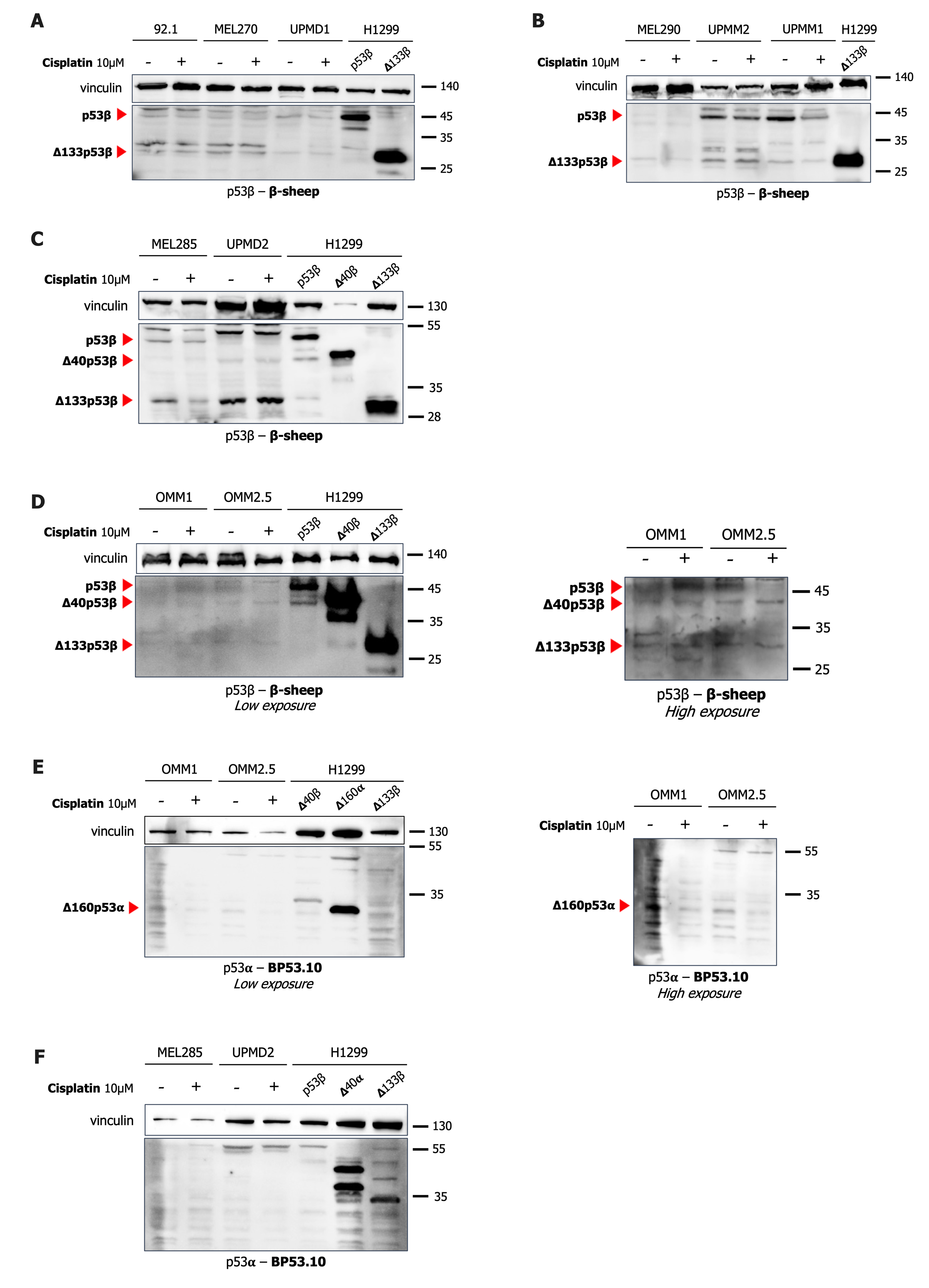

Supplement: Supplementary file 9 — Supplementary Figure 8 [file 41420_2025_2891_MOESM9_ESM.tif]

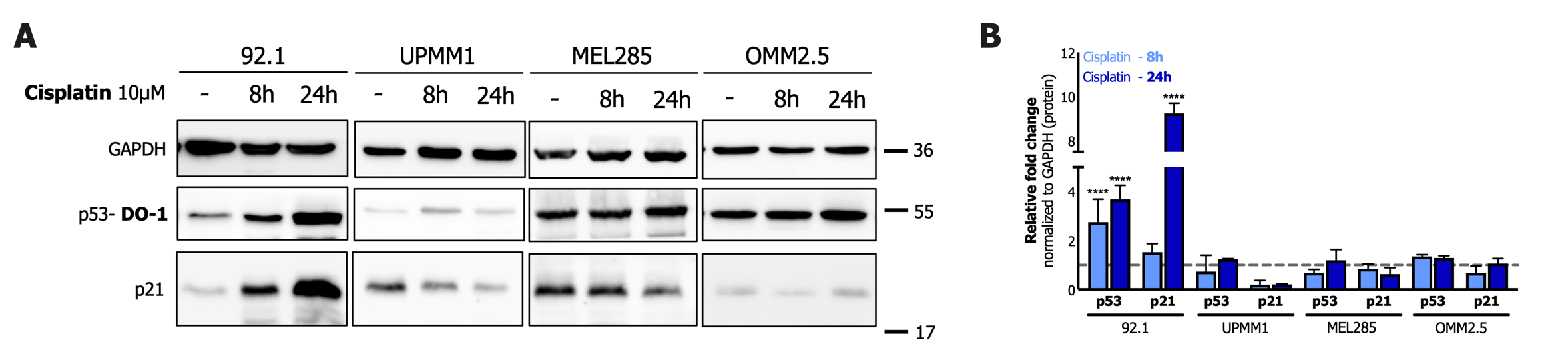

Supplement: Supplementary file 10 — Supplementary Figure 9 [file 41420_2025_2891_MOESM10_ESM.tif]

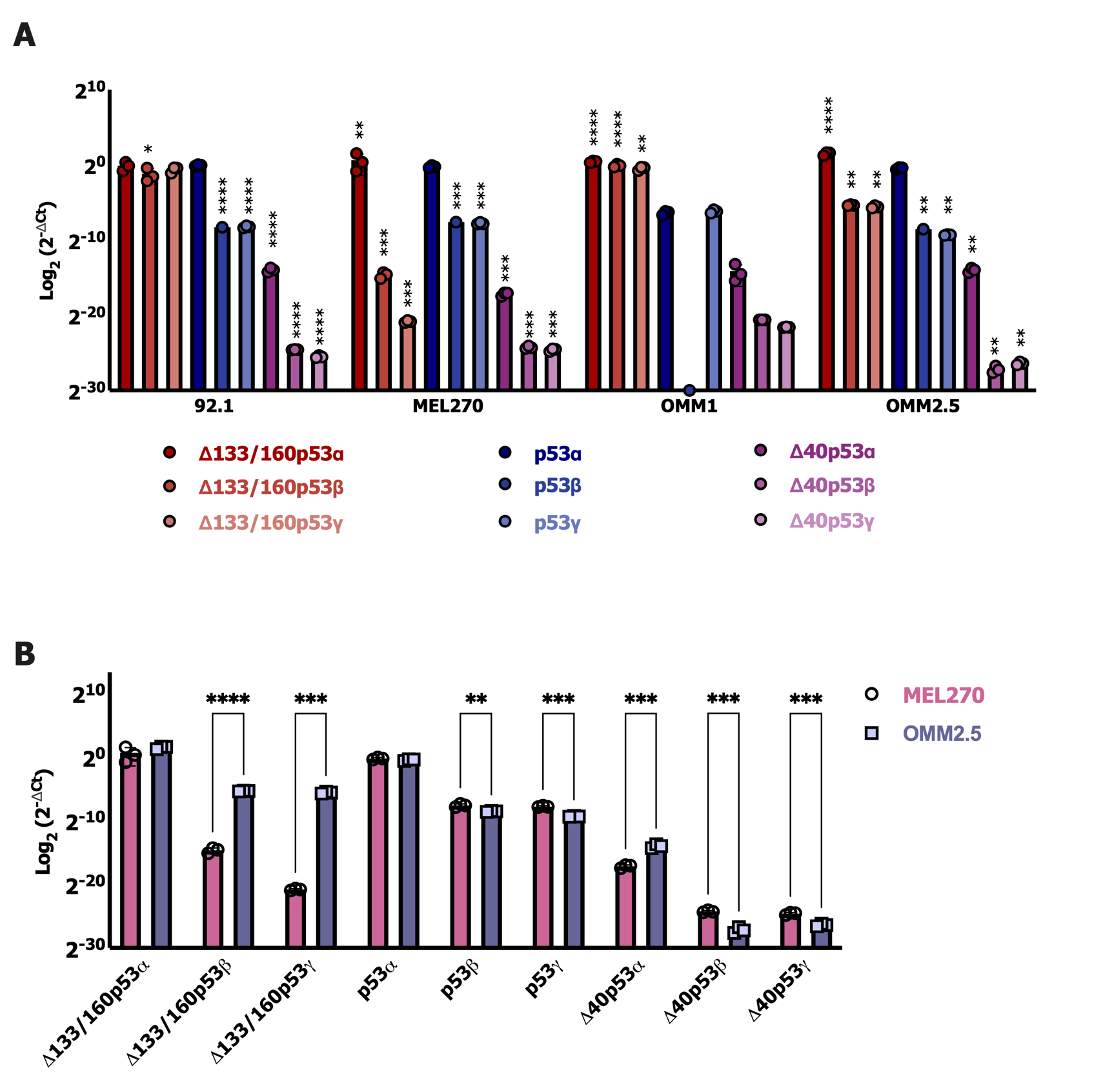

Supplement: Supplementary file 11 — Supplementary Figure 10 [file 41420_2025_2891_MOESM11_ESM.tif]

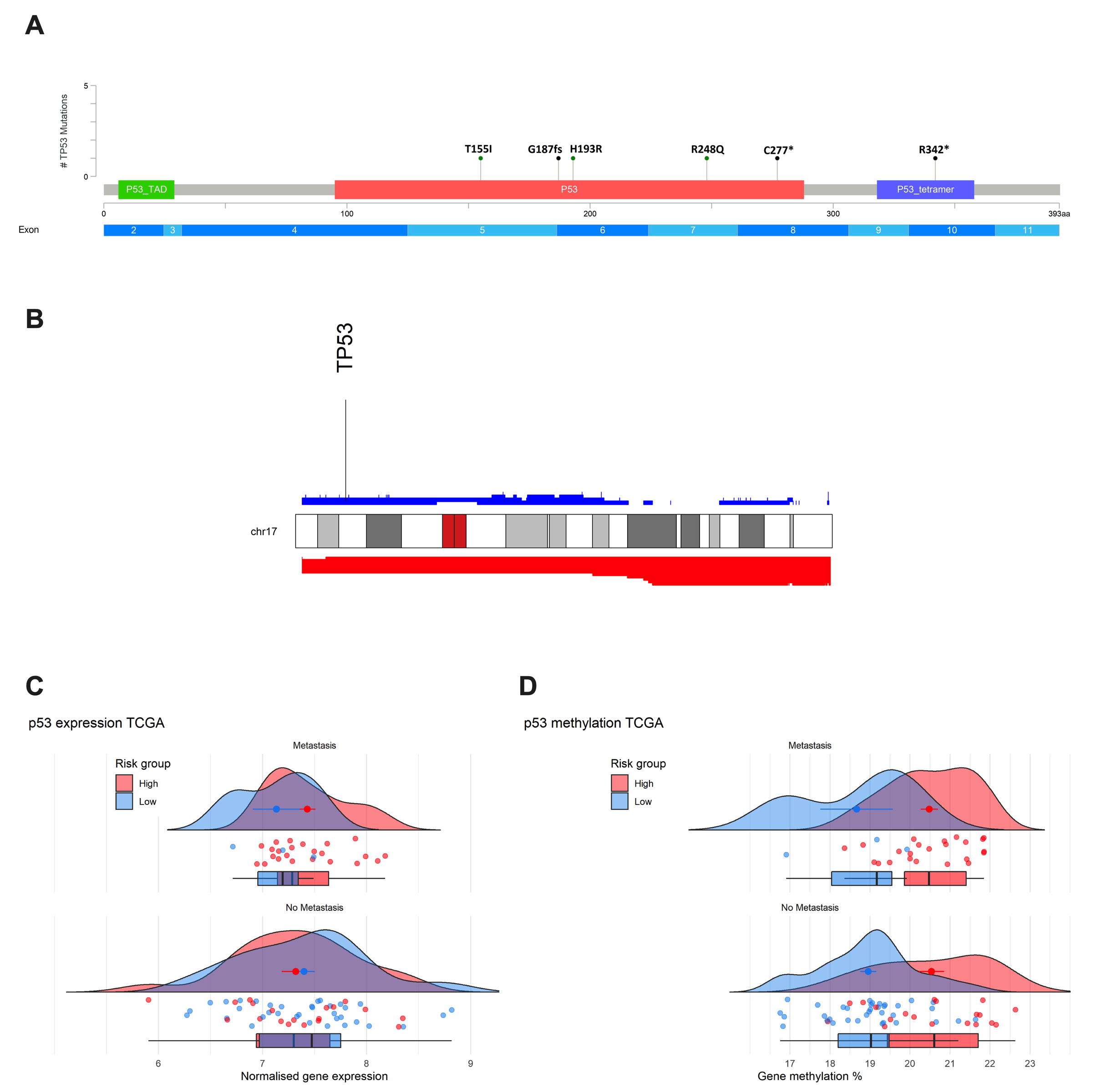

Supplement: Supplementary file 12 — Supplementary Figure 11 [file 41420_2025_2891_MOESM12_ESM.tif]
