# Supplementary material for: Exploring p53 isoforms: unraveling heterogeneous p53 tumor suppressor functionality in uveal melanoma
Source: Cell Death Discov. 2025 Dec 5;12:39. doi: 10.1038/s41420-025-02891-1 (PMC12827457; doi:10.1038/s41420-025-02891-1)
Supplement: Supplementary file 13 — Supplementary Table 1 [file 41420_2025_2891_MOESM13_ESM.docx]

| **Cell line** | **Accession number^§^** | **Stage** | ***BAP1*** | ***GNAQ*** | ***GNA11*** | **Chromosome 3** |
| --- | --- | --- | --- | --- | --- | --- |
| UPMM1 | CVCL_C299 | primary | wt | R183Q | wt | Monosomy |
| UPMM2 | CVCL_C294 | primary | I586H fs * 57 | Q209L | wt | Isodisomy |
| 92.1 | CVCL_C8607 | primary | wt | Q209L | wt | Disomy |
| MEL270 | CVCL_C302 | primary | wt | Q209P | wt | Disomy, loss 3p24, loss 3q21.2-3q24 |
| MEL285 | CVCL_C303 | primary | wt | wt | wt | Disomy, loss 3p26-pter |
| MEL290 | CVCL_C304 | primary | wt | wt | wt | Disomy, loss 3p26-pter |
| UPMD1 | CVCL_C297 | primary | wt | wt | Q209L | Disomy |
| UPMD2 | CVCL_C298 | primary | wt | wt | Q209L | Isodisomy |
| OMM1 | CVCL_6939 | metastatic (hypodermis) | wt | wt | Q209L | Net gain 3q |
| OMM2.5 | CVCL_C307 | metastatic (liver) | wt | Q209P | wt | Disomy |

**Supplementary Table 1.** List of the UM cell lines used in present study. The specific features of the UM cell lines were retrieved from previously published studies [1-4]. wt = wild-type; ^§^ = Cellosaurus accession number (https://www.cellosaurus.org). All UM cell lines express a wild type *BAP1* coding sequence with the exception of UPMM2 [5]. fs * 57 = frameshift mutation generating a Histidine at codon 586 and a termination codon after 57 novel amino acids downstream.

**REFERENCES**

[1] Nareyeck G, Zeschnigk M, Prescher G, Lohmann DR, Anastassiou G. Establishment and characterization of two uveal melanoma cell lines derived from tumors with loss of one chromosome 3. Exp Eye Res. 2006;83:858-64.

[2] Nareyeck G, Zeschnigk M, Bornfeld N, Anastassiou G. Novel cell lines derived by long-term culture of primary uveal melanomas. Ophthalmologica. 2009;223:196-201.

[3] Griewank KG, Yu X, Khalili J, Sozen MM, Stempke-Hale K, Bernatchez C, et al. Genetic and molecular characterization of uveal melanoma cell lines. Pigment cell & melanoma research. 2012;25:182-7.

[4] Jager MJ, Magner JA, Ksander BR, Dubovy SR. Uveal Melanoma Cell Lines: Where do they come from? (An American Ophthalmological Society Thesis). Trans Am Ophthalmol Soc. 2016;114:T5.

[5] Amaro AA, Gangemi R, Emionite L, Castagnola P, Filaci G, Jager MJ, Tanda ET, Spagnolo F, Mascherini M, Pfeffer U, Croce M. Cerivastatin Synergizes with Trametinib and Enhances Its Efficacy in the Therapy of Uveal Melanoma. Cancers (Basel). 2023 Jan 31;15(3):886. doi: 10.3390/cancers15030886. PMID: 36765842; PMCID: PMC9913575.
