# Supplementary material for: Exploring p53 isoforms: unraveling heterogeneous p53 tumor suppressor functionality in uveal melanoma
Source: Cell Death Discov. 2025 Dec 5;12:39. doi: 10.1038/s41420-025-02891-1 (PMC12827457; doi:10.1038/s41420-025-02891-1)
Supplement: Supplementary file 15 — Supplementary Table 3 [file 41420_2025_2891_MOESM15_ESM.docx]

| **Gene** | **Forward primer (Fw)** | **Reverse primer (Rv)** |
| --- | --- | --- |
| GAPDH | 5’-TCCAAAATCAAGTGGGGCGA-3’ | 5’-AGTAGAGGCAGGGATGATGT-3’ |
| ACTB | 5’-CTGGAACGGTGAAGGTGACA-3’ | 5’-AAGGGACTTCCTGTAACAAT-3’ |
| p21 | 5’-CTGGAGACTCTCAGGGTCGAA-3’ | 5’-GATTAGGGCTTCCTCTTGGAG-3’ |
| MDM2 | 5’-GGCCTGCTTTACATGTGCAA-3’ | 5’-GCACAATCATTTGAATTGGTTGTC-3’ |
| KILLER | 5’-TGACTCATCTCAGAAATGTCAATTCTTA-3’ | 5’-GGACACAAGAAGAAAACCTTAATGC-3’ |
| PUMA | 5’-CCTGGAGGGTCCTGTACAATCT-3’ | 5’-GCACCTAATTGGGCTCCATCT-3’ |
| p53 short (pre-amp) | 5’-TTCACTTGTGCCCTGACTTTCAACT-3’ | 5’-CTTCCCAGCCTGGGCATCCTTG-3’ |
| p53 long (pre-amp) | 5’-GTCACTGCCATGGAGGAGCCGCA-3’ | 5’-CTTCCCAGCCTGGGCATCCTTG-3’ |
| FLp53 | 5’-AGACCTATGGAAACTACTTCCT-3’ | 5’-CTCACGCCCACGGATCTGA-3’ (α)  5’-AAGCTGGTCTGGTCCTGAAAGGGT-3’ (β)  5’-TCGTAAGTCAAGTAGCATCTGAAGG-3’ (γ) |
| Δ40p53 | 5’-GATCCATTGGAAGGGCAGGCC-3’ | 5’-CTCACGCCCACGGATCTGA-3’ (α)  5’-AAGCTGGTCTGGTCCTGAAAGGGT-3’ (β)  5’-TCGTAAGTCAAGTAGCATCTGAAGG-3’ (γ) |
| Δ133/Δ160p53 | 5’-ACTCTGTCTCCTTCCTCTTCCTACAG-3’ | 5’-CTCACGCCCACGGATCTGA-3’ (α)  5’-AAGCTGGTCTGGTCCTGAAAGGGT-3’ (β)  5’-TCGTAAGTCAAGTAGCATCTGAAGG-3’ (γ) |
| p53 all isoforms | 5’-CCATCTACAAGCAGTCACAGCA-3’ | 5’-TTCTTGCGGAGATTCTCTTCCT-3’ |

**Supplementary Table 3.** Sequences of primers used for qPCR analysis in present study.
