# Supplementary material for: Exploring p53 isoforms: unraveling heterogeneous p53 tumor suppressor functionality in uveal melanoma
Source: Cell Death Discov. 2025 Dec 5;12:39. doi: 10.1038/s41420-025-02891-1 (PMC12827457; doi:10.1038/s41420-025-02891-1)
Supplement: Supplementary file 16 — Supplementary Table 4 [file 41420_2025_2891_MOESM16_ESM.docx]

| **UM Cell Lines** | **FASAY** | ***TP53*** | ***rs1042522*** |
| --- | --- | --- | --- |
| UPMM1 | 12.85% | WT | P72R |
| UPMM2 | 8.72% | WT | P72P/P72R |
| 92.1 | 37.5% | **MUT**: **K132T** | P72R |
| MEL270 | 8.76% | WT | P72P/P72R |
| MEL285 | 16.70% | WT | P72P/P72R |
| MEL290 | 16.88% | WT | P72R |
| UPMD1 | 11.44% | WT (R213R) | P72P/P72R |
| UPMD2 | 9.06% | WT | P72P/P72R |
| OMM1 | 7.36% | WT | P72R |
| OMM2.5 | 8.38% | WT | P72P/P72R |

**Supplementary Table 4.** Results of FASAY assay and *TP53* coding sequence Sanger sequencing (codons 42-375) on UM cell lines. WT = wild-type; MUT = mutant. P72P (CCC>CCC); P72R (CCC> CGC); K132T (AAG>ACG); R213R (CGA>CGG).
