# Supplementary material for: Exploring p53 isoforms: unraveling heterogeneous p53 tumor suppressor functionality in uveal melanoma
Source: Cell Death Discov. 2025 Dec 5;12:39. doi: 10.1038/s41420-025-02891-1 (PMC12827457; doi:10.1038/s41420-025-02891-1)
Supplement: Supplementary file 17 — Supplementary Table 5 [file 41420_2025_2891_MOESM17_ESM.docx]

| **UM samples** | **FASAY** | ***TP53*** | ***rs1042522*** | **Metastases** | **CNV_3** |
| --- | --- | --- | --- | --- | --- |
| MU003 | 5.78% | WT | P72P/P72R | yes | losses |
| MU005 | 0.18% | WT | P72R | no | 2 |
| MU007 | 8.66% | WT | P72R | yes | 1 |
| MU008 | 16.09% | WT | P72P/P72R | yes | 2 |
| MU010 | 7.63% | WT | P72R | yes | 1 |
| MU012 | 6.12% | WT | P72P/P72R | yes | 1 |
| MU013 | 7.89% | WT | P72R | no | 2 |
| MU015 | 5.06% | WT | P72R | yes | losses |
| MU016 | 11.01% | WT | P72P/P72R | no | 2 |
| MU018 | 6.71% | WT | P72R | no | 1 |
| MU020 | 10.63% | WT | P72P | no | 2 |
| MU023 | 16.97% | WT | P72R | no | 2 |
| MU025 | 7.91% | WT | P72P/P72R | no | 2 |
| MU031 | 11.04% | WT | P72R | yes | 1 |
| MU032 | 7.24% | WT | P72R | no | 2 |
| MU033 | 2.52% | WT | P72P/P72R | no | n.a. |
| MU034 | 4.40% | WT | P72R | yes | 1 |
| MU036 | 7.36% | WT | P72R | no | 1 |
| MU037 | 7.97% | WT | P72R | no | 2 |
| MU039 | 1.55% | WT | P72R | no | 1 |
| MU040 | 4.33% | WT | P72R | yes | losses |
| MU041 | 5.76% | WT | P72P/P72R | no | 1 |
| MU042 | 13.24% | WT | P72R | yes | 1 |
| MU046 | 5.81% | WT | P72P/P72R | yes | 1 |
| MU050 | 8.05% | WT | P72P/P72R | yes | n.a. |
| MU060 | 12.40% | WT | P72R | yes | 2 |
| MU062 | 7.39% | WT | P72P/P72R | no | 2 |
| MU063 | 7.92% | WT | P72R | yes | 1 |
| MU064 | 9.01% | WT | P72P/P72R | yes | 1 |
| MU066 | 9.92% | WT | P72R | yes | 1 |
| MU075 | 11.53% | WT | P72P | yes | 1 |
| MU076 | 14.49% | WT | P72R | no | 2 |

**Supplementary Table 5**. Results of FASAY assay and *TP53* coding sequence Sanger sequencing (codons 42-375) on UM samples. P72P (CCC>CCC); P72R (CCC>CGC). CNV 3 = Copy Number Variations at chromosome 3. n.a. = chromosome 3 status not available. Losses = partial loss of chromosome 3 in different locations including part of *BAP1* gene; therefore, it can be considered functionally monosomic.
