# Supplementary material for: Exploring p53 isoforms: unraveling heterogeneous p53 tumor suppressor functionality in uveal melanoma
Source: Cell Death Discov. 2025 Dec 5;12:39. doi: 10.1038/s41420-025-02891-1 (PMC12827457; doi:10.1038/s41420-025-02891-1)

Figure 1

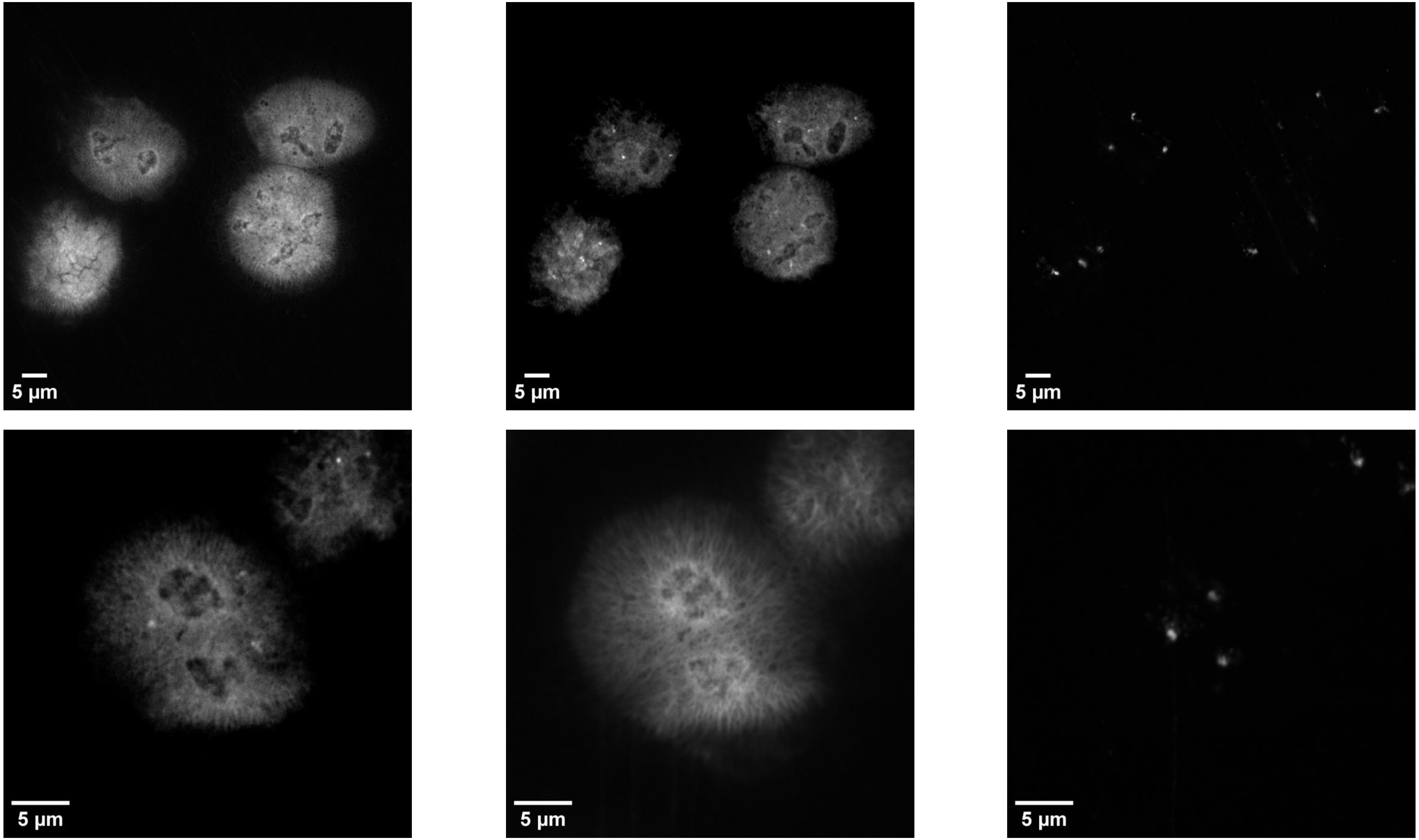

Figure 2A

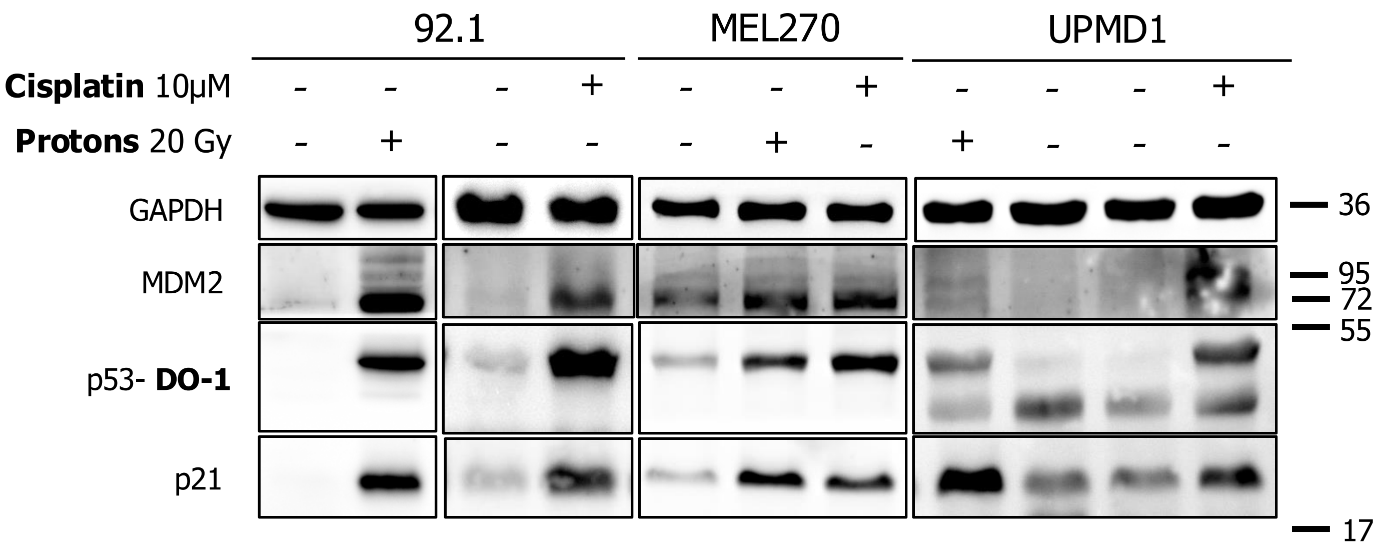
92.1
MEL270
UPMD1
Cisplatin 10μM
Protons 20 Gy
GAPDH
MDM2
p53- DO-1
p21
36
95
72
55
17

92.1

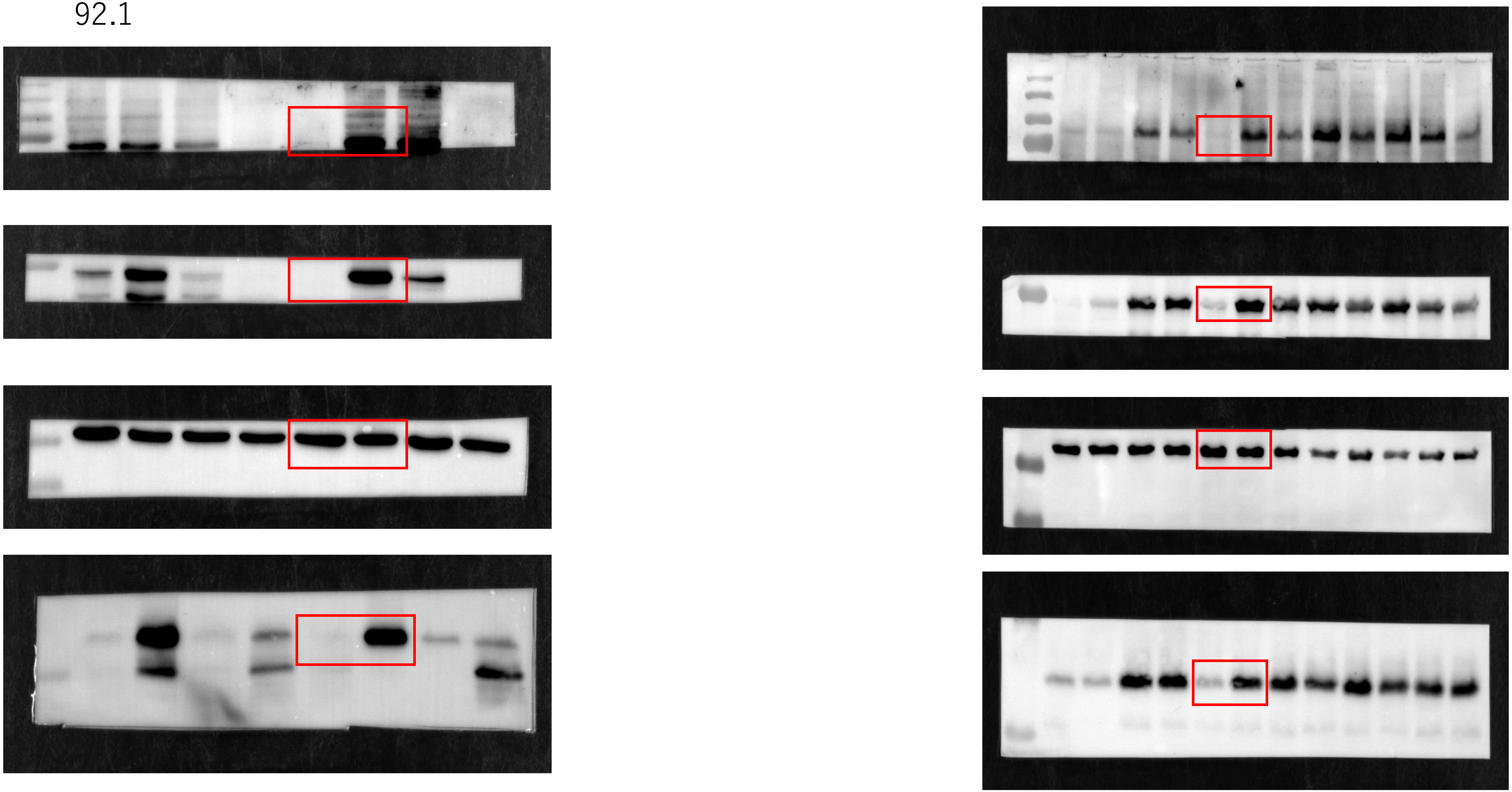

MEL270

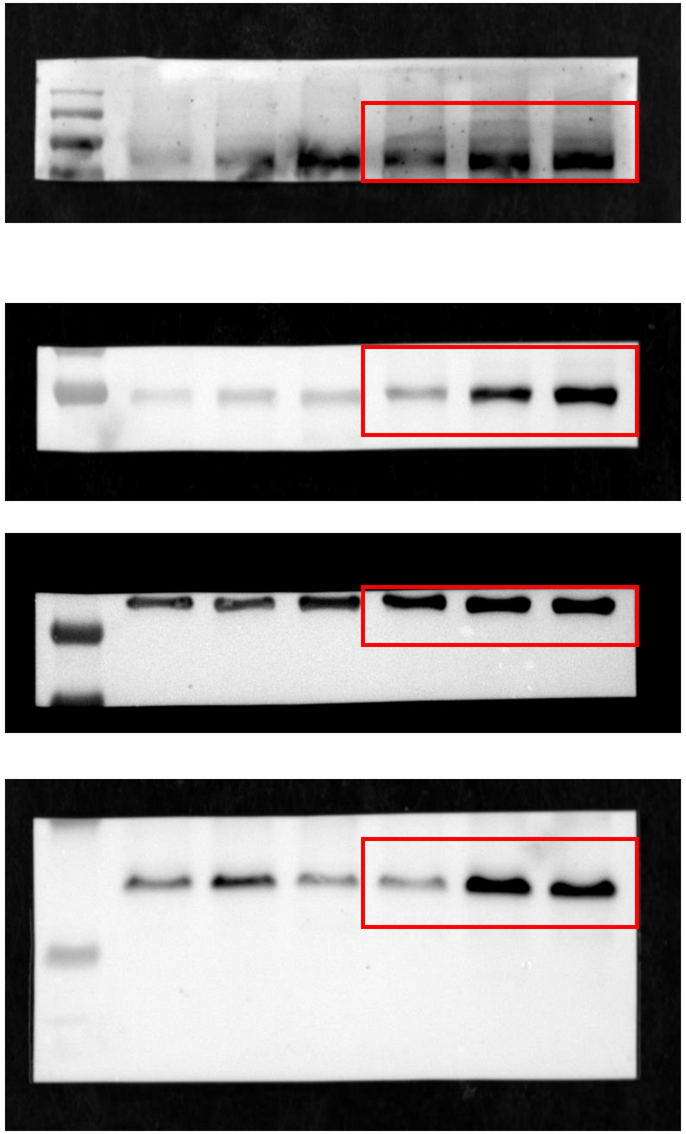

UPMD1

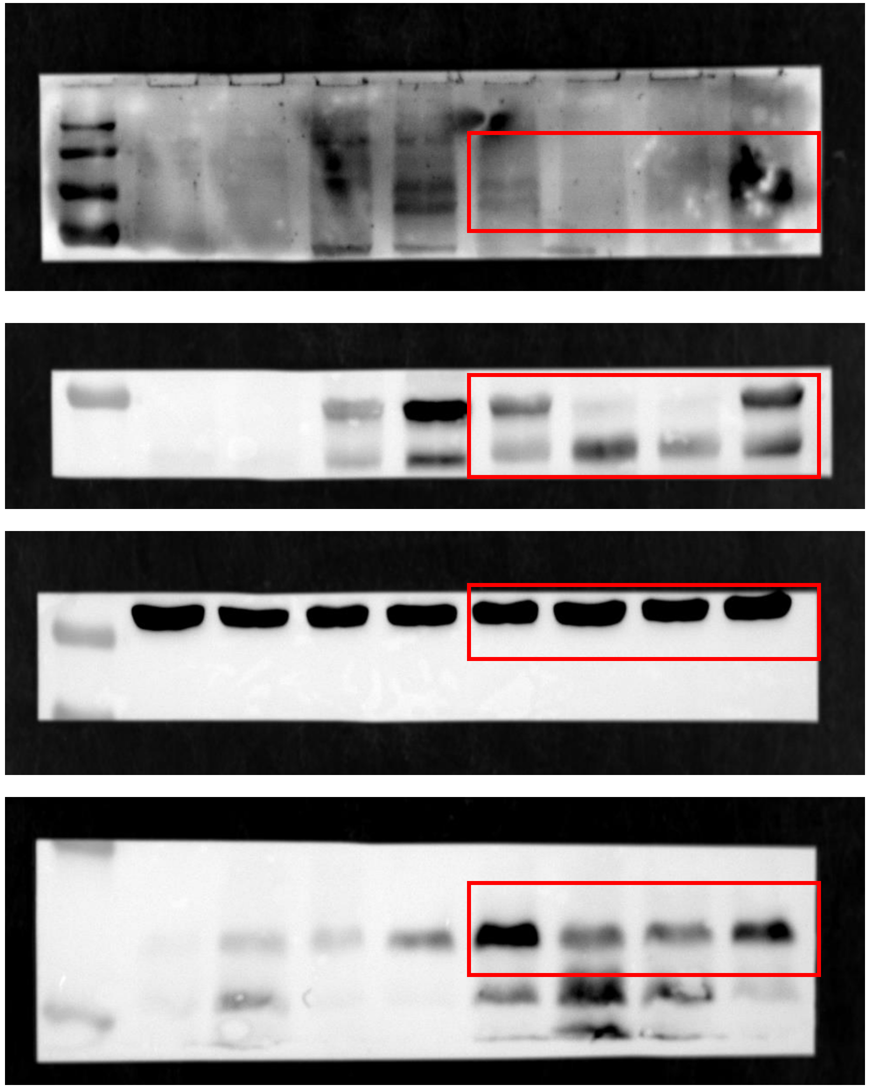

Figure 2B

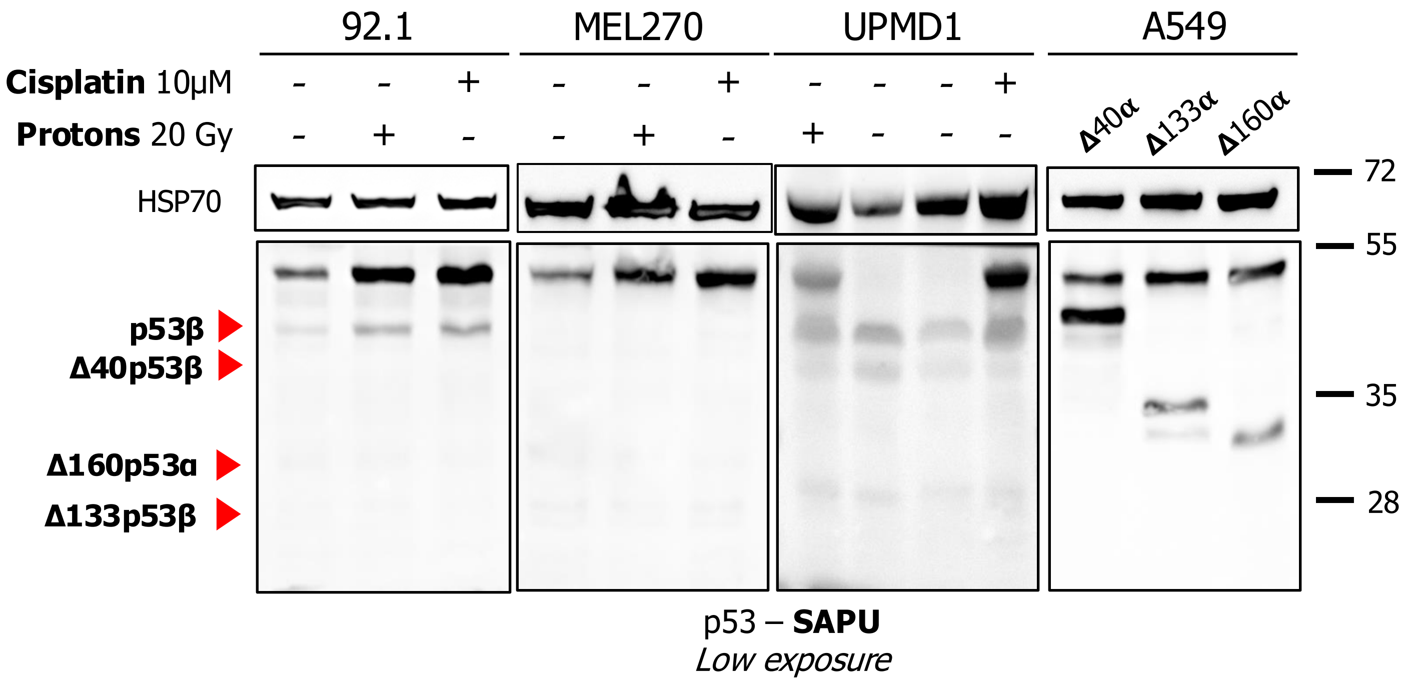
92.1
MEL270
UPMD1
A549
Cisplatin 10µM
Protons 20 Gy
Δ40α
Δ133α
Δ160α
HSP70
72
55
35
28
p53β
Δ40p53β
Δ160p53α
Δ133p53β
p53 – SAPU
Low exposure

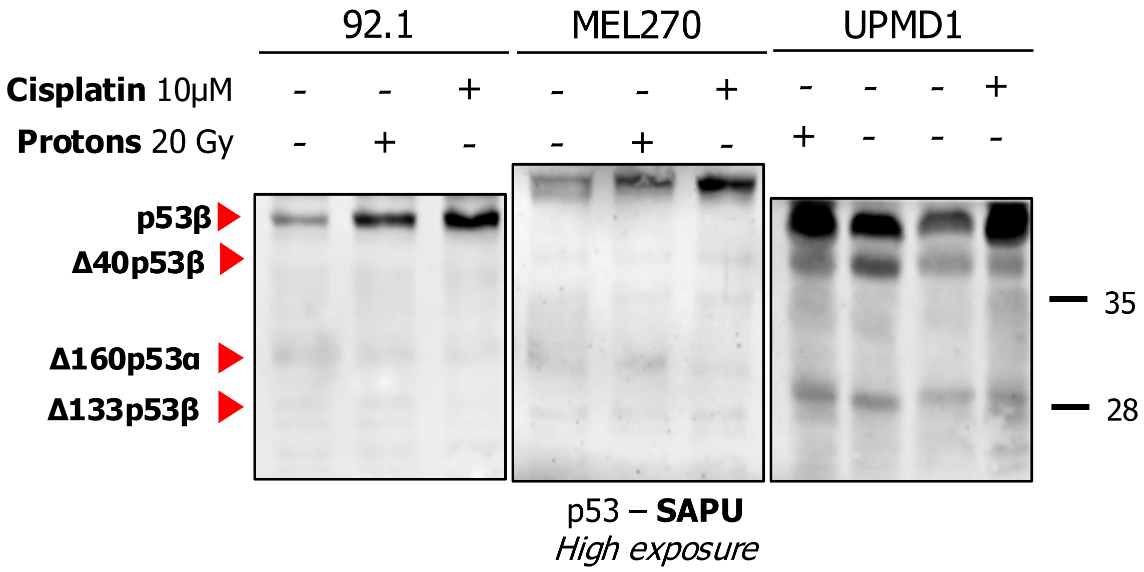
92.1
MEL270
UPMD1
Cisplatin 10µM
Protons 20 Gy
p53β
Δ40p53β
Δ160p53α
Δ133p53β
35
28
p53 – SAPU
High exposure

92.1

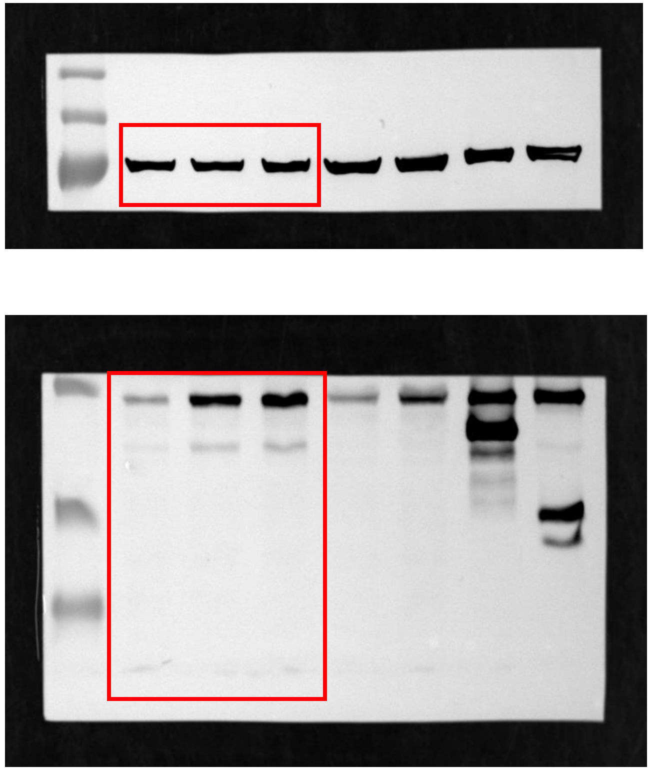

MEL270-A549

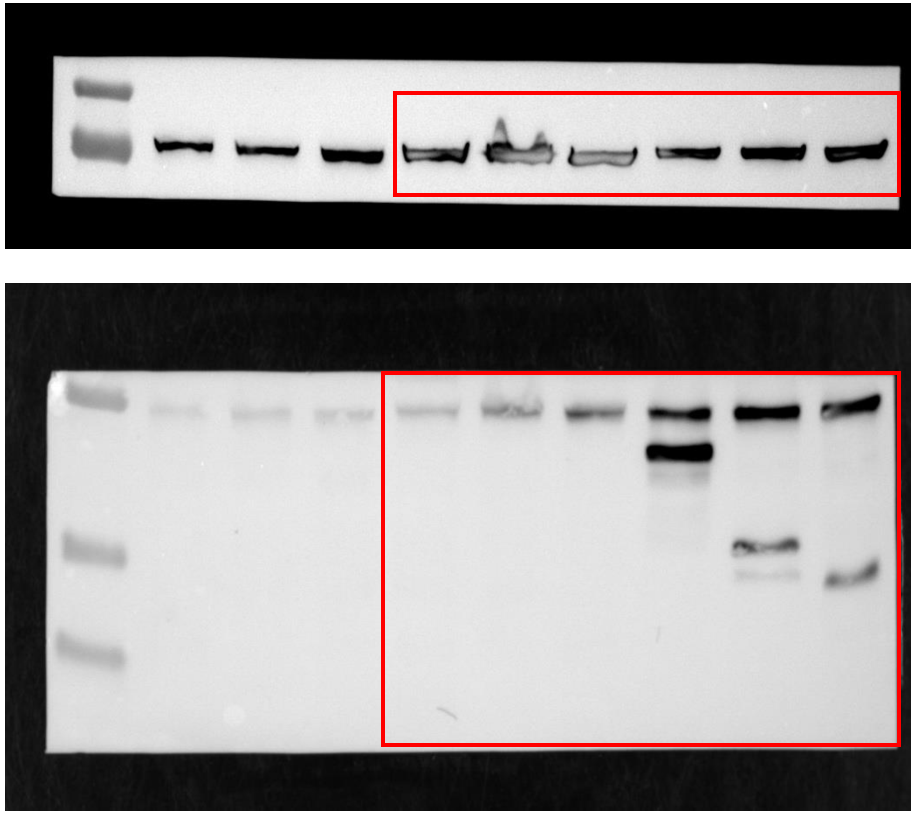

UPMD1

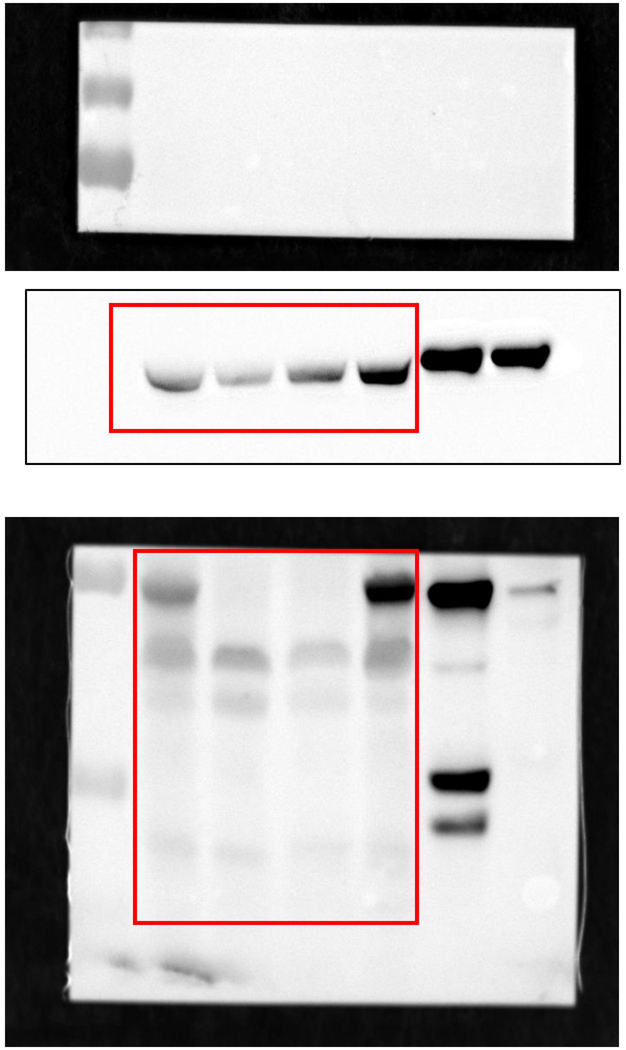

Figure 2C

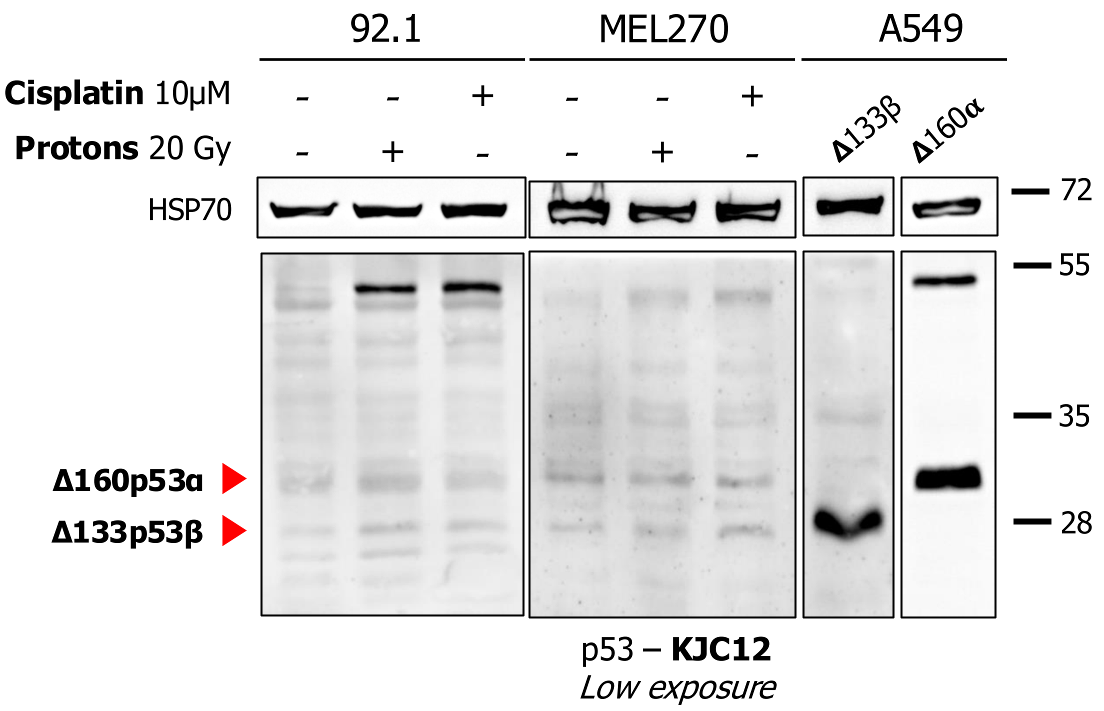
92.1
MEL270
A549
Cisplatin 10µM
Protons 20 Gy
Δ133β
Δ160α
HSP70
72
55
35
28
Δ160p53α
Δ133p53β
p53 – KJC12
Low exposure

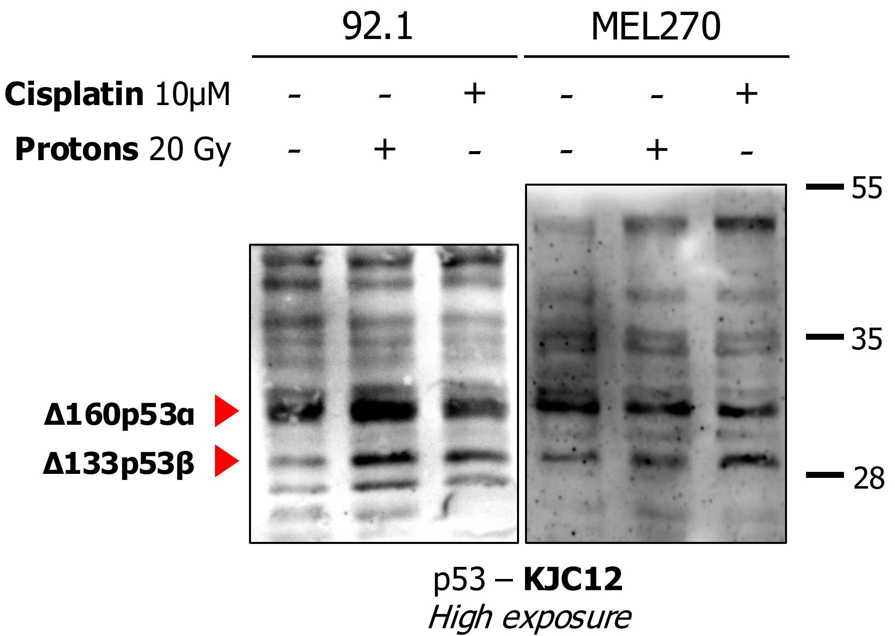
92.1
MEL270
Cisplatin 10µM
Protons 20 Gy
55
35
28
Δ160p53α
Δ133p53β
p53 – KJC12
High exposure

92.1-A549Δ160α

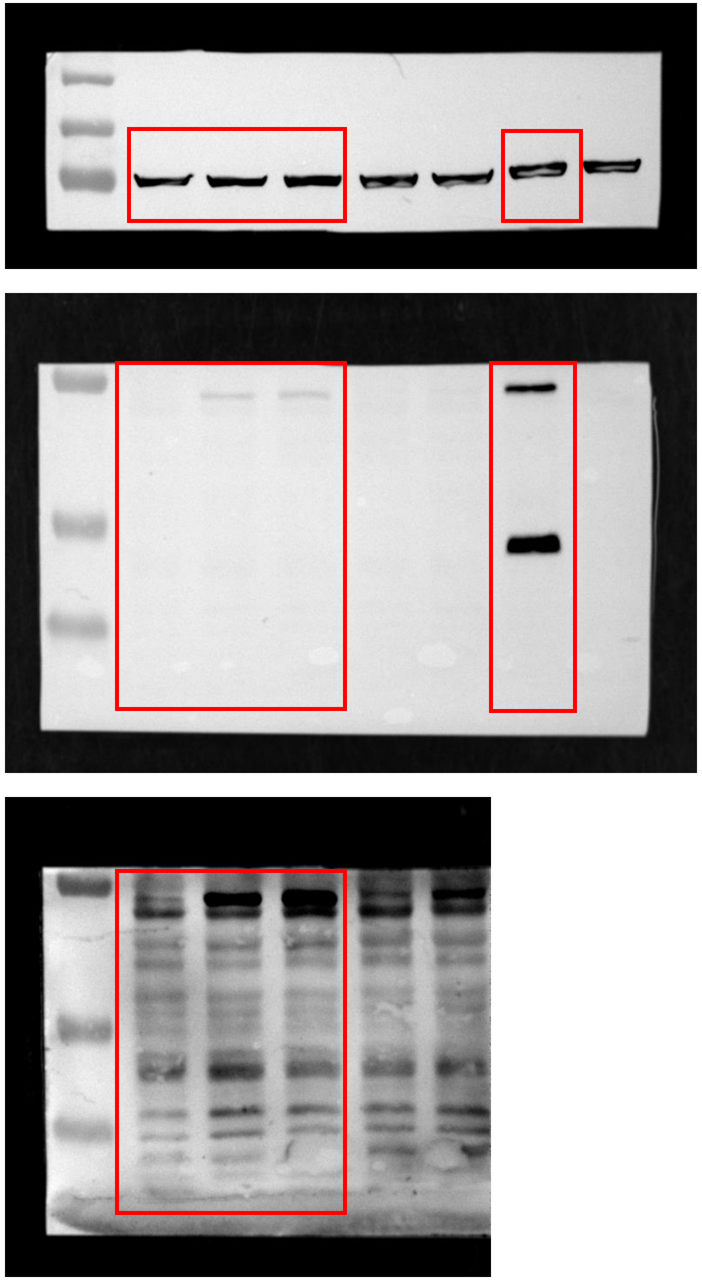

MEL270-A549Δ133β

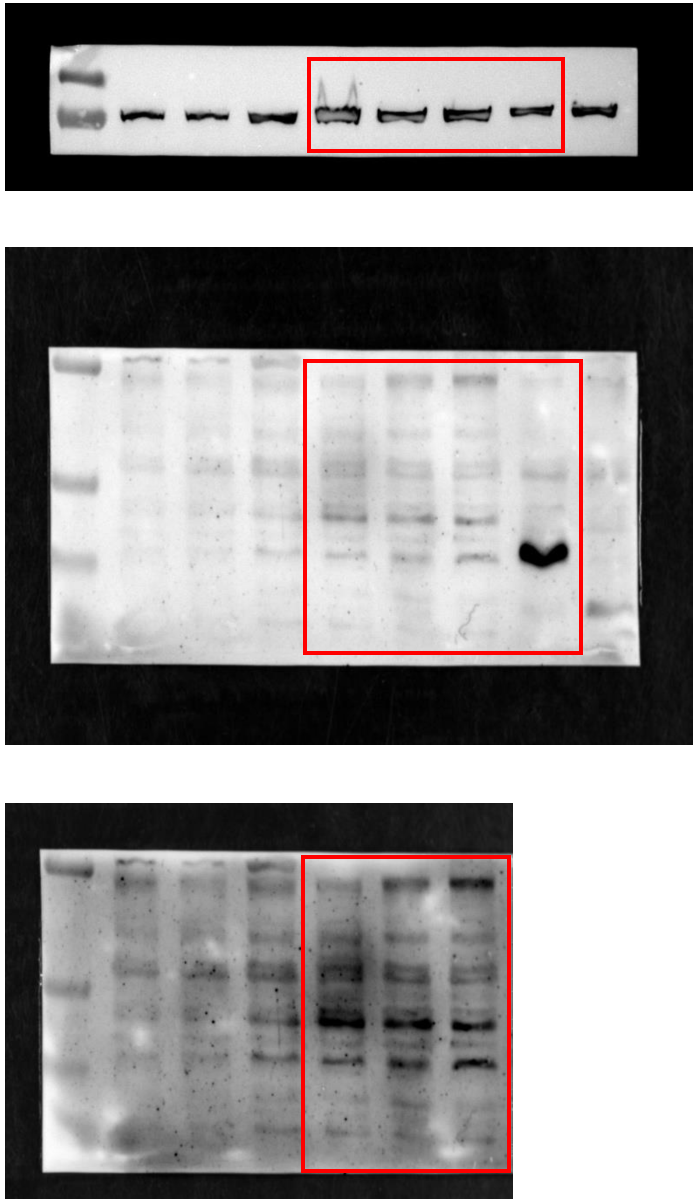

Figure 2E

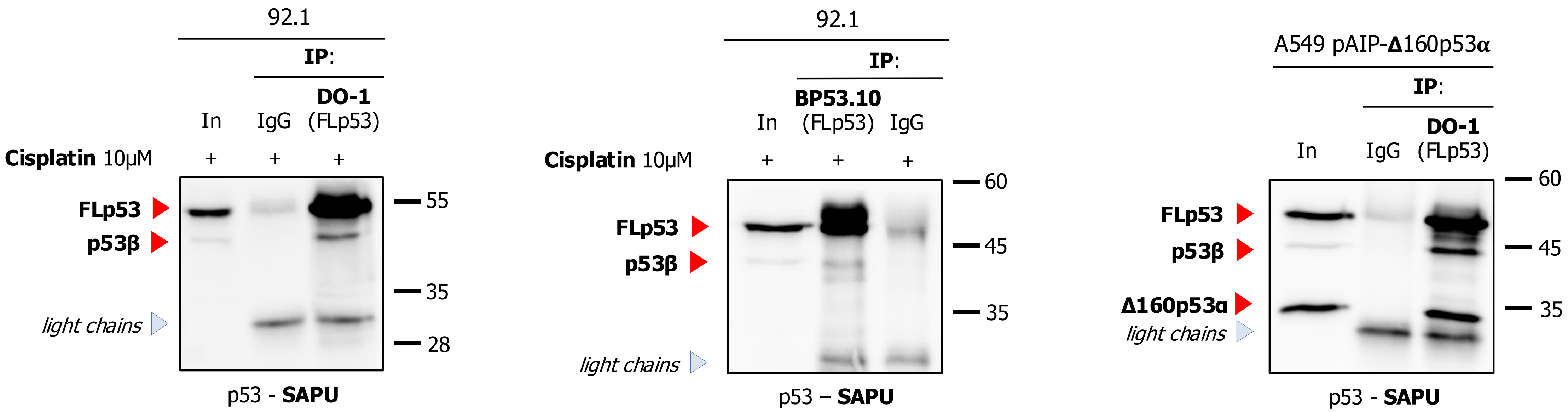
92.1
IP:
DO-1
In
IgG
(FLp53)
Cisplatin 10µM
+
+
+
FLp53
p53β
light chains
55
35
28
p53 - SAPU
92.1
IP:
BP53.10
In
(FLp53)
IgG
Cisplatin 10µM
+
+
+
FLp53
p53β
light chains
60
45
35
p53 – SAPU
A549 pAIP-Δ160p53α
IP:
DO-1
In
IgG
(FLp53)
FLp53
p53β
Δ160p53α
light chains
60
45
35
p53 - SAPU

Figure 2E

92.1

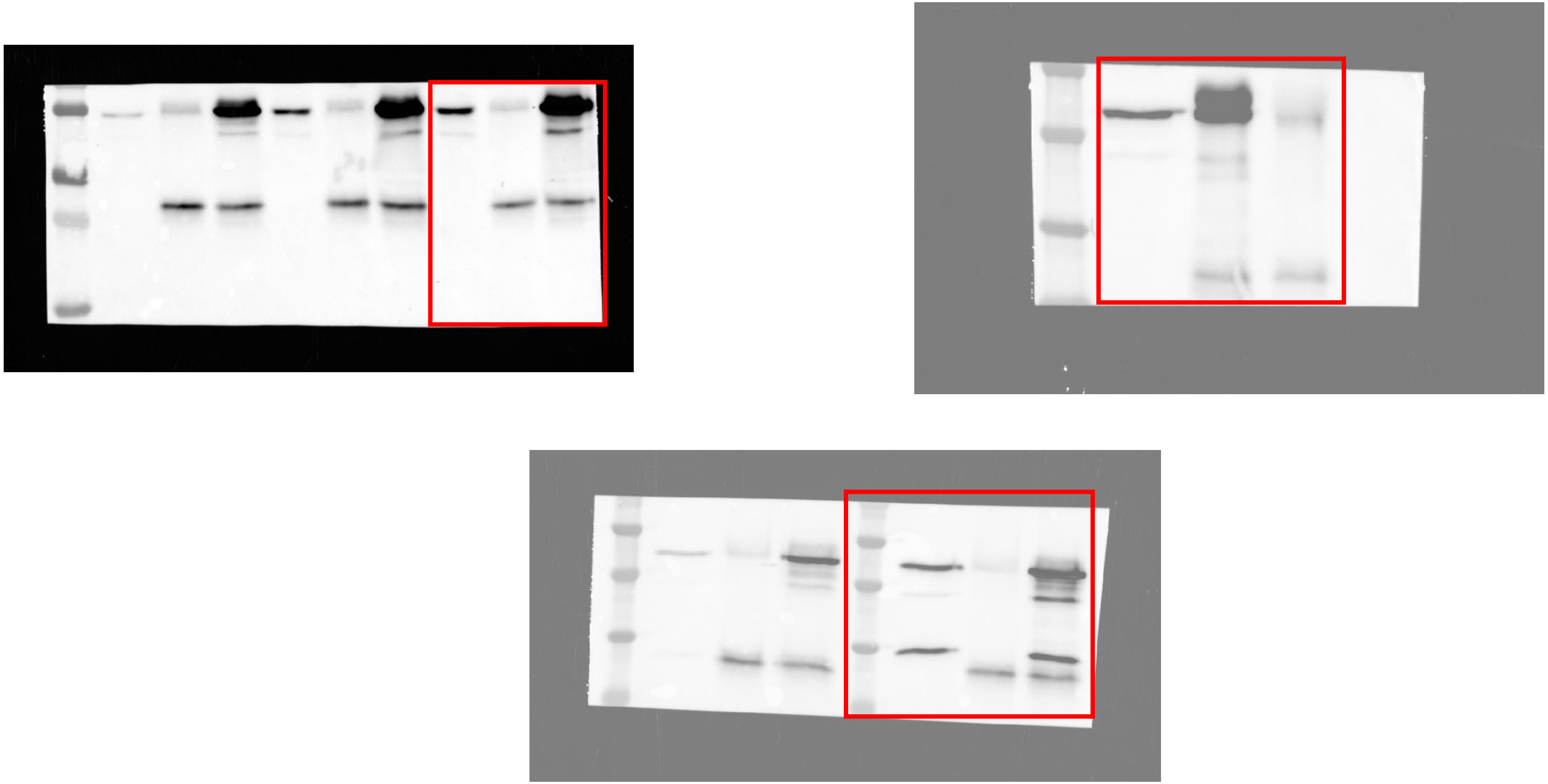

Figure 3A

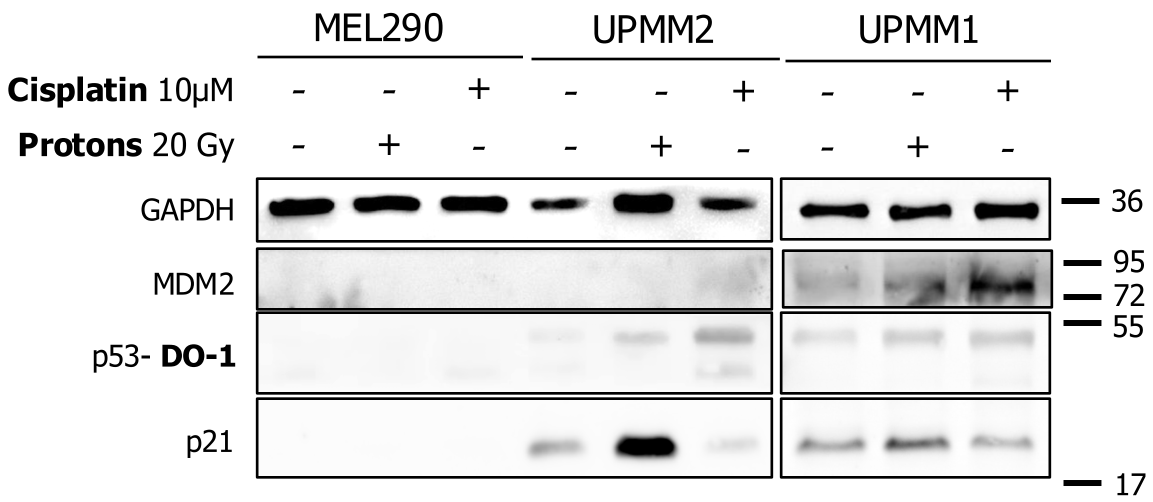
MEL290
UPMM2
UPMM1
Cisplatin 10µM - - + - - + - - +
Protons 20 Gy - + - - + - - + -
GAPDH
MDM2
p53- DO-1
p21
36
95
72
55
17

Figure 3A

MEL290-UPMM2

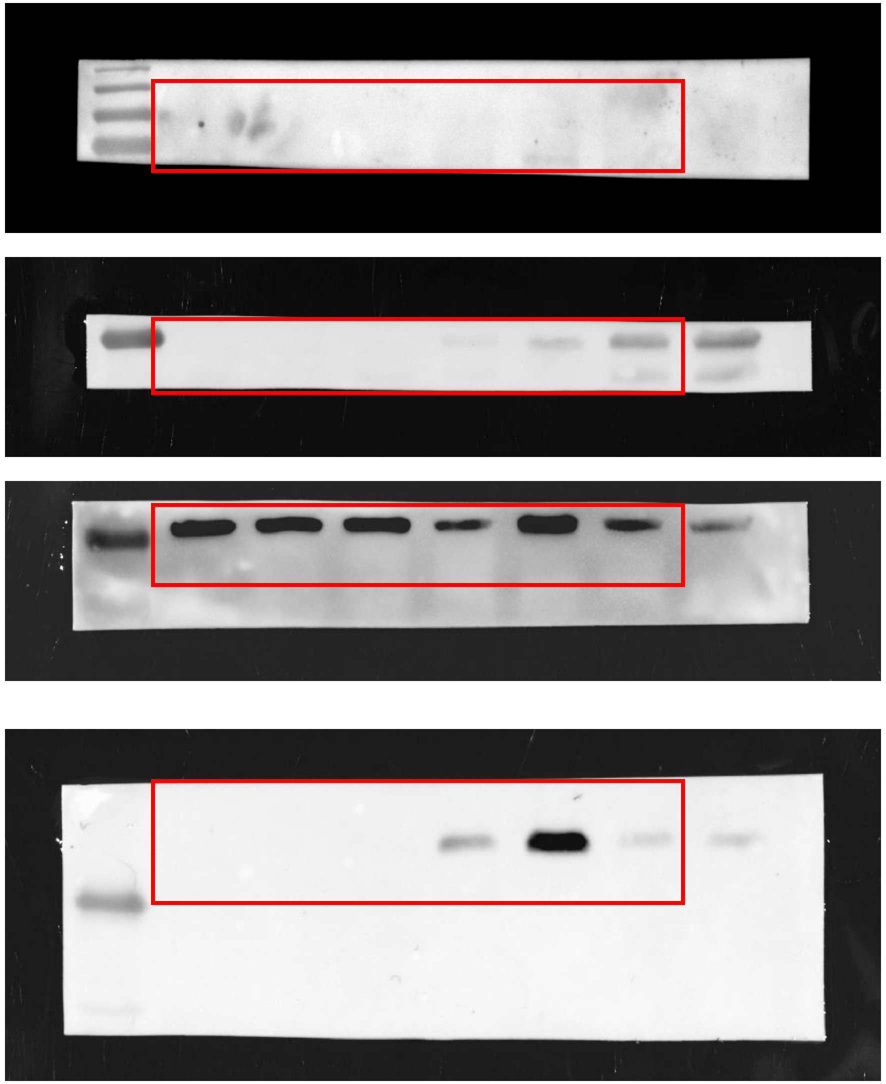

UPMM1

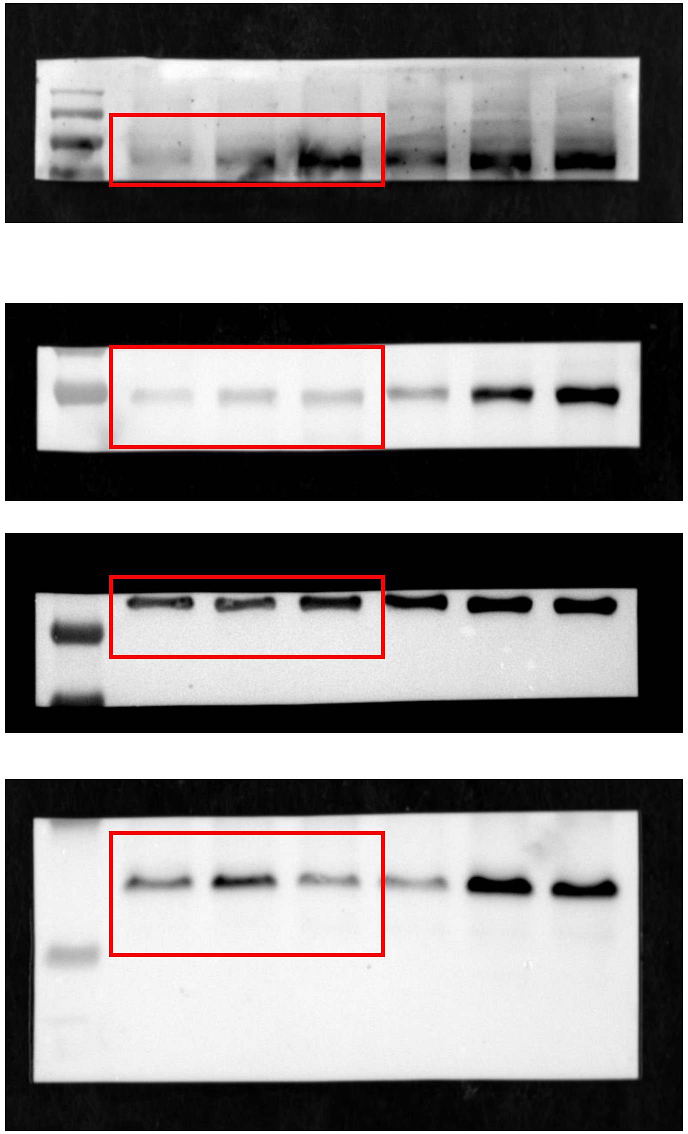

Figure 3B

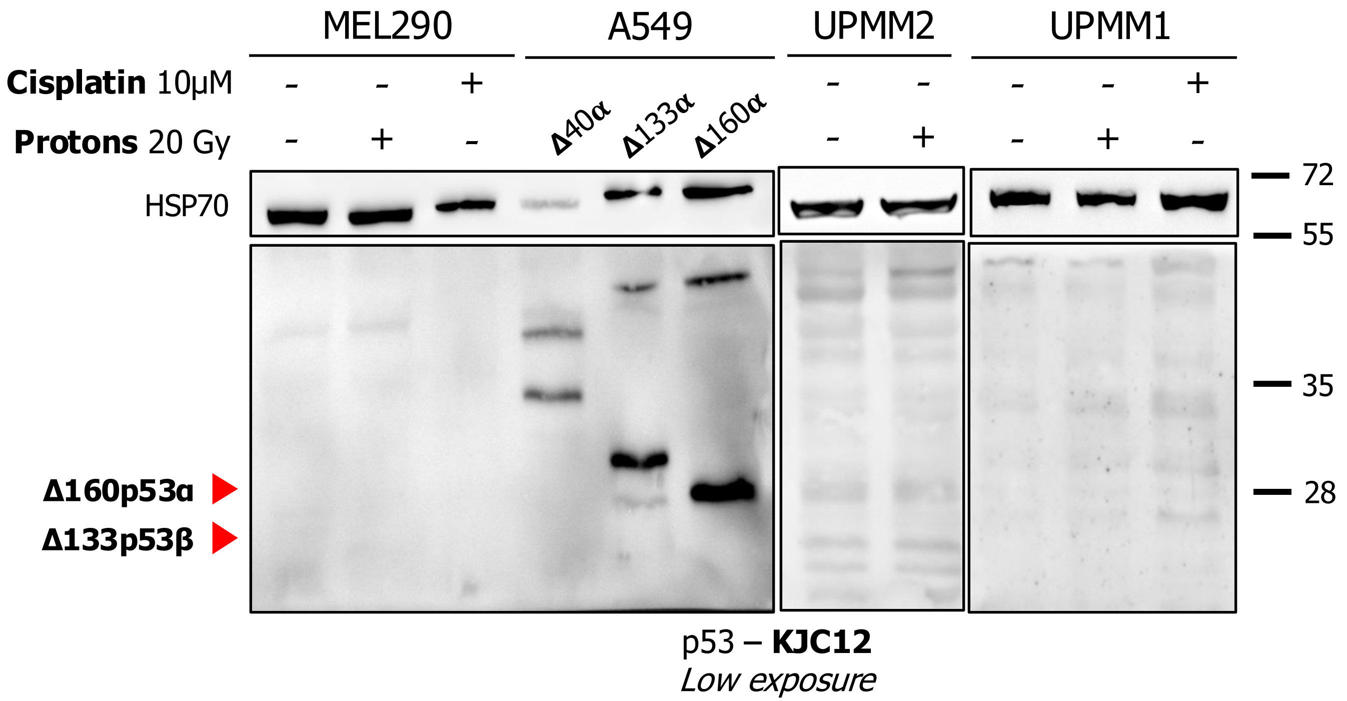
MEL290
A549
UPMM2
UPMM1
Cisplatin 10µM
Protons 20 Gy
Δ40α
Δ133α
Δ160α
HSP70
72
55
35
28
Δ160p53α
Δ133p53β
p53 – KJC12
Low exposure

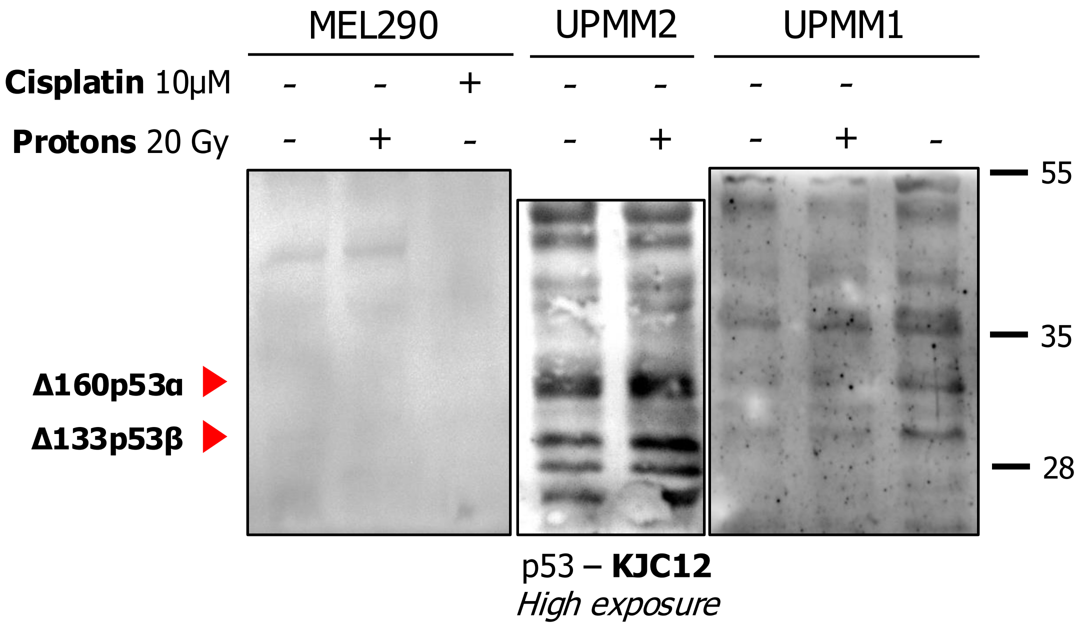
MEL290
UPMM2
UPMM1
Cisplatin 10µM
Protons 20 Gy
55
35
28
Δ160p53α
Δ133p53β
p53 – KJC12
High exposure

Figure 3B

MEL290- A549

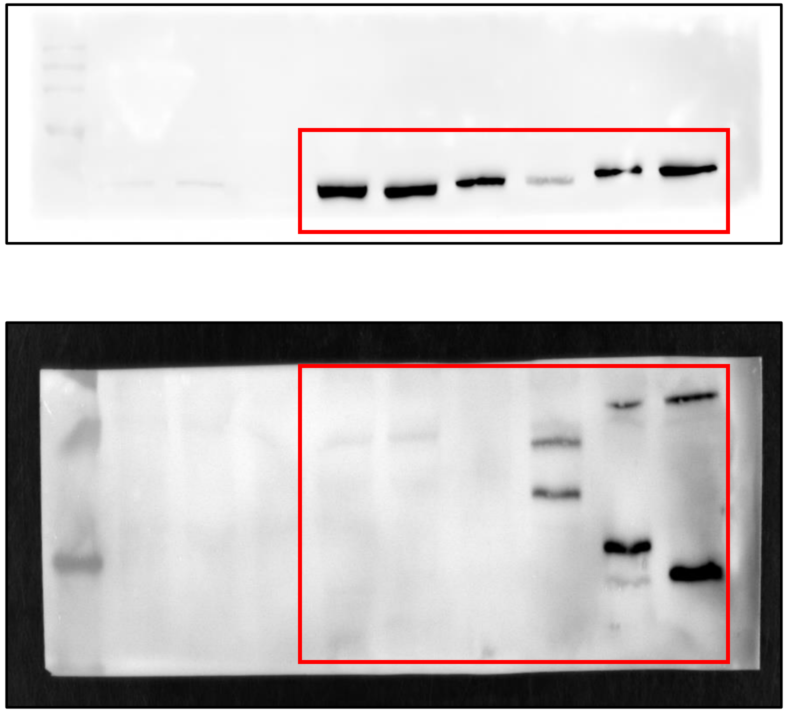

UPMM2

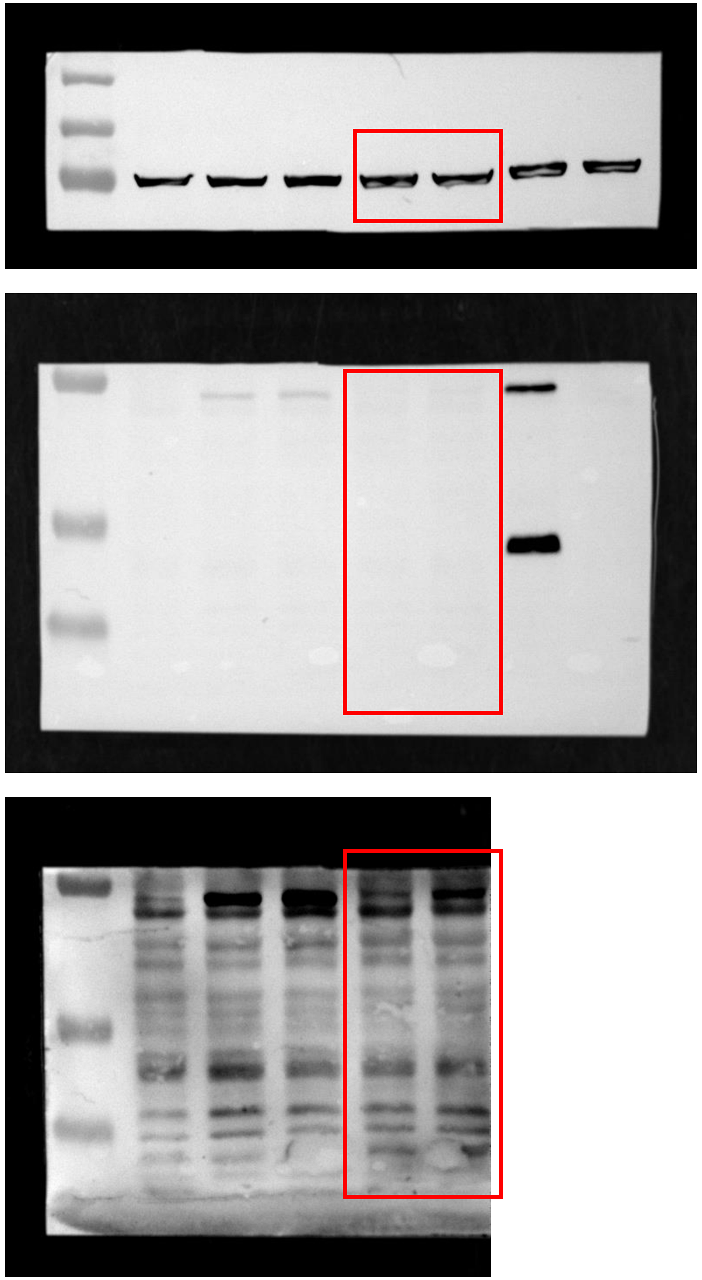

UPMM1

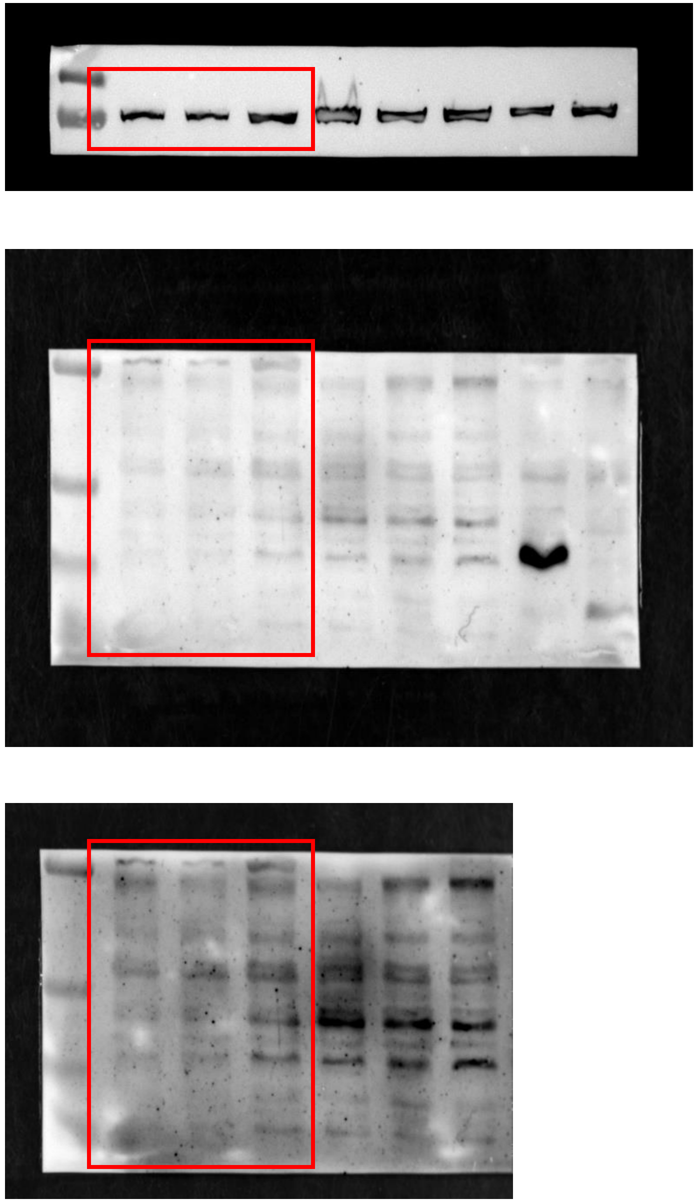

Figure 5D

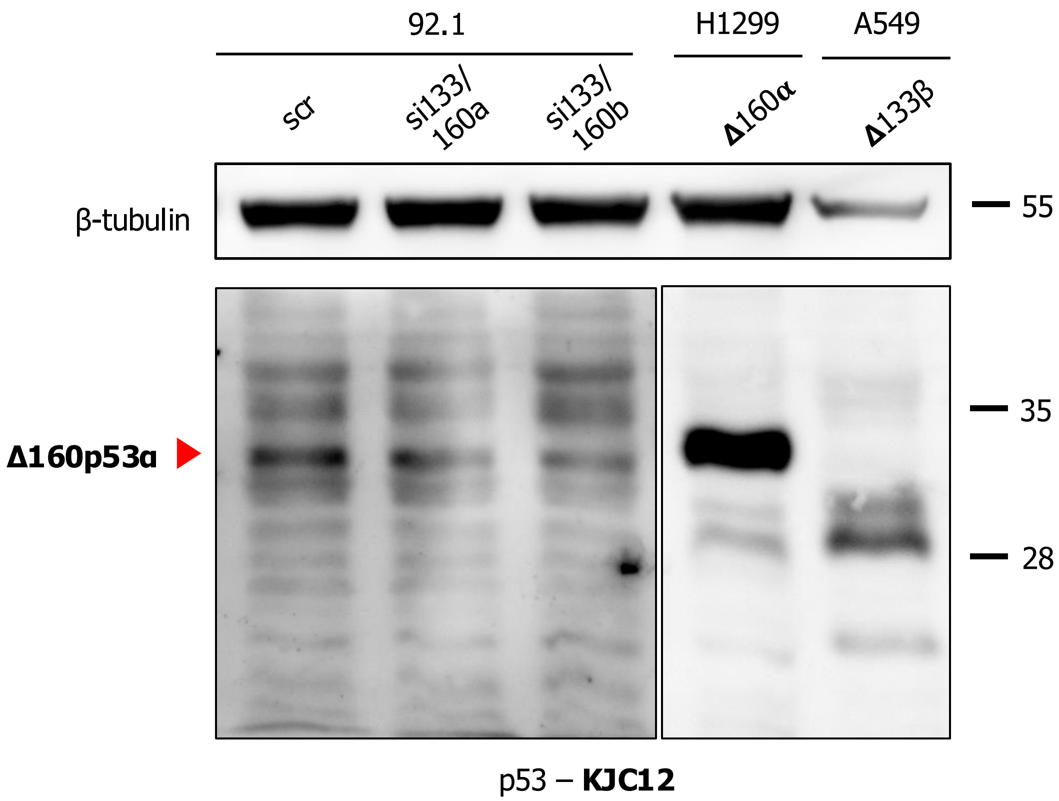
92.1
H1299
A549
scr
si133/160a
si133/160b
Δ160α
Δ133β
β-tubulin
55
35
28
Δ160p53α
p53 – **KJC12**

Figure 5D

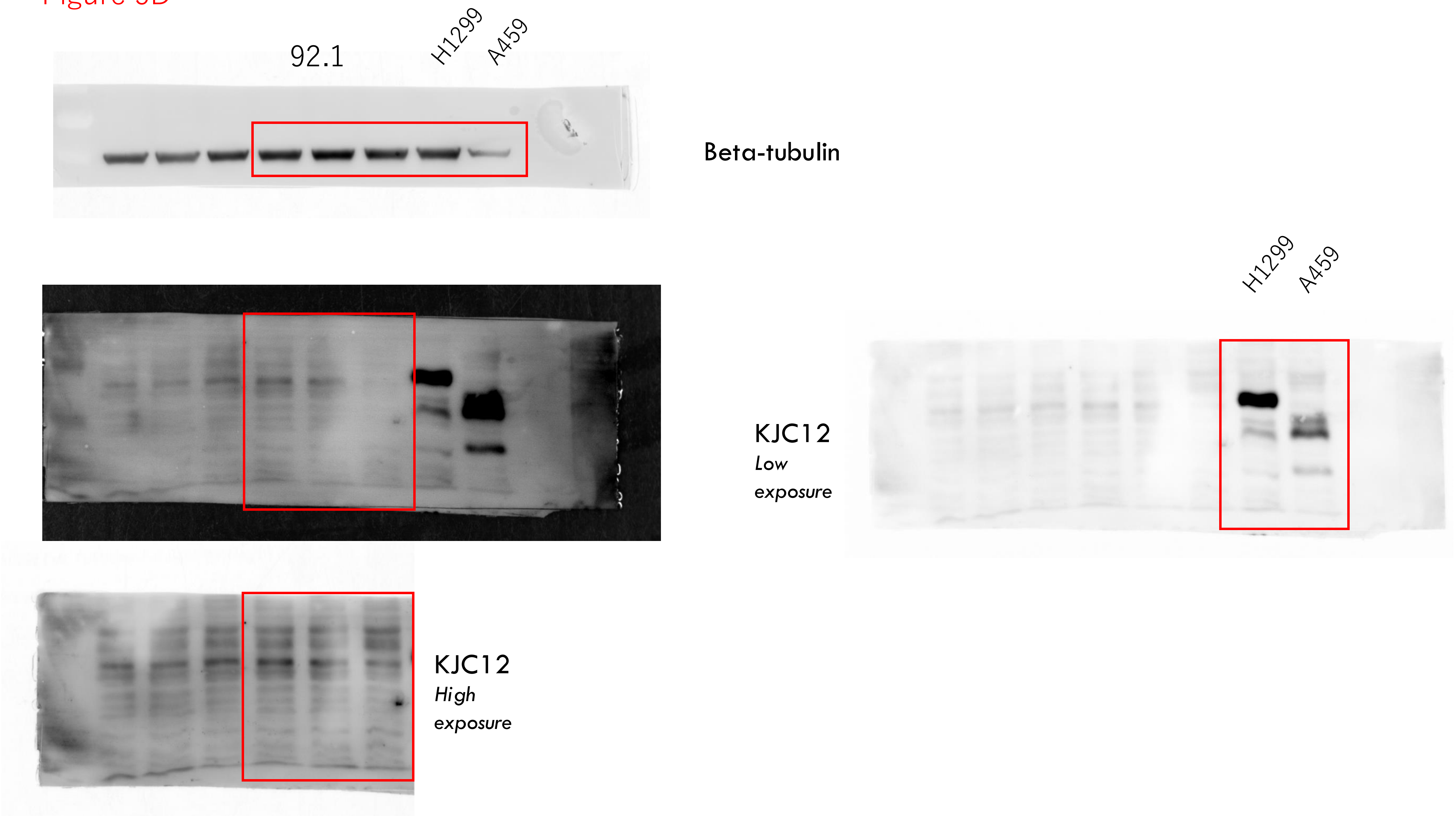
92.1
H1299
A459
Beta-tubulin
KJC12
Low exposure
KJC12
High exposure
H1299
A459

Supplementary Figure 4

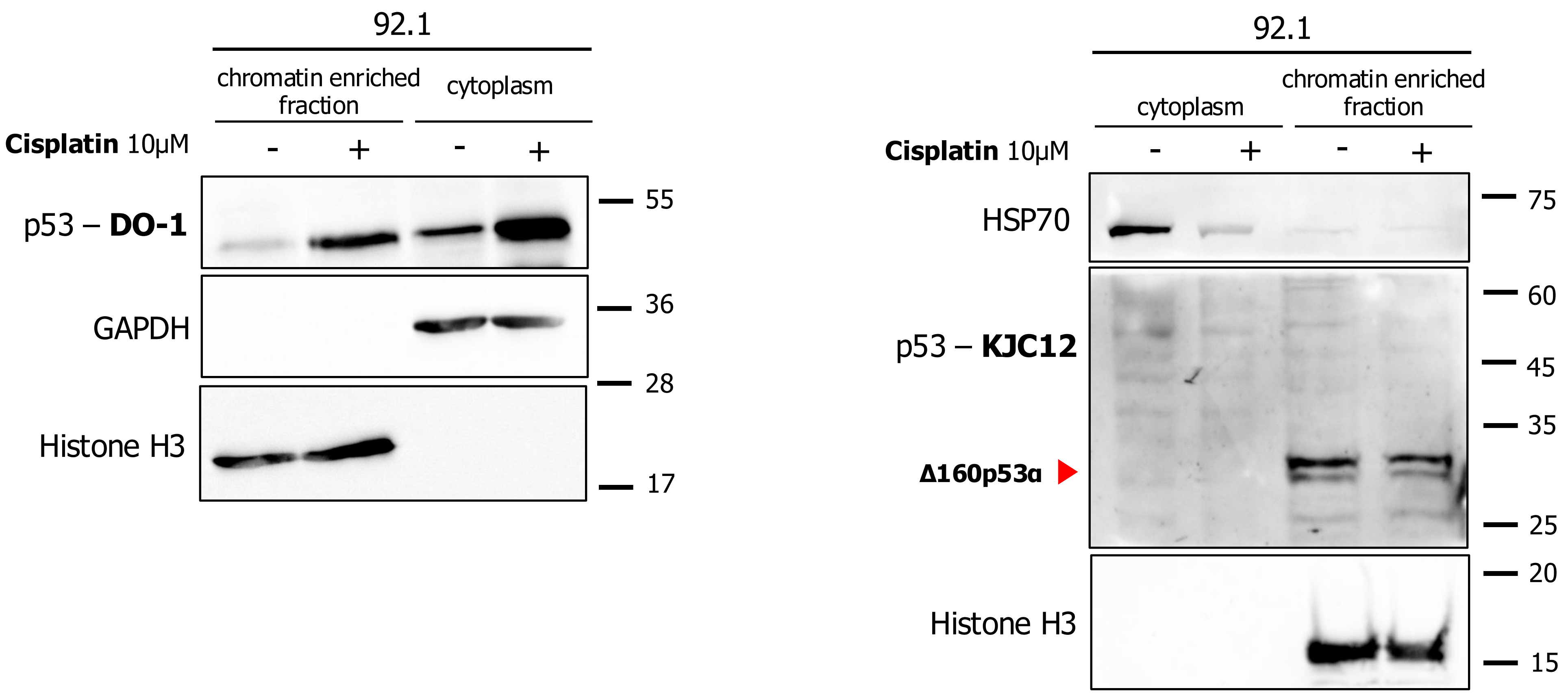

Supplementary Figure 4

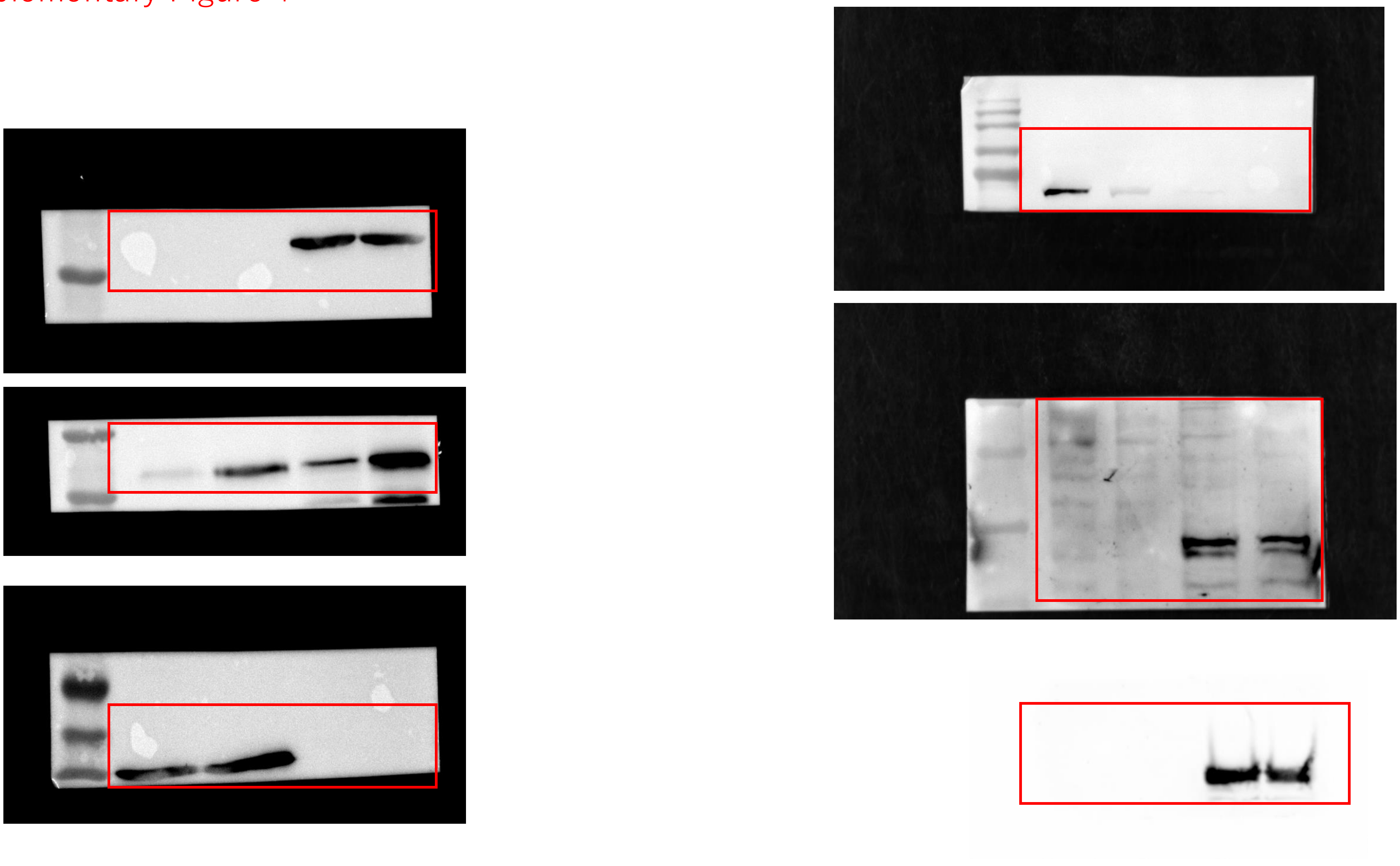

Supplementary Figure 5

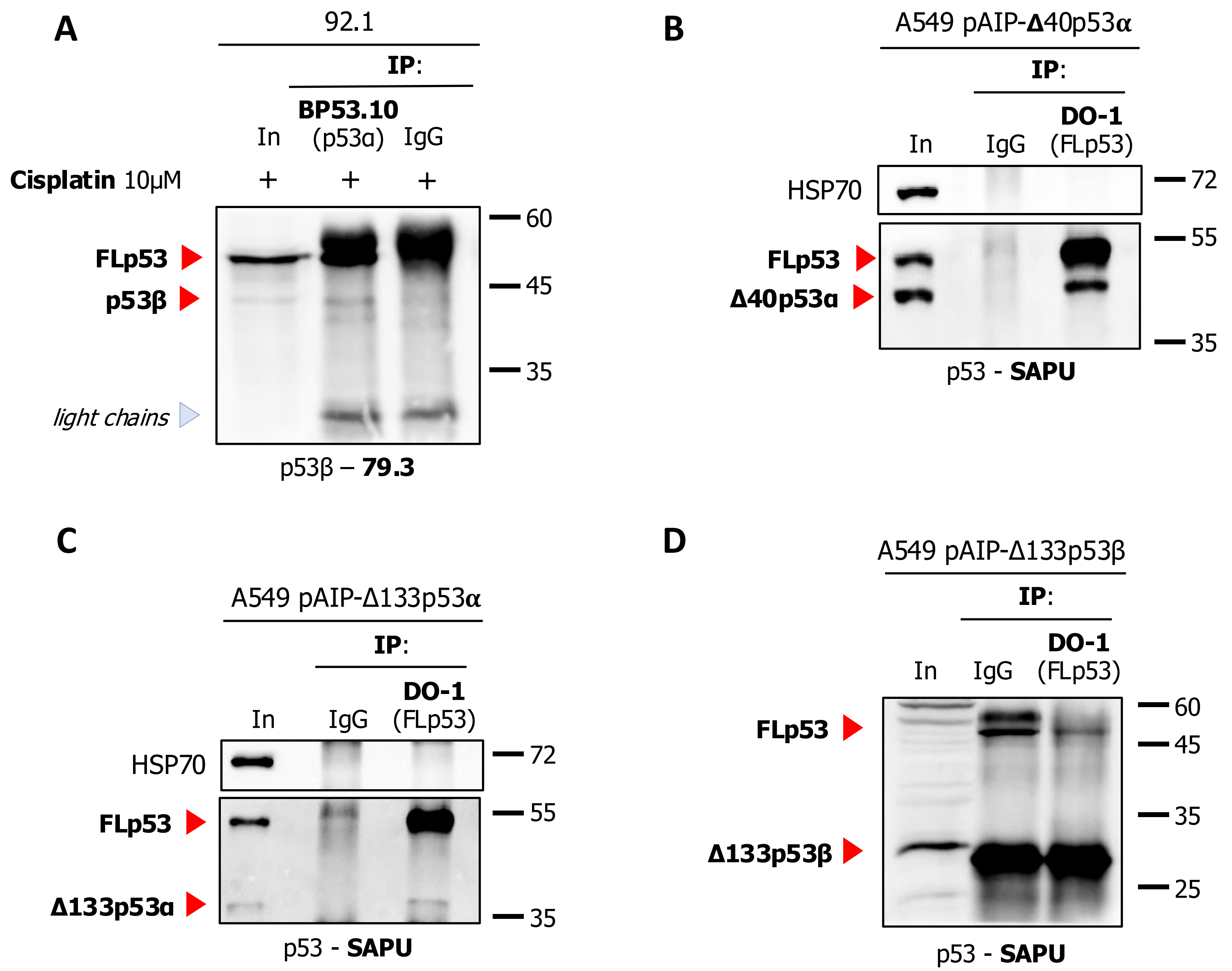

Supplementary Figure 5

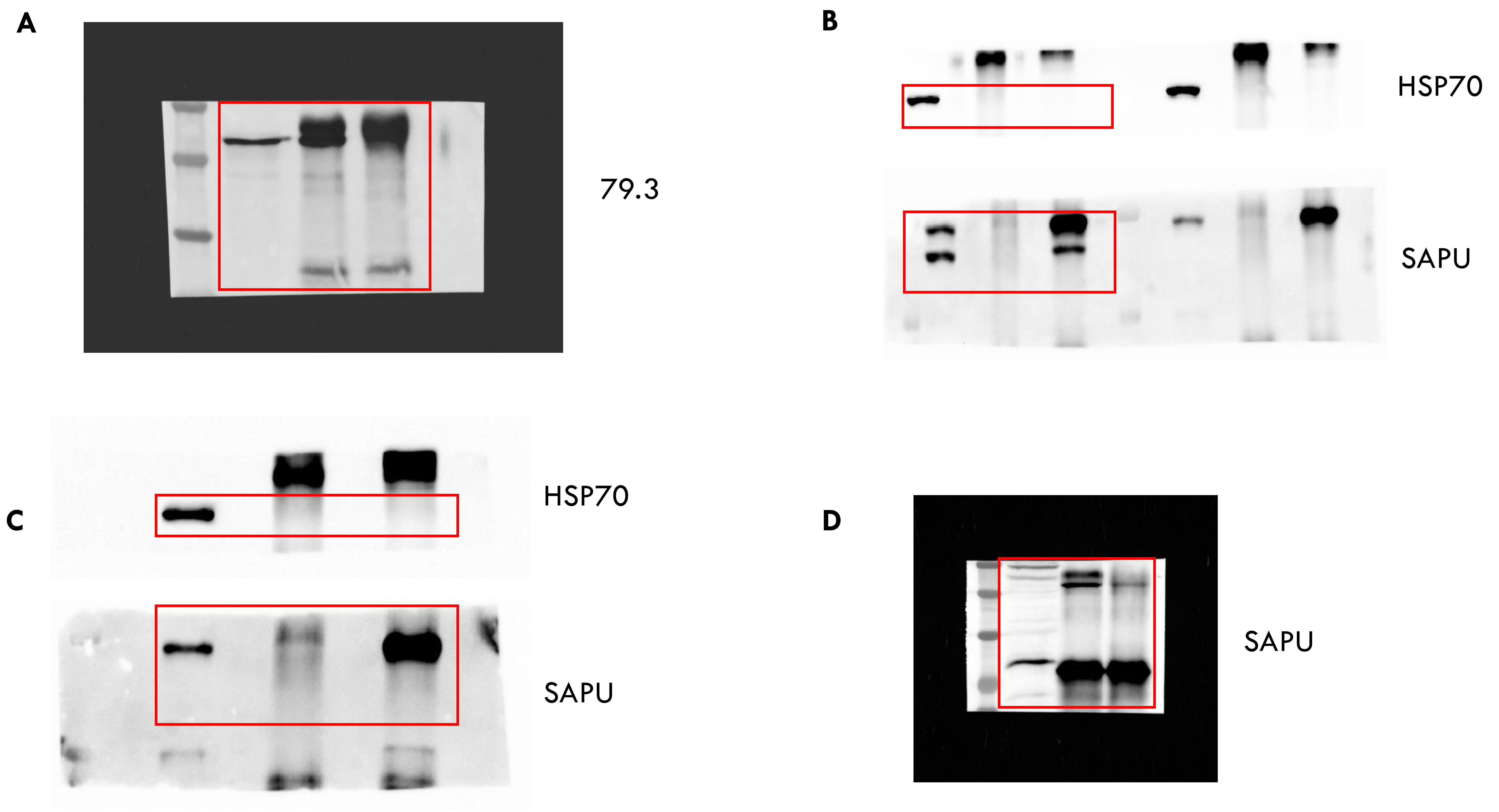

Supplementary Figure 6A

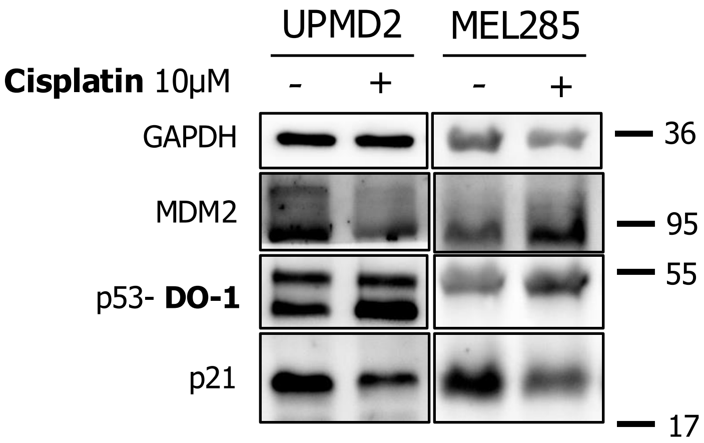

Supplementary Figure 6

UPMD2

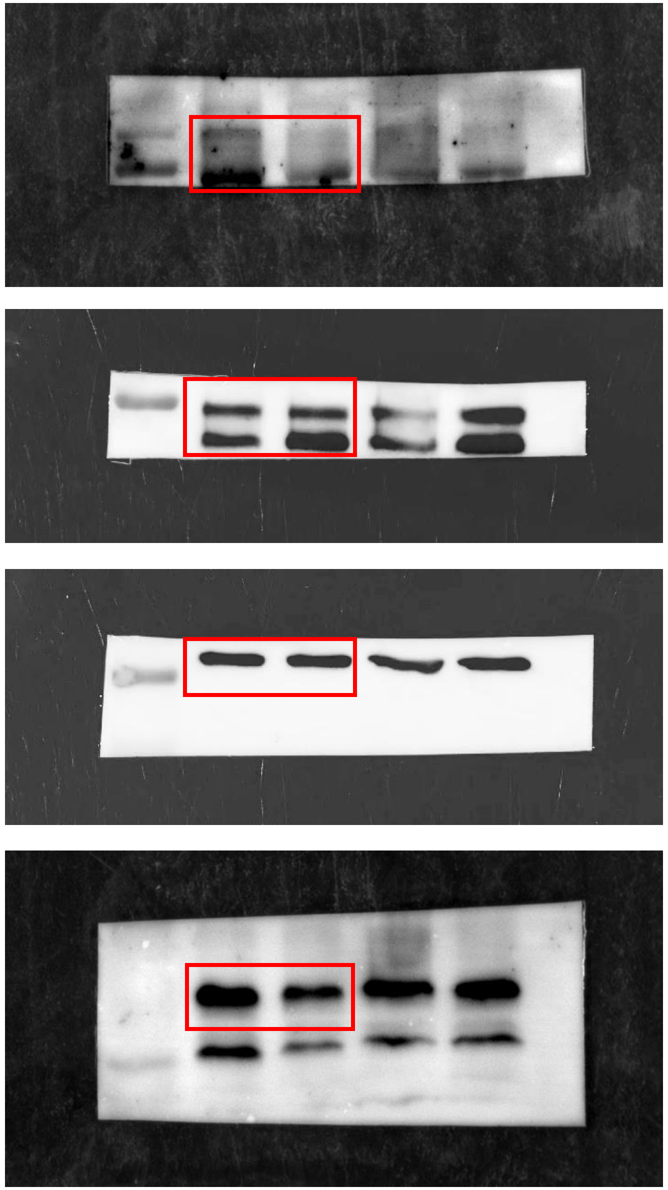

MEL285

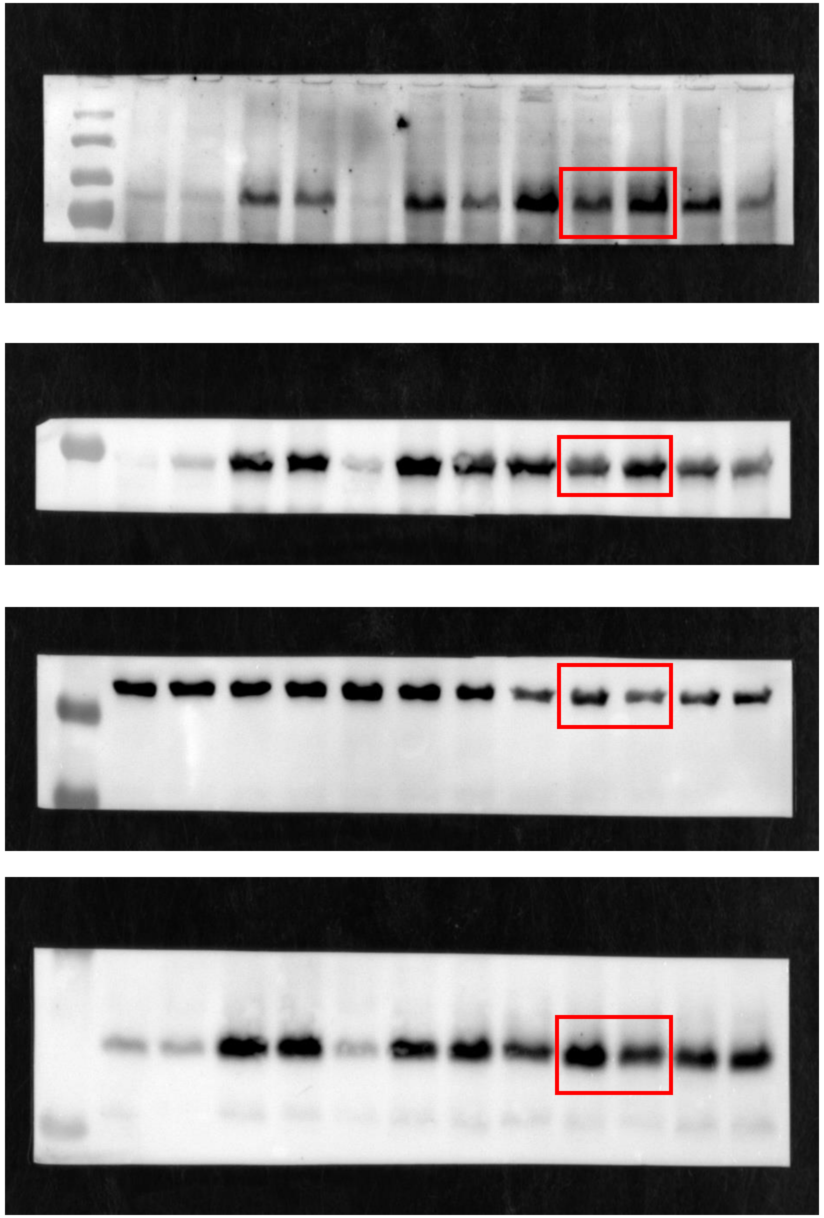

Supplementary Figure 6B

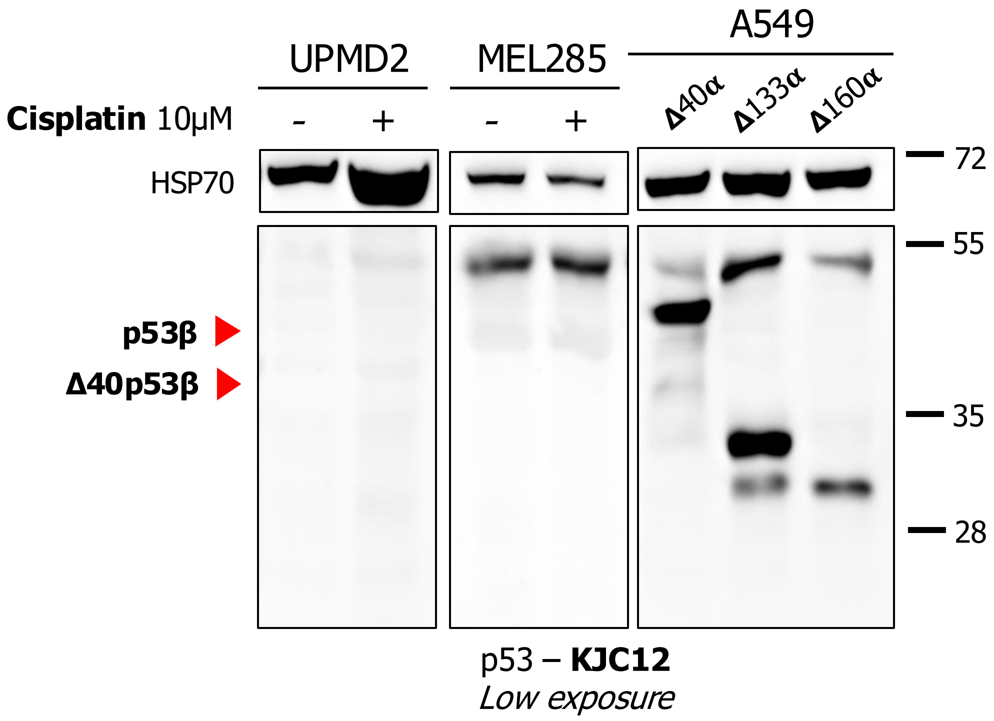

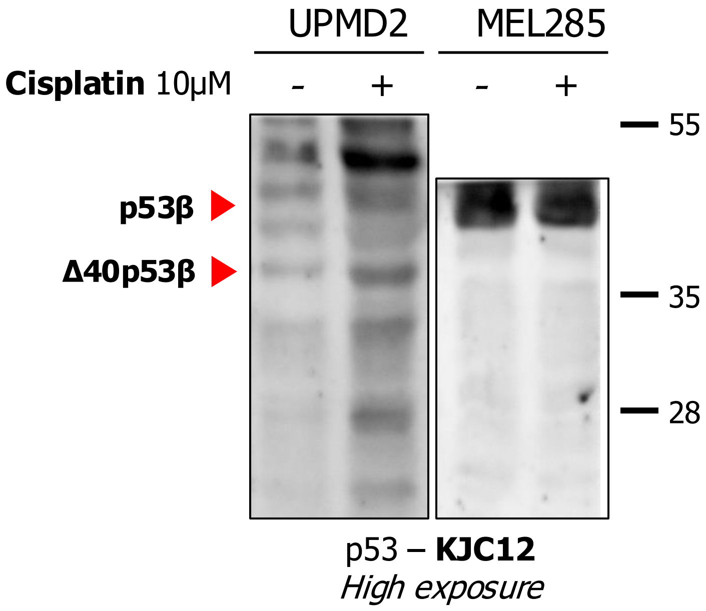

Supplementary Figure 6

UPMD2-A549

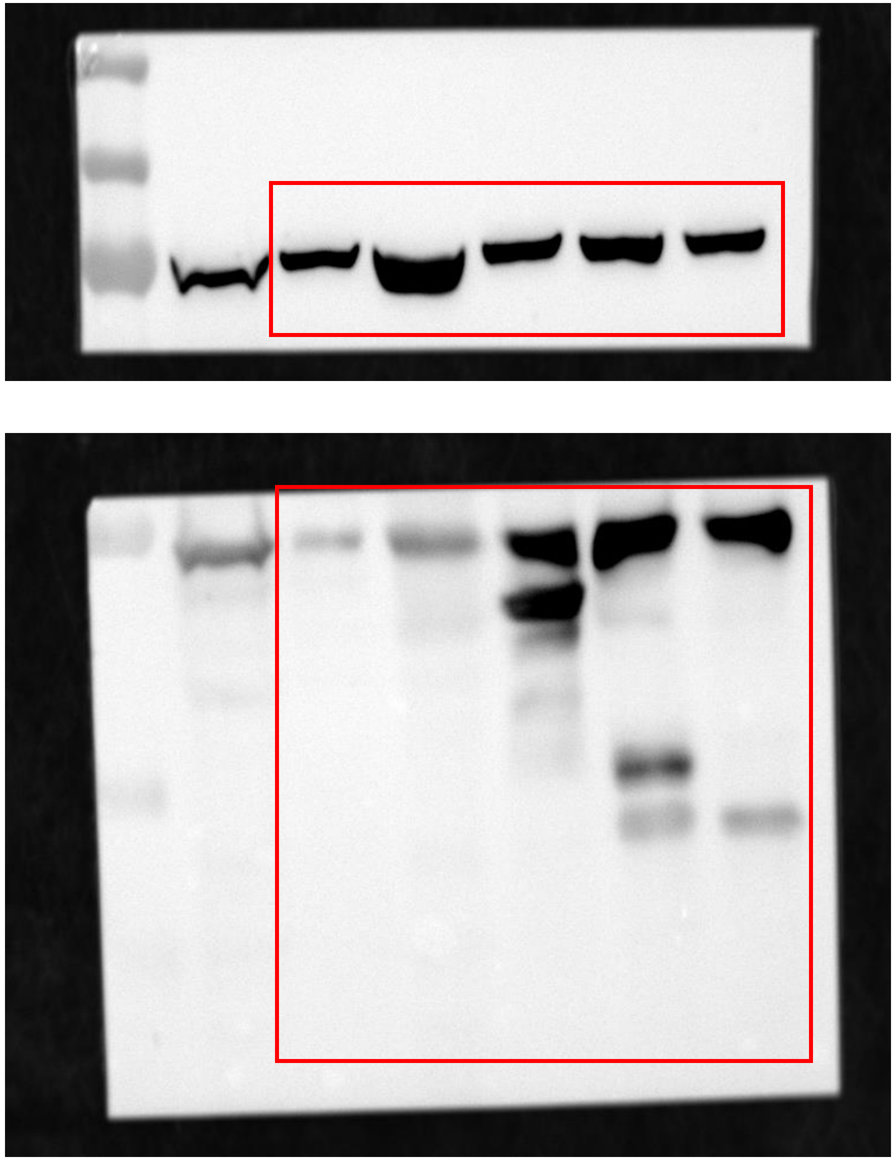

MEL285

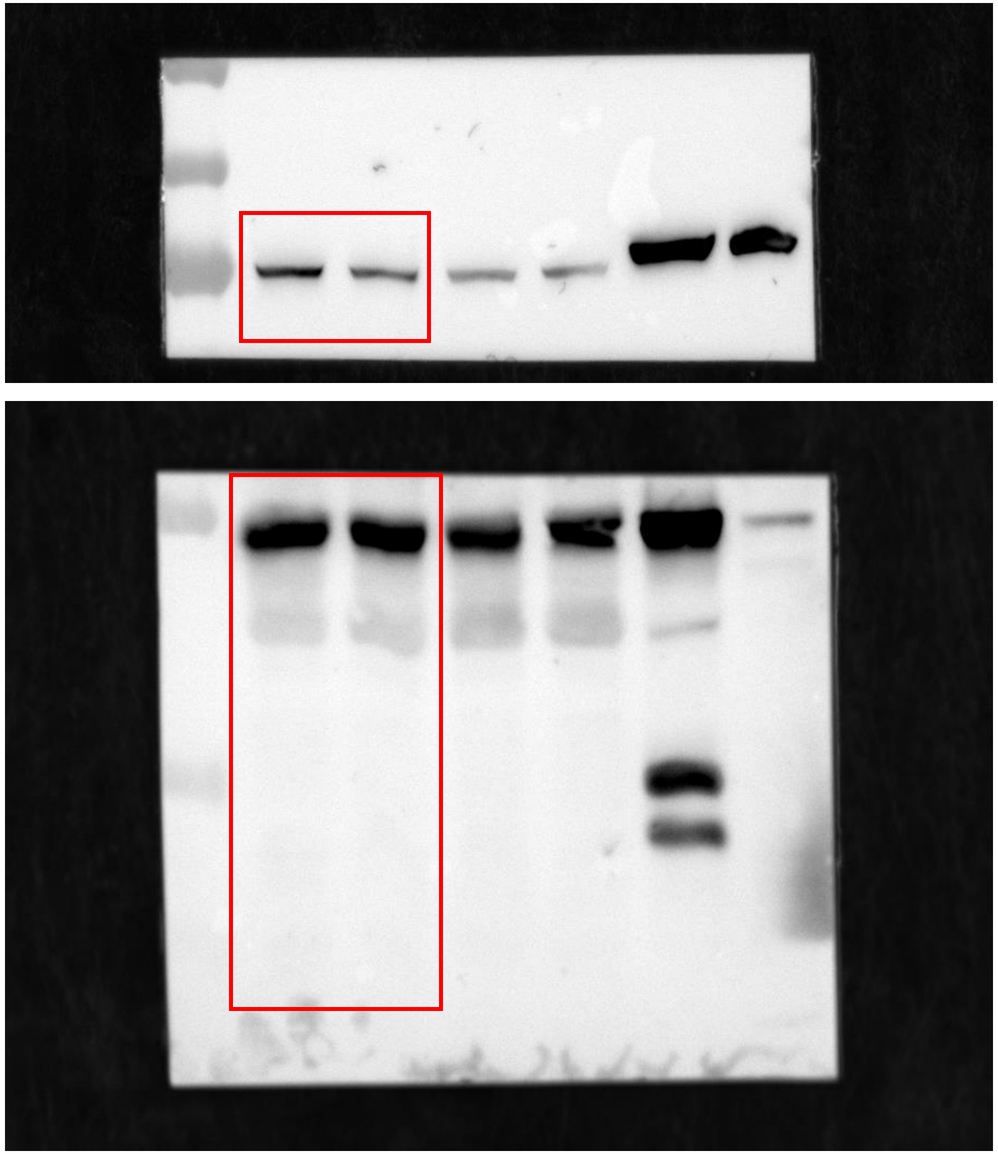

Supplementary Figure 7A

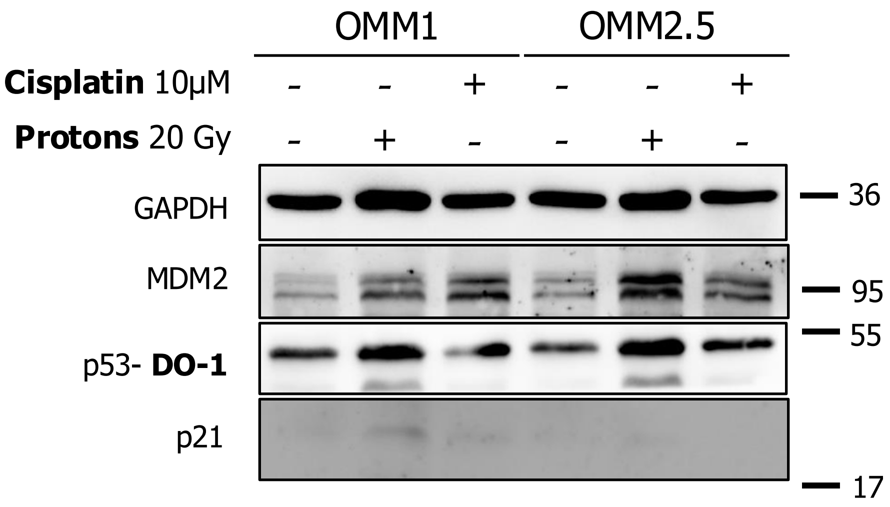

Supplementary Figure 7A

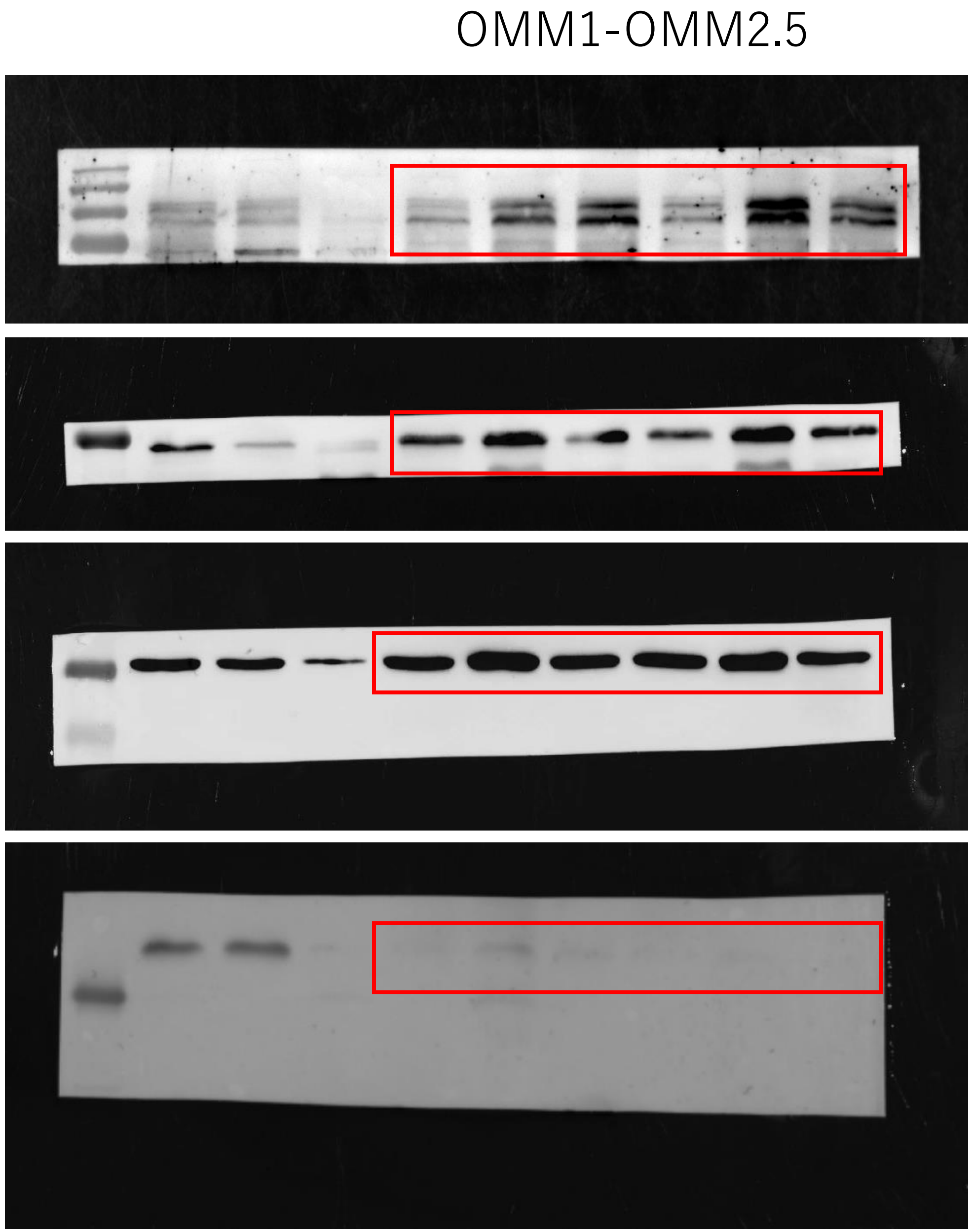

Supplementary Figure 7B

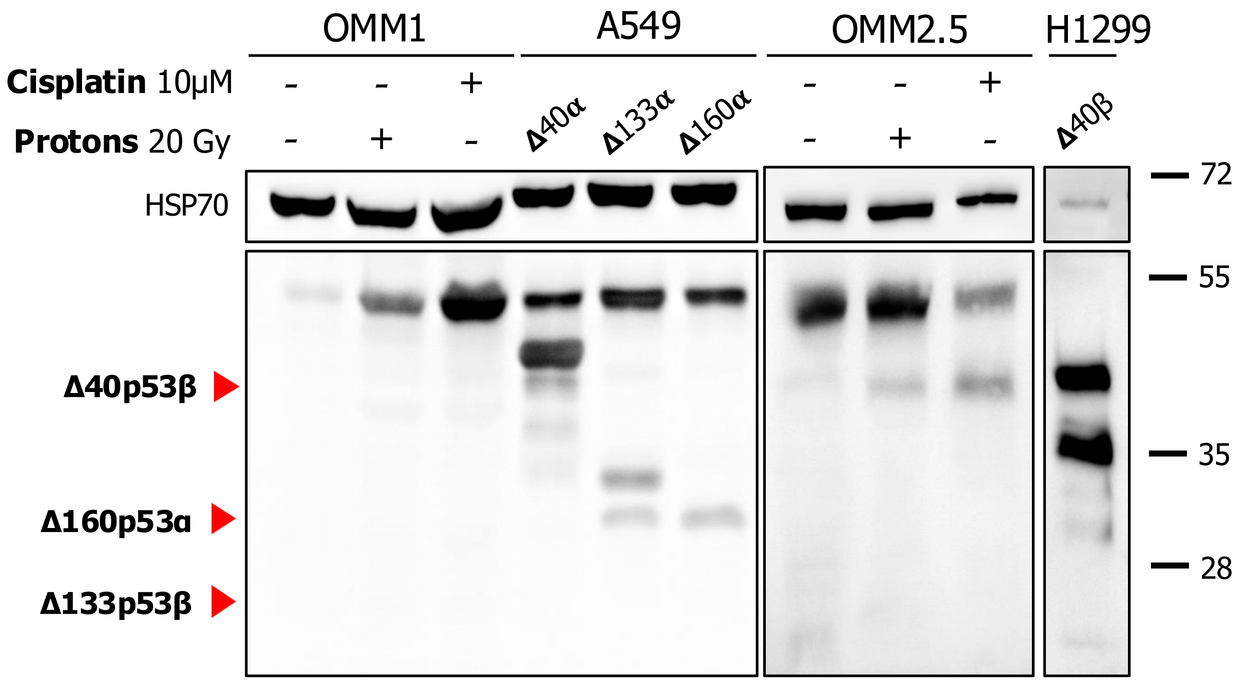

p53 – **SAPU**
Low exposure

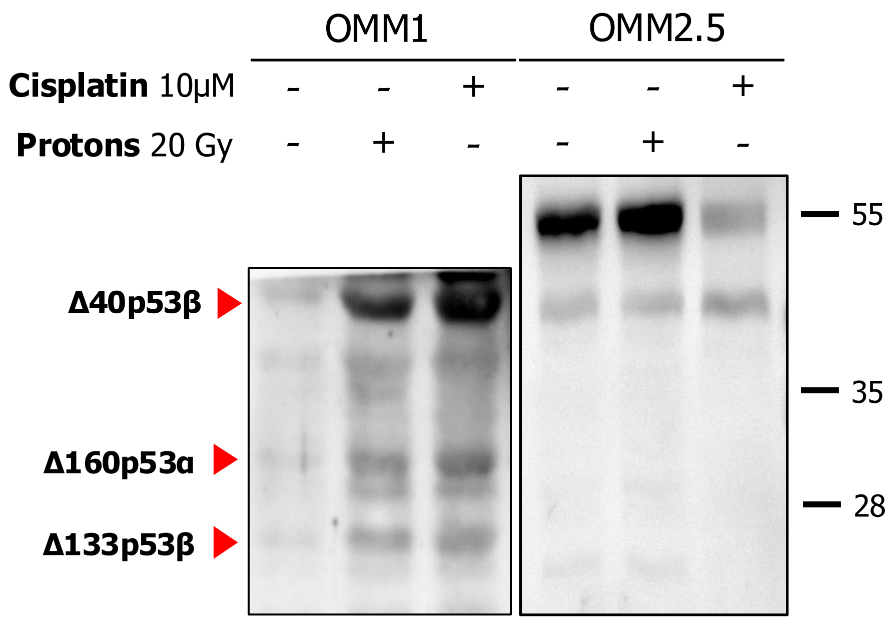

p53 – **SAPU**
High exposure

Supplementary Figure 7B

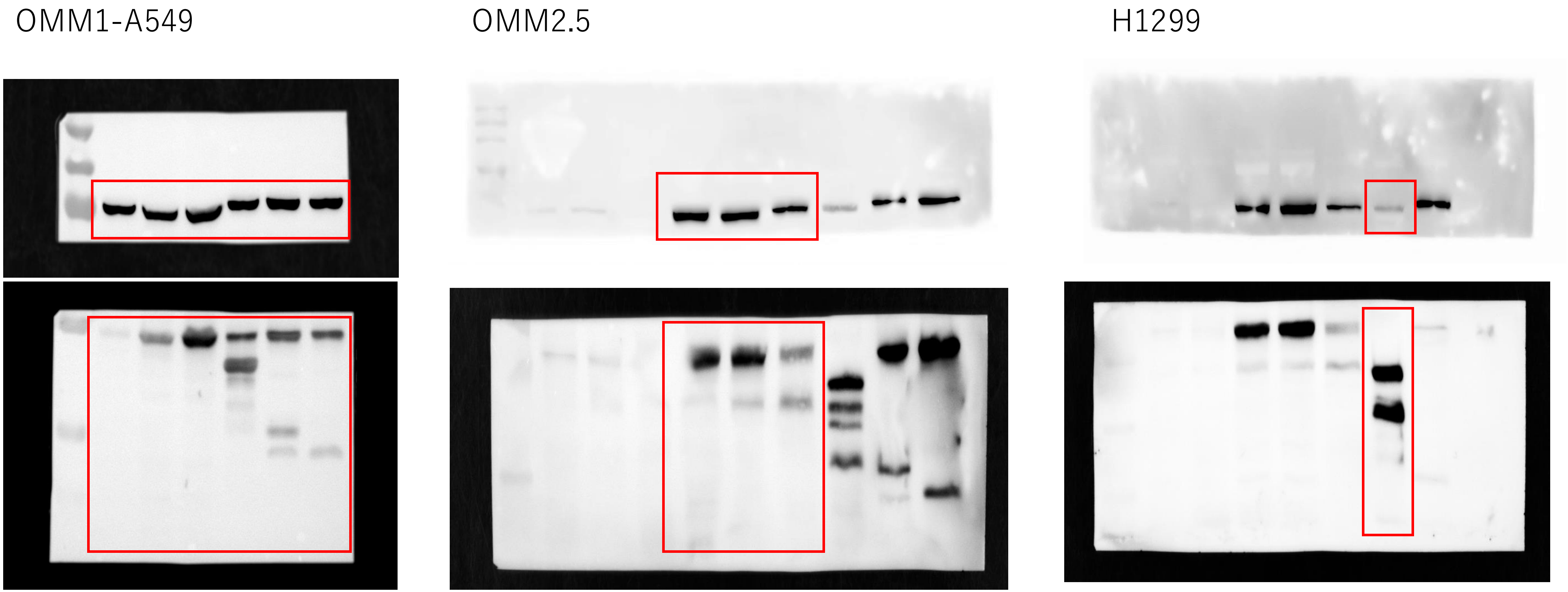

Supplementary Figure 7C

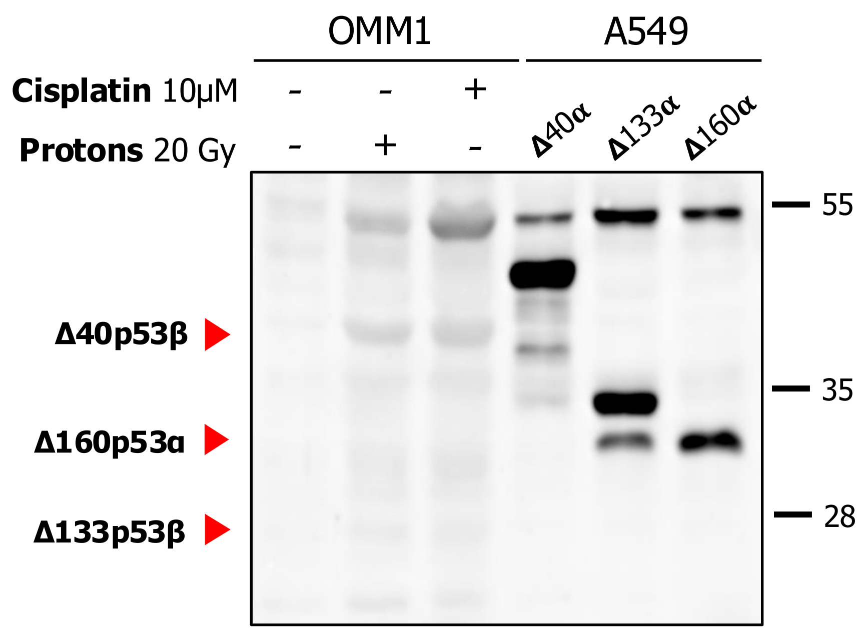

p53 – **KJC12**
Low exposure

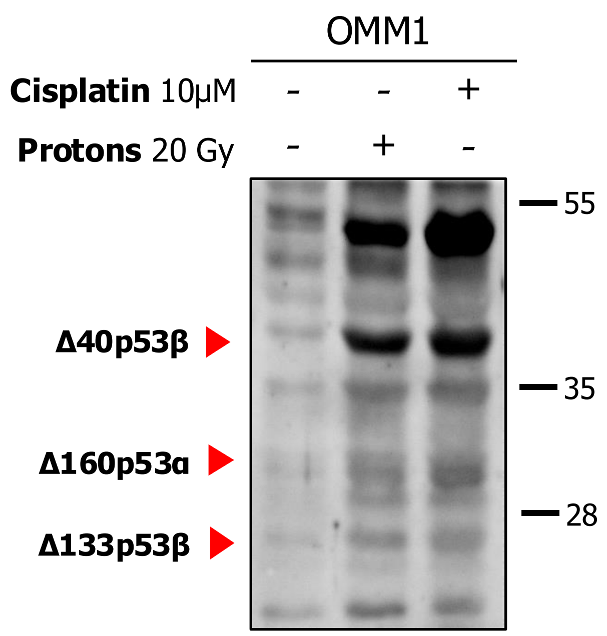

p53 – **KJC12**
High exposure

Supplementary Figure 7

OMM1-A549

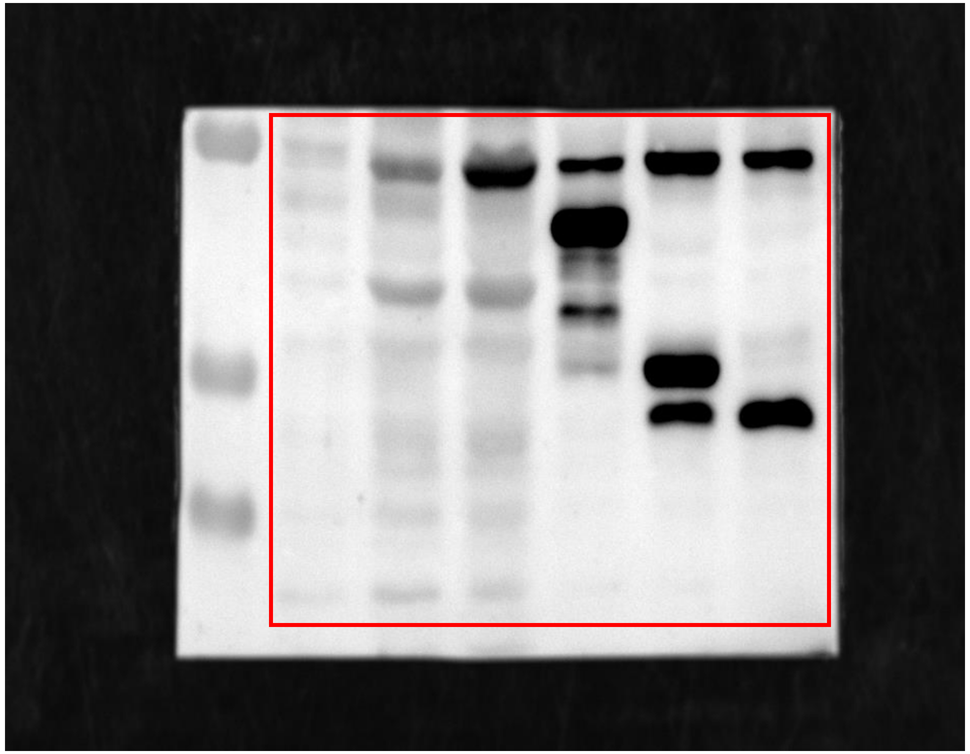

Supplementary Figure 8A-B

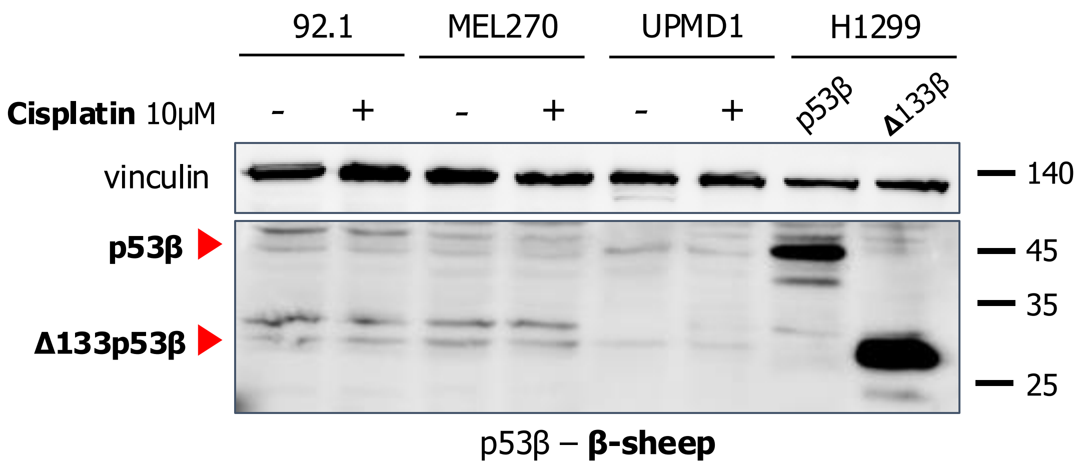

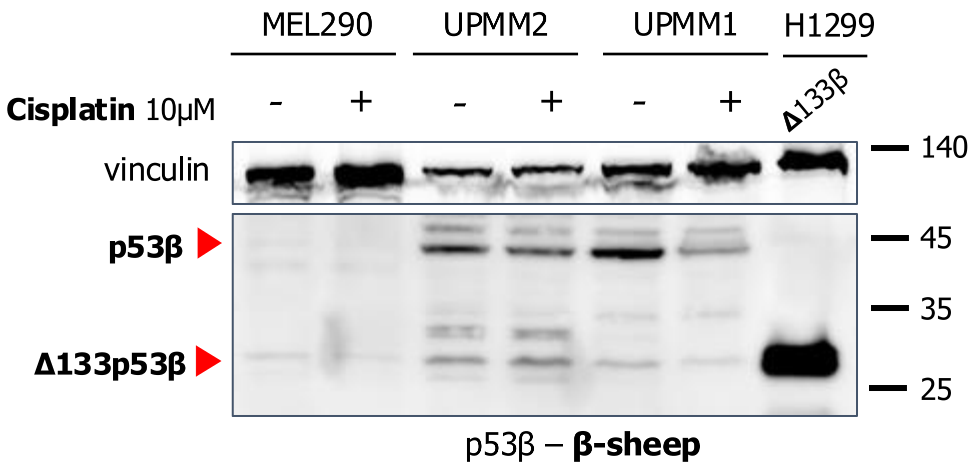

Supplementary Figure 8A-B

92.1-MEL270-UPMD1-H1299

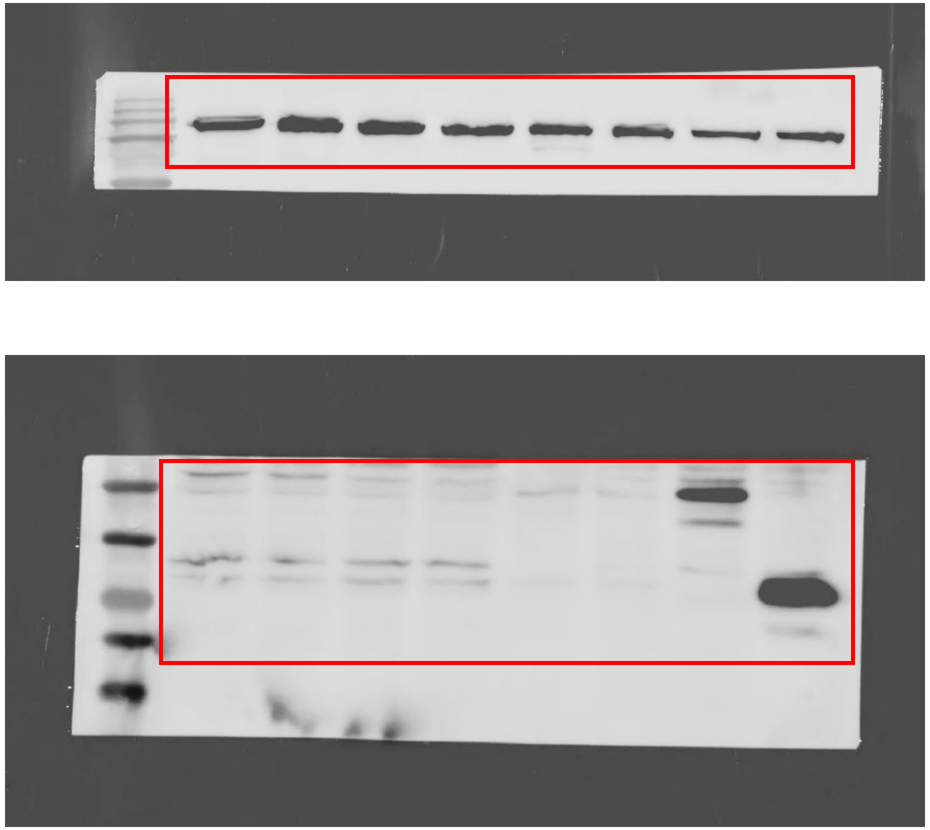

MEL290-UPMM2-UPMM1-H1299

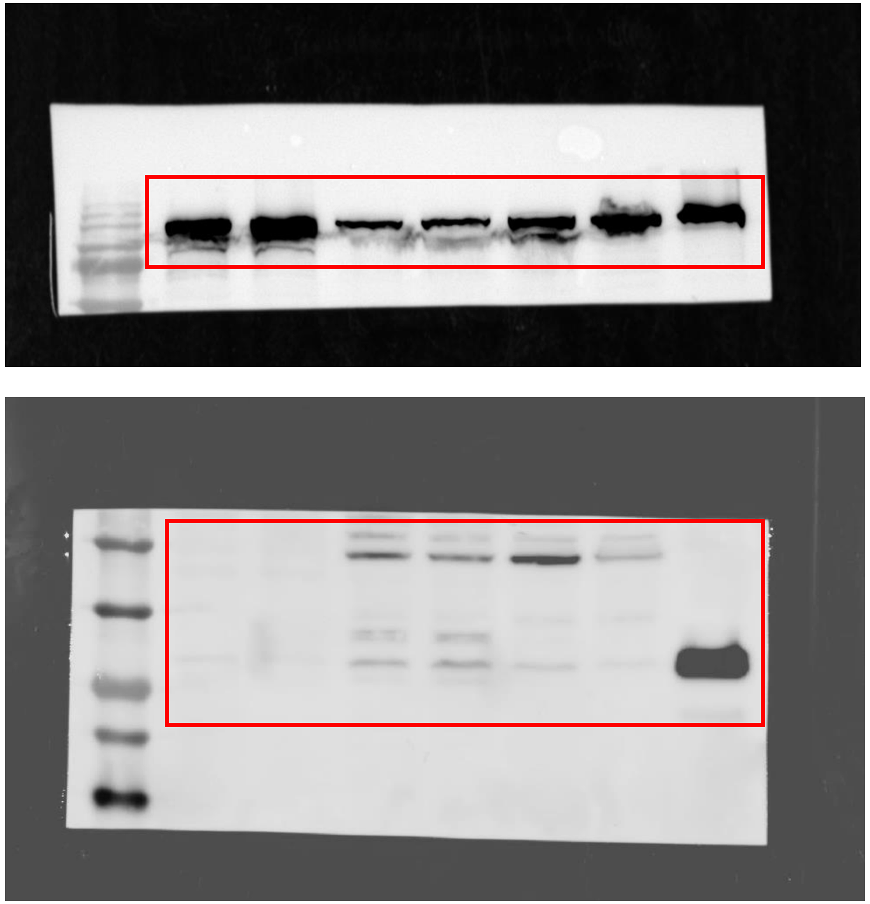

Supplementary Figure 8C-D

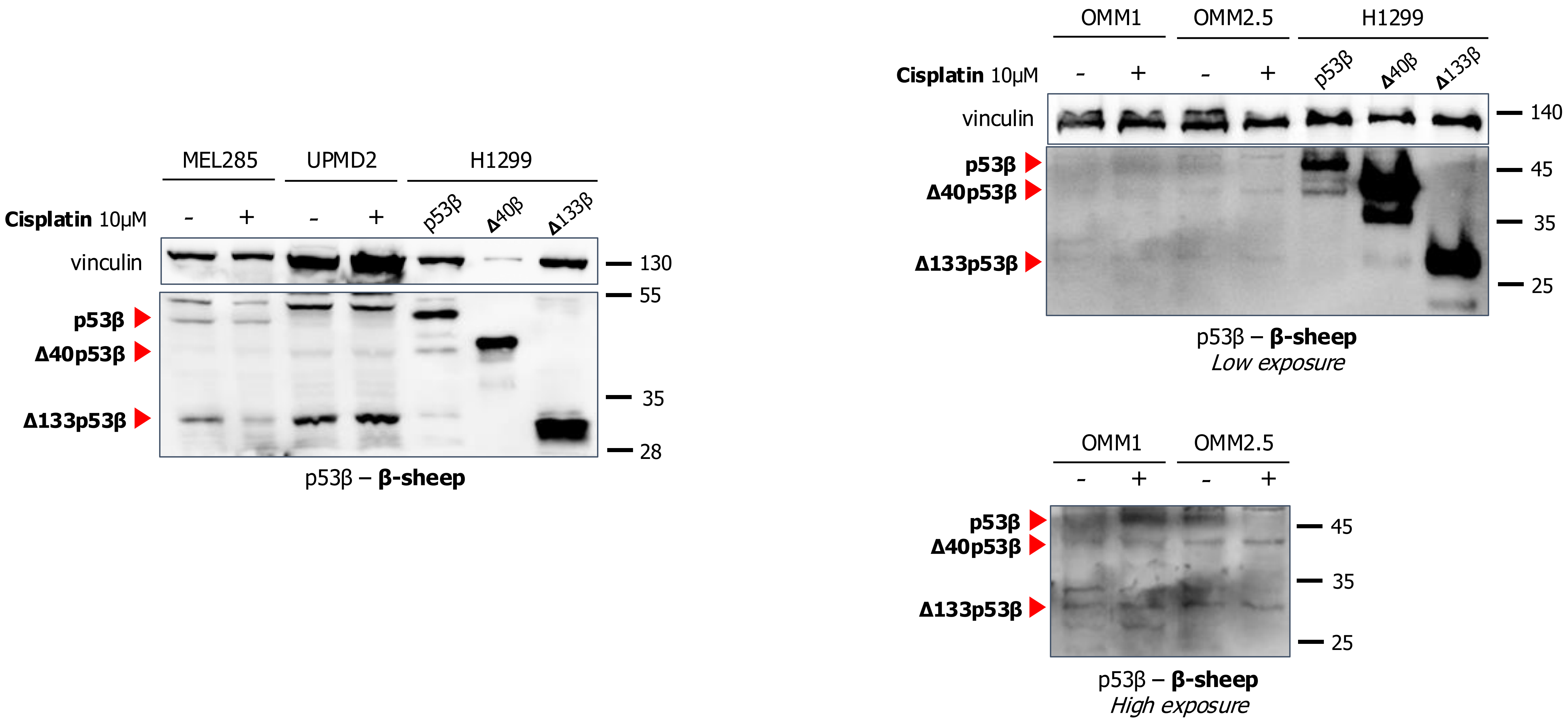

Supplementary Figure 8C-D

MEL285-UPMD2-H1299

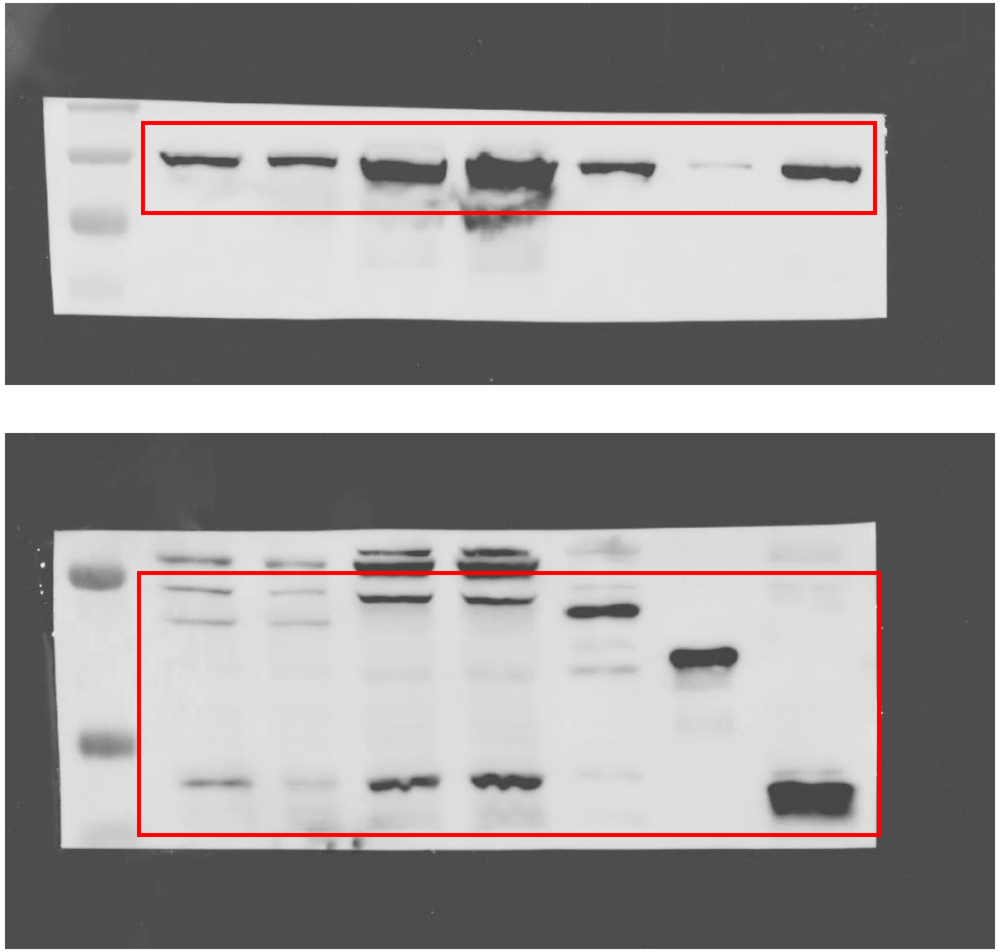

OMM1-0MM2.5-H1299

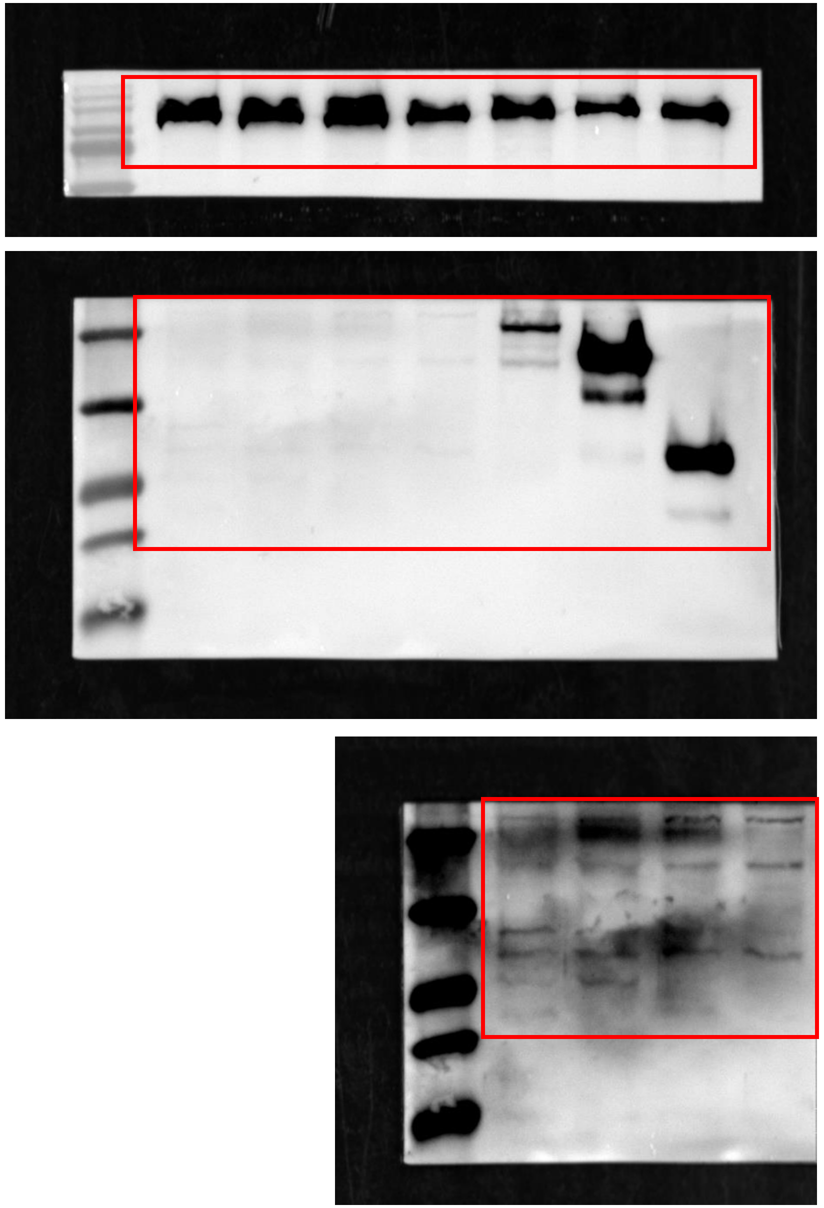

Supplementary Figure 8E-F

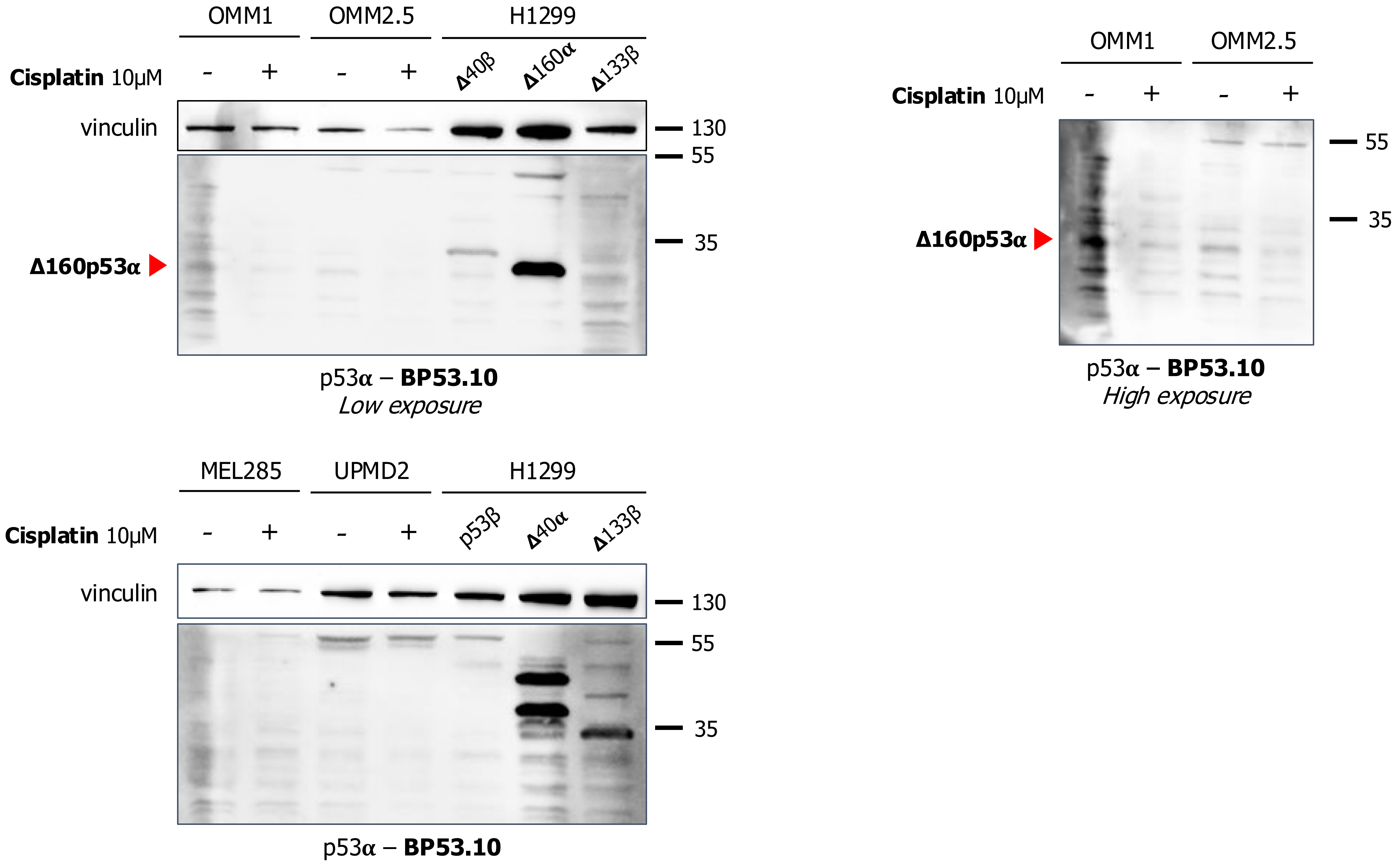

Supplementary Figure 8E-F

OMM1-0MM2.5-H1299

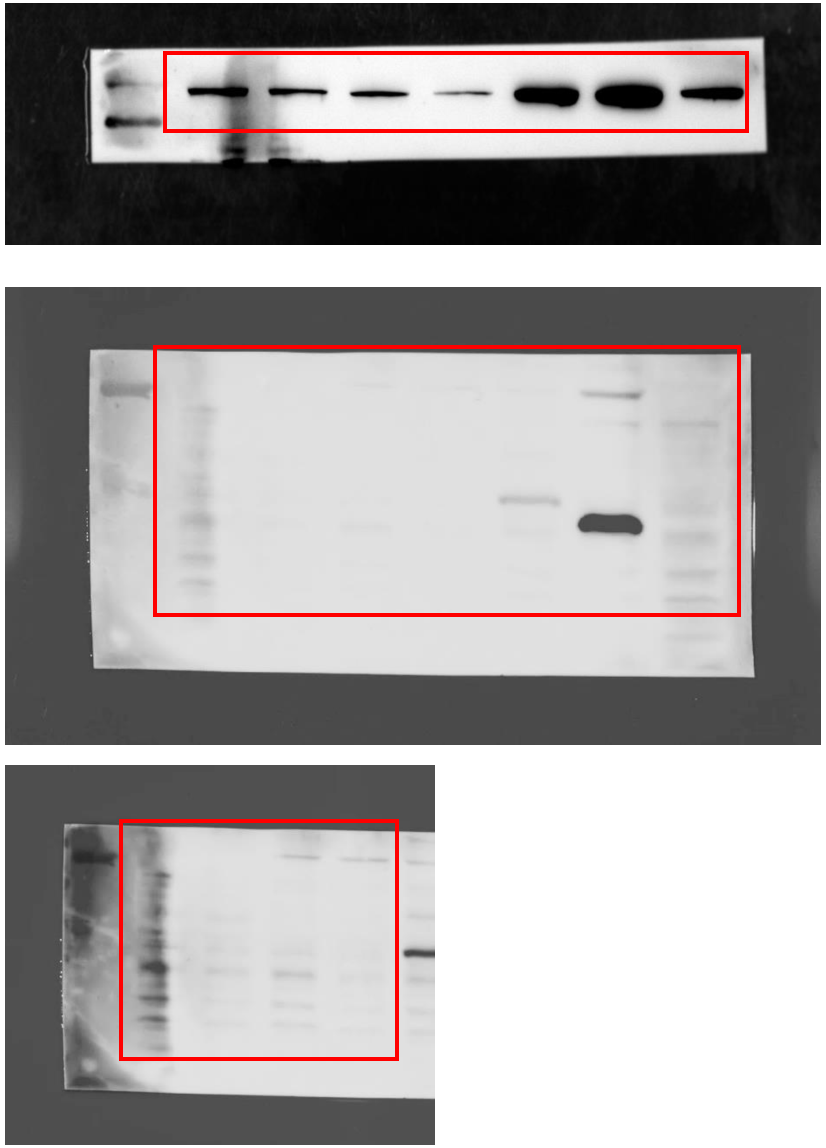

MEL285-UPMD2-H1299

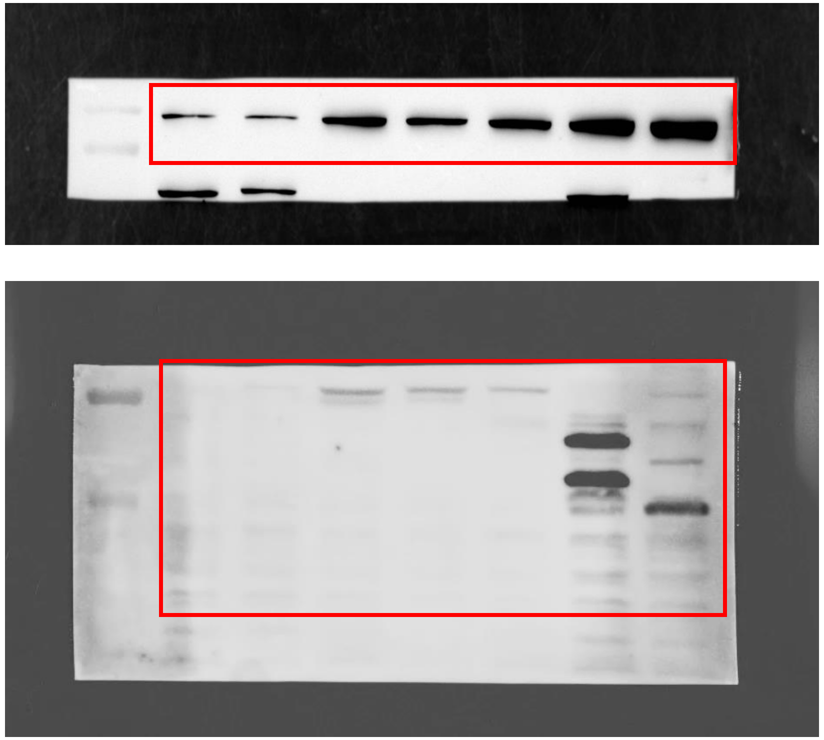

Supplementary Figure 9

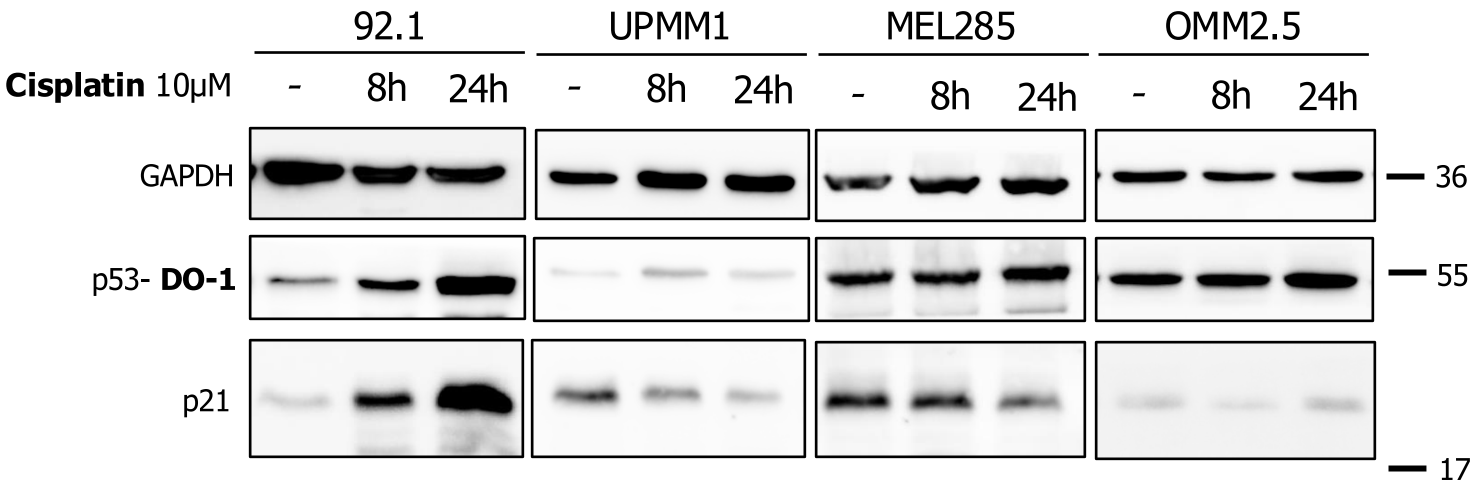

Supplementary Figure 9

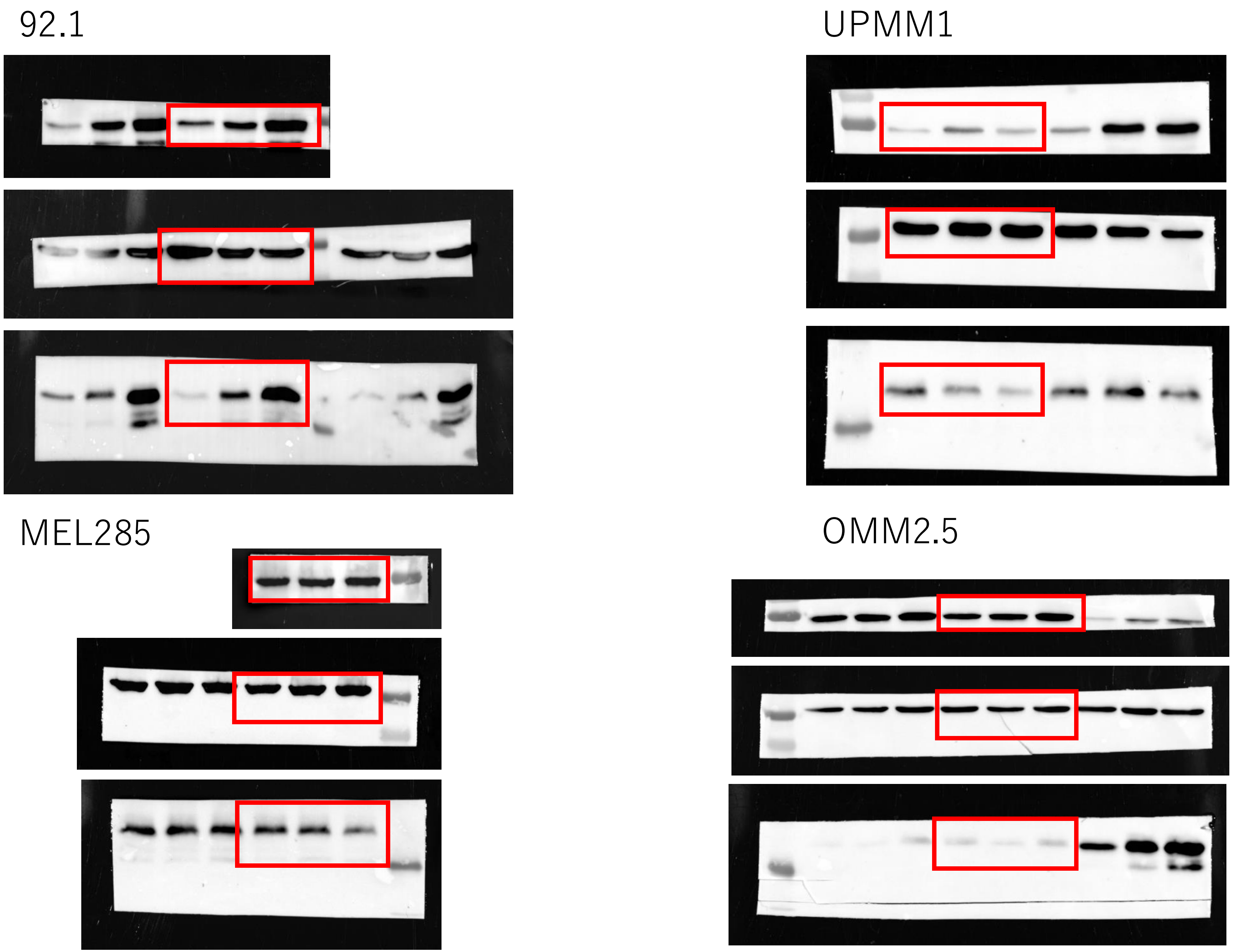

Supplement: Supplementary file 19 — Original data [file 41420_2025_2891_MOESM19_ESM.pdf]
